# *In Silico Oncology*: Quantification of the *In Vivo* Antitumor Efficacy of Cisplatin-Based Doublet Therapy in Non-Small Cell Lung Cancer (NSCLC) through a Multiscale Mechanistic Model

**DOI:** 10.1371/journal.pcbi.1005093

**Published:** 2016-09-22

**Authors:** Eleni Kolokotroni, Dimitra Dionysiou, Christian Veith, Yoo-Jin Kim, Jörg Sabczynski, Astrid Franz, Aleksandar Grgic, Jan Palm, Rainer M. Bohle, Georgios Stamatakos

**Affiliations:** 1 *In Silico* Oncology and *In Silico* Medicine Group, Institute of Communication and Computer Systems, National Technical University of Athens, Athens, Greece; 2 Institute of Pathology, University of Saarland, Homburg (Saar), Germany; 3 Philips Research, Hamburg, Germany; 4 Department of Nuclear Medicine, University of Saarland, Homburg (Saar), Germany; 5 Department of Radiotherapy and Radiation Oncology, University of Saarland, Homburg (Saar), Germany; University of California Irvine, UNITED STATES

## Abstract

The 5-year survival of non-small cell lung cancer patients can be as low as 1% in advanced stages. For patients with resectable disease, the successful choice of preoperative chemotherapy is critical to eliminate micrometastasis and improve operability. *In silico* experimentations can suggest the optimal treatment protocol for each patient based on their own multiscale data. A determinant for reliable predictions is the a priori estimation of the drugs’ cytotoxic efficacy on cancer cells for a given treatment. In the present work a mechanistic model of cancer response to treatment is applied for the estimation of a plausible value range of the cell killing efficacy of various cisplatin-based doublet regimens. Among others, the model incorporates the cancer related mechanism of uncontrolled proliferation, population heterogeneity, hypoxia and treatment resistance. The methodology is based on the provision of tumor volumetric data at two time points, before and after or during treatment. It takes into account the effect of tumor microenvironment and cell repopulation on treatment outcome. A thorough sensitivity analysis based on one-factor-at-a-time and latin hypercube sampling/partial rank correlation coefficient approaches has established the volume growth rate and the growth fraction at diagnosis as key features for more accurate estimates. The methodology is applied on the retrospective data of thirteen patients with non-small cell lung cancer who received cisplatin in combination with gemcitabine, vinorelbine or docetaxel in the neoadjuvant context. The selection of model input values has been guided by a comprehensive literature survey on cancer-specific proliferation kinetics. The latin hypercube sampling has been recruited to compensate for patient-specific uncertainties. Concluding, the present work provides a quantitative framework for the estimation of the *in-vivo* cell-killing ability of various chemotherapies. Correlation studies of such estimates with the molecular profile of patients could serve as a basis for reliable personalized predictions.

## Author Summary

Less than 14% of medically treated patients with locally advanced and metastatic non-small cell lung cancer are expected to be alive 5 years after diagnosis. Standard therapeutic strategies include the administration of two drugs in combination, aiming at shrinking the tumor before surgery and improving overall survival. Knowing the sensitivity profile of each patient to different treatment strategies at diagnosis may help choose the most appropriate ones. We develop a methodology for the quantitative estimation of the cytotoxic efficacy of cisplatin-based doublets on cancer cells by applying a simulation model of cancer progression and response. The model incorporates the proliferation cycle, quiescence, differentiation and loss of tumor cells. We evaluate the effect of *in vivo* microenvironment of real tumors, as expressed by measurable tumor proliferation kinetics, such as how fast the tumor grows, the percentage of cells that are actively dividing, the resistance of stem cells, etc. on treatment outcome so as to derive more accurate estimates. A literature survey guides the selection of values. The methodology is applied to a real clinical dataset of patients. Correlation studies between the derived cytotoxicities and the patients’ molecular profile could lead to predictions of treatment response at the time of diagnosis.

## Introduction

Worldwide, lung cancer accounts for most cancer related deaths among both men and women [1]. Non-small cell lung cancer (NSCLC) represents the most common type [1]. The success of current treatment choices depends on the extent of the disease at diagnosis; however, overall prognosis remains poor. For locally advanced and metastatic NSCLC, accounting for more than a half of NSCLC incidence [2], the 5-year survival rate ranges between 14% and 1% [1].

The use of cisplatin in combination with another agent remains the standard of care in NSCLC [3]. For patients with resectable tumors, neoadjuvant chemotherapy can be proven particular beneficial in terms of operability, event-free survival, time to distant recurrence and overall survival [4]. However, if treatment fails, a considerable time will have passed during which the tumor may have advanced or even become inoperable [4]. Treatment choices have routinely been based on stage, tumor size, location, lymph node or distant metastasis and overall health status. The exploitation of the molecular profile of cancer cells as treatment selection criteria in NSCLC has only been limited to the consideration of EGFR or ALK mutations as therapeutic targets [5–6]. However, the consideration of the molecular landscape, not to mention the complete genome sequencing, of cancer cells to guide treatment choice is a promising new research area in the field of personalized medicine [7–8].

Mechanistic models that summarize our knowledge on cancer progression are potential candidates to bridge the gap between the gene and molecular world and the prediction of treatment failure or success. Simplified models summarizing cancer biology in few parameters (e.g. growth rate) cannot grasp the great inter-patient and intra-tumor heterogeneity of the disease. Personalized cancer treatment demands for more sophisticated approaches to better trace and understand the biology of cancer and overcome the barriers of current therapeutic practices. The success of such a model to predict the therapeutic outcome depends greatly on the accuracy of the patient-specific estimates of its parameters at the time of diagnosis.

In the present work a methodology for the estimation of *in vivo* cell killing ability of chemotherapy based on data routinely measured in clinical practice has been developed. A discrete event-discrete state, clinically oriented simulation model of tumor response to chemotherapy is applied to translate tumor shrinkage after treatment to cytotoxic efficacy by taking into account the effect of tumor kinetics on treatment outcome. Measure of the antitumor efficacy is the ‘cell kill rate’ that expresses the fraction of treatment-induced cell losses, i.e. the fraction of cancer cells that are lethally hit, after each drug administration. The model addresses primary tumors during their clinical course of life, well beyond their initiation phase. It has been designed to incorporate patient-specific data such as imaging-based, histological, molecular and treatment data. In our modelling approach the high complexity of cancer is reduced to these essential mechanisms that prevail at cellular and super-cellular length scales. Hypoxia due to an insufficient tumor neovasculature, reversible dormancy, active proliferation and spontaneous, starvation-induced or treatment-induced cell loss are among the biological processes considered. Intra-tumor heterogeneity has been implemented via the cancer stem cell (CSC) hypothesis. Different resistance profiles of cancer cells have been taken into account. An essential feature of the model is its capability to simulate different proliferation patterns based on the assumed conditions of tumor microenvironment. Eventually tumor progression is regulated by the interplay of the above considered biological mechanisms.

The core simulation model of tumor cell multiplication stems from previous work of the *In Silico* Oncology-*In Silico* Medicine Group, ICCS, National Technical University of Athens [9–12]. In the present work, several extensions and adaptations have been performed to account for the modelling of the combination chemotherapy and the mechanism of action of the drugs considered. Furthermore, by recruiting statistical sampling methods to overcome intrinsic uncertainties in model parameter values, a methodological framework has been proposed that enables the application of mechanistic simulation models on the retrospective analysis of accumulated clinical data in a systemic way.

In order to facilitate the interpretation of histological subtypes to model parameters, a comprehensive literature survey on non-small cell lung cancer-specific proliferation kinetics has guided the consideration of biologically-reasonable and cancer-specific value ranges for critical proliferation features.

Moreover, in terms of better understanding cancer, the effect of key biological processes that drive tumor progression on treatment response are studied and clinical implications are discussed. More specifically, a thorough sensitivity analysis has been performed to decipher which tumor features are determinant of the quantitative estimation of the drugs’ cell killing ability. The tumor features under examination include tumor growth rate, cell cycle time, growth fraction, cell loss and chemo-resistance. Means to improve the accuracy of our estimations by exploiting measurable tumor proliferation kinetics are discussed.

Finally, a crucial advancement in relation to previous works is the use of real anonymized clinical data. The methodology is applied and early validated in the case of neoadjuvant chemotherapy treatment of NSCLC with various combinations of the antineoplastic agent cisplatin with gemcitabine, vinorelbine, and docetaxel. A real clinical dataset of 13 patients with primary NSCLC has been recruited for the purposes of this study. The anonymized data originate from the Institute of Pathology and the Clinical Cancer Registry, University Hospital of Saarland, Germany.

## Methods

### Patients

Thirteen patients with newly diagnosed NSCLC are included in the study (Table 1). The patients were treated at the Institute of Pathology of the University Hospital of Saarland between 2006 and 2010. The patients were between 45 and 67 years of age. Nine of the patients were diagnosed with squamous cell carcinoma (SCC) and the rest four with adenocarcinoma (ADC). None of the patients had previous cancer occurrence. All patients had stage III or IV disease according to TNM Classification of Malignant Tumours, 7th ed. There were two patients with distant metastases at the time of diagnosis. All patients received primary systemic chemotherapy prior to surgery.

**Table 1:** Patient characteristics.

Characteristic	n (%)
Number of cases	13
Age (Mean)	56
Sex	
Male	11 (85)
Female	2 (15)
Histology	
ADC	4 (31)
SCC	9 (69)
Differentiation grade	
Well	1 (8)
Moderate	2 (15)
Poor	5 (38.5)
Unknown	5 (38.5)
T-classification	
1	3 (23)
2	6 (46)
3	3 (23)
4	1 (8)
N-classification	
0	4 (31)
1	2 (15)
2	7 (54)
M-classification	
0	11 (85)
1	2 (15)
Stage	
III	10 (77)
IV	3 (23)
EGFR mutations	
Yes	1 (8)
No	12 (92)
KRAS mutations	
Yes	1 (8)
No	12 (92)
Treatment	
Cisplatin & Gemcitabine	3 (23)
Cisplatin & Docetaxel	1 (8)
Cisplatin & Vinorelbine	9 (69)

All data exploited by the present study have been provided, following anonymization, through the security framework implemented within the Contra Cancrum European Commision-funded program [13] (Contra Cancrum: Clinically Oriented Translational Cancer Multilevel Modeling’, FP7-ICT-2007-2-223979, http://contracancrum.eu/). Pseudonomized data from the patients and tumor tissue of the Contra Cancrum project were used according to the approval of the ethical committee Ethik-Kommission, Ärztekammer des Saarlandes, Faktoreistr. 4, 66111 Saarbrücken, Germany—approval number: 104/10. (This Committee is responsible for any medical ethics decision in the state of Saarland including all affairs of the medical faculty of the University of Saarland.) As the study has to focus on retrospective data e.g. tissue data from stored tissue samples the ethical committee agreed that informed consent cannot be gained in retrospective cases.

### Treatment schedule

All patients were treated preoperatively with a cisplatin-based doublet regimen (Table 2): one patient received a combination therapy with cisplatin (75 mg/m^2^) plus docetaxel (75 mg/m^2^), three patients received cisplatin (80 mg/m^2^) plus gemcitabine (1250 mg/m^2^), and nine patients were given cisplatin (80 mg/m^2^) plus vinorelbine (30 mg/m^2^). The cisplatin/gemcitabine and cisplatin/vinorelbine doublet regimens were given as a three-week cycle, administered two or three times. On day 1 of each cycle the patients received a short IV infusion (10-30min) of gemcitabine (or vinorelbine), followed by a 1–2 h IV infusion of cisplatin, whereas on day 8 only gemcitabine (or vinorelbine) was administered. In the case of cisplatin/docetaxel doublet regimen, both chemotherapeutic agents (1h IV infusion of docetaxel followed by 1h IV infusion of cisplatin) were given on day 1 of a 21-day cycle, repeated three times. After systemic chemotherapy, the patients underwent surgical resection of the primary tumor and/or the regional lymph nodes.

**Table 2:** Summary of clinical data.

Case #	Histologic type*	Tumor volume (mm^3^) at initial CT acquisition	Tumor volume (mm^3^) at second CT acquisition	Relative volume reduction (% of initial volume)	Interval (days) between CT acquisitions	Interval (days) between initial CT and treatment onset	Drug administrations (day)^‡^
***Cisplatin (80 mg/m***^***2***^***) & Gemcitabine (1250 mg/m***^***2***^***)***							
1	SCC	100264	46776	53.35	47	5	GEM:1^st^, 8^th^, 22^nd^, 29^th^, CIS:1^st^, 22^nd^
2	SCC	568264	168048	70.43	46	4	GEM:1^st^, 8^th^, 22^nd^, 29^th^_,_ CIS:1^st^, 22^nd^
3	SCC	101216	26336	73.98	45	4	GEM:1^st^, 8^th^, 22^nd^, 30^th^, CIS:1^st^, 22^nd^
***Cisplatin (75 mg/m***^***2***^***) & Docetaxel (75 mg/m***^***2***^***)***							
4	SCC	41376	18352	55.65	67	27	DOC: 1^st^, 22^nd^, CIS:1^st^, 22^nd^
***Cisplatin (80 mg/m***^***2***^***) & Vinorelbine (30 mg/m***^***2***^***)***							
5	SCC	20760	5824	71.95	90	47	VIN: 1^st^, 8^th^, 22^nd^, 29^th^, CIS:1^st^, 22^nd^
6	SCC	41056	23416	42.97	63	14	VIN: 1^st^, 8^th^, 22^nd^, 29^th^, 43^rd^, CIS:1^st^, 22^nd^, 43^rd^
7	SCC	39776	15840	60.18	42	-1	VIN: 1^st^, 22^nd^, 29^th^, CIS: 1^st^, 22^nd^
8	SCC	133864	29816	77.73	47	5	VIN: 1^st^, 8^th^, 22^nd^, 29^th^, CIS:1^st^, 22^nd^
9	SCC	123016	131160	-6.62	83	27	VIN: 1^st^, 8^th^, 29^th^, 36^th^, CIS: 1^st^, 29^th^
10	ADC	45160	8544	81.08	69	27	VIN: 1^st^, 8^th^, 22^nd^, 29^th^, CIS: 1^st^, 22^nd^
11	ADC	63136	20632	67.32	46	0	VIN: 1^st^, 8^th^, 26^th^, 33^rd^, CIS:1^st^, 26^th^
12	ADC	99872	51752	48.18	60	18	VIN: 1^stt^, 8^th^, 22^nd^, 29^th^, CIS:1^st^, 22^nd^
13	ADC	111744	29264	73.81	104	21	VIN: 1^st^, 8^th^, 36^th^, 43^rd^, 64^th^, 71^st^, CIS: 1^st^, 36^th^, 64^th^

*SCC: Squamous cell carcinoma, ADC: Adenocarcinoma

^**‡**^ Day 1 is treatment onset. Only the drug administration time points in the interim between the two CT acquisitions are recorded. Only these time points have been considered for the quantification of treatment efficacy.

### CT image acquisition

The CTs were performed by positioning the patients in supine position with both arms above the head. A spiral CT of the chest with intravenous contrast during mid–breath hold was obtained (two-channel Elscint Twin Flash CT or four-channel MX 8000 [Philips Medical Systems, Best, The Netherlands]) if there were no contraindications. A pitch value of 1.2 was used. Imaging data were reconstructed at 3mm slice thickness and 1.5mm reconstruction interval. All images were evaluated by a board-certified radiologist for the presence or absence of pathological lesions.

### CT image processing and volumetric methods

In order to be able to compare model predictions qualitatively or quantitatively with patient 3D CT images, the images taken at different time instances must be properly aligned (registration) and the tumor shape must be determined (segmentation).

#### Registration

The task to register lung CT images taken at different time points is not trivial. The acquired CT images contain not only the tumor under investigation, but the whole thorax or even the whole body. For the purpose of tumor growth modelling, however, the alignment of these images is required to be accurate in a region around the tumor only, whereas in other body regions a larger registration error can be accepted. Therefore we decided to use a combination of a pre-computed fast global affine registration with an on-demand local block matching to identify corresponding lesions [14]. The user marks a lesion in the base-line CT image. The algorithm then finds the corresponding lesions in the follow-up scans automatically by processing three basic steps: 1. fast multi-resolution affine registration (scaling, shift) with cross-correlation as similarity measure [15], 2. block matching registration refinement of local volumes of interest (VOI) around the corresponding lesion positions, and 3. additional local lesion search in the corresponding PET scans in case of unsuccessful block matching [14].

#### Segmentation

The task to segment lung tumors in CT images in three dimensions is not trivial as well. There are many papers and methods on automatic segmentation of small lung nodules [14–16]. However, according to our experience these methods cannot be directly applied to the segmentation of larger lung tumors, which often have a substantial connectivity to other structures such as lung wall, hilum, or diaphragm. On the other hand, in model-based approaches as suggested in [17] the high variability in shape and size of lung tumors makes it difficult to define a general tumor surface model. Moreover, partial connection with the lung wall often occurs with little or no contrast between tumor tissue and outer-lung regions. Therefore we decided to apply a semi-automatic algorithm to segment the lung tumors, consisting of two steps:

1. *Interactive definition of initial tumor surface mesh*: We have developed a new technique for a fast, flexible, and intuitive 3D definition of initial tumor surface meshes. Points on the boundary of the tumor are marked by mouse clicks. After each mouse click a sphere is computed which best approximates the user-defined points. All points are projected onto this sphere, and the distances to the sphere are computed. These distances are then interpolated using radial basis functions, resulting in an interpolating deformed sphere through the user-defined points. If the user is not satisfied with the result, additional surface points can be defined, and the interpolation by radial basis functions is started again. Since the boundary points are defined completely manually, this definition of the initial surface mesh does not suffer from the drawbacks of existing automatic segmentation methods.
2. *Model adaptation*: To improve the accuracy of the result from the previous step, model based segmentation methods [18, 19] are applied. The deformed sphere from the previous step is converted into a triangular mesh. The adaption is an iterative process optimizing the influence of shape constraints, given by a triangular mesh, and features of the grayscale image in each step. These constraints are modeled by an energy term, forcing the mesh to adapt to characteristic features of the underlying image while restricting the deformation of the mesh.

### Exploitable patient data

The patient specific data that have been exploited by the model are the applied chemotherapeutic scheme (drugs, administration instants) and the 3D image of the tumor as segmented from CT imaging data (Table 2). The image processing of the initial DICOM files was implemented as described above. Interactive segmentation was performed at soft tissue window in the present study. The sets of imaging data refer to the primary tumor and were provided for two time instances before, and during or after the completion of the systemic treatment (prior to surgery). The reconstructed images contain information only about the external boundary of the tumor, whereas information concerning any distinct internal metabolic region is absent. Accordingly, the virtual tumor implemented is assumed homogeneous with a shape compliant to the segmented tumor shape.

### Mathematical model of treatment response

A detailed description of the model’s formulation can be found in [11–12]. The model is built on the concept of a discrete time and space stochastic cellular automaton. In particular, the anatomical region of interest is represented by a regular grid of voxels called “geometrical cells” (GCs). Each GC that belongs to the tumor region is occupied by a cluster of tumor cells that are distributed in various states, representing cell types and phases. The rules that govern the transition of tumor cells between these states, in each GC, are described by the cell kinetic model as presented below. A second set of rules regulate the movement of tumor cells between GCs, aiming at a spatial evolution conformal to the initial shape of the tumor [12].

**Fig 1:**
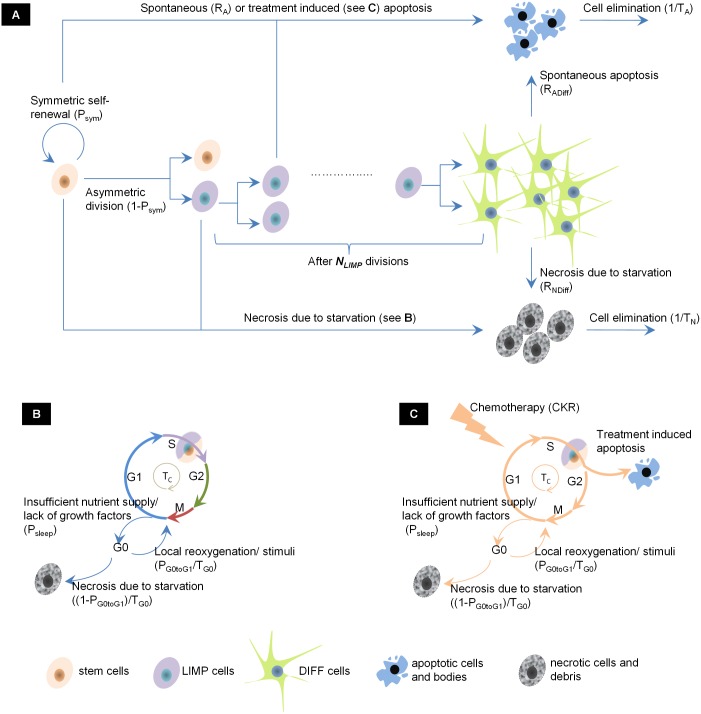
Generic cell kinetic model for tumor response to chemotherapy. (A) Transition diagram between the five main cancer cell categories. (B) Cell cycle of cancer cells with proliferative capacity, either stem or LIMP (C) Cell cycle of cancer cells with proliferative capacity that are lethally hit by chemotherapy. Cells enter a rudimentary cell cycle that leads to apoptotic death from the phase dictated by the mechanism of action of the chemotherapeutic drug. In the schema, lethally hit cells are assumed to die at the end of S phase. Parameter symbols are explained in Table 4. Abbreviations: LIMP: LImited Mitotic Potential tumor cell (also called committed or restricted progenitor cell), DIFF: terminally DIFFerentiated tumor cell, G1: Gap 1 cell cycle phase, S: DNA synthesis phase, G2: Gap 2 phase. M: Mitosis phase, G0: dormant, resting phase.

Tumor propagation is modeled based on the ‘cancer stem cell’ hypothesis and is regulated by the balance between active cell cycle, quiescence, differentiation and death. The tumor population comprises the following five cancer cell categories: a. cancer stem cells, which possess an unlimited mitotic capacity, b. cells of limited mitotic potential (LIMP), c. terminally differentiated cells (DIFF) that have lost the ability to divide, d. cells that have died through apoptosis and e. cells that have died through necrosis. In addition, stem and LIMP cells can be either cycling, distributed in the four phases of the cell cycle (G1, S, G2, M), or quiescent (G0). The rules that describe the transition between the various categories/phases of the cancer cells are depicted in Fig 1. On the top of the developmental hierarchy lie the stem cells that have the ability of self-renewal and differentiation. Two types of stem cell division are allowed: symmetric that gives rise to two stem cells, with probability *P*_*sym*_, and asymmetric that gives rise to one stem and one LIMP cell, with probability 1-*P*_*sym*_. LIMP cells follow a type of aberrant differentiation pathway. After *N*_*LIMP*_ divisions, LIMP cells are assumed to generate the population of the DIFF cells. Cycling cells (stem or LIMP) require a time equal to *T*_*C*_ in order to progress throughout the active cell cycle. Cellular dormancy is considered to be due to both nutrient deprivation (hypoxia) and lack of growth-promoting stimuli. Proliferating cells found under either one of the aforementioned conditions are assumed to withdraw from the active cycle into a common G0 state upon completion of mitosis, with a mean probability of *P*_*sleep*_. Under conditions of insufficient nutrient supply and oxygenation, quiescent cells can survive for a limited period. Subsequently, they die through necrosis unless the local metabolic conditions stimulate the entry into the cell cycle. A mean residence time of cells in G0 phase equal to *T*_*G0*_ is considered. After its expiration cells reenter cell cycle with a mean probability *P*_*G0toG1*_. Cycling and quiescent cells may die through spontaneous apoptosis, with rate *R*_*A*_. Differentiated cells may die through apoptosis (rate *R*_*ADiff*_) or necrosis (rate *R*_*NDiff*_). Apoptotic and necrotic cells are assumed to be present in the tumor bulk for a time length *T*_*A*_ and *T*_*N*_ respectively, before their final elimination.

In our modelling approach, chemotherapy is assumed to affect only cancer cells with proliferative capacity, either cycling or quiescent, depending on the cell cycle specificity of the drug. The activation of apoptosis is regarded as the major mode of action of chemotherapeutic drugs against cancer cells at clinical relevant doses. Chemotherapy-induced apoptosis is implemented through the parameter ‘cell kill rate’ (CKR) that expresses the fraction of cancer cells that sustain lethal damage by the drug(s) and are destined to die. These cells enter a rudimentary cell cycle before the triggering of the apoptotic pathway. The exact time point within the cell cycle when lethally hit cells enter the apoptotic compartment depends on the mechanism of action of the specific drug. We assume that all lethal cell lesions induced by the drug take place instantaneously at the time of drug administration, and disregard any time delays due to the method of administration or the specific pharmacokinetics and pharmacodynamics of the drug. The consideration of such delays would postpone the permanent removal of lethally hit cells from several hours to a few days (S1 Text). Given that the time interval between the last drug administration and the final tumor volumetric measurement is comparatively long enough (Table 2), the bulk of lethally hit cells will have been removed and will not contribute to the final volume of the simulated tumor, in all case scenarios (S1 Text), despite the aforementioned delays. Likewise, since the dosage interval is comparatively long, no drug accumulation is anticipated between consecutive administrations of the drugs (S1 Text). Therefore, the consideration of such delays is not expected to alter the results of the present study with respect to the estimated CKR.

Lethal lesions are assumed to occur in all cell cycle phases, since the drug absorption or the damage may take place in other phases too, besides the specific phase during which the final toxicity is manifested. Indeed, for all of the drugs considered in the present study, their ability to kill cells in all proliferative phases has been reported in literature (Table 3). As a first simplified approximation, for cell cycle specific drugs (gemcitabine, vinorelbine and docetaxel), the same value of cell kill rate is assumed for all proliferative phases, corresponding to a mean fraction of cells that will be lethally hit by the drug over the entire cell cycle. For cell cycle non-specific drugs (cisplatin) the same value of cell kill rate is assumed for cell cycle and G0 phases. When drugs are used in combination, their effects are considered additive, i.e. the total number of cells that are lethally hit in each proliferative phase is determined by the sum of the cell kill rates of the drugs. The additive action is applicable only for the proliferative phases (G1, S, G2, M), since cisplatin is the only drug that acts out of the active cell cycle, i.e. during G0 phase. Table 3 summarizes the action mechanisms and the modeling approach for the drugs considered in the present study.

**Table 3:** Main mechanism of action of cisplatin, gemcitabine, vinorelbine and docetaxel.

	*Mechanism of action*	*Modeling approach*
***Cisplatin***	The cytotoxic effect of cisplatin (*cis*-DDP) on cancer cells has primarily been attributed to the formation of cisplatin-gDNA adducts (mainly l,2-d(GpG) and l,2-d(ApG) intrastrand cross-links) [56–58]. In an attempt to remove these adducts and restore DNA lesions, the cells undergo a transient S arrest and a more persistent G2/M arrest [59]. Failure of DNA repair mechanisms, in case of extended damage, activates a number of signal transduction pathways (e.g. p53 involved pathways, c-Abl- and p73-dependent cascade, mitogen-activated protein kinase pathways, protein kinase C pathway, regulation of Bcl-2 family proteins, calcium signaling, death receptor signaling, mitochondrial pathways and caspaces cascade) that lead to programmed cell death or apoptosis [56–58, 60–62]. Accumulating evidence, derived from various cancer cell lines including NSCLC, has linked the initiation of apoptotic pathway with the activity of mismatch repair proteins and the G2/M checkpoint [56–58, 63]. Lethally damaged cells seem to stay trapped, but viable, at G2/M phase for a few days and then proceed to death [63]; in addition, entrance to mitosis and realization of a final aberrant mitosis prior to death has been observed [64]. Even though, depending on dose, cellular status or the molecular profile of the cell, nonapoptotic cell death pathways, have been reported as well (e.g. a defective apoptotic program, necrosis [65] or mitotic catastrophe followed by necrosis [66]), apoptosis is accepted to be the dominant cell death mechanism induced by cisplatin [62]. *cis*-DDP is considered cell cycle nonspecific [67, 68].	Equally affects cycling (regardless of phase) and quiescent tumor cells. Lethally-hit tumor cells enter the apoptotic pathway at the end of G2 phase.
***Gemcitabine***	Studies with a wide spectrum of cell lines [69–72] have evinced apoptosis as the method of cell death induced by gemcitabine (dFdC). Inside the cell, dFdC undergoes a series of phosphorylations resulting in its active diphosphate (dFdC-DP) and triphosphate (dFdC-TP) forms. dFdC-induced apoptosis is primarily related with the incorporation of the triphosphate form into the DNA strand during replication, acting competitively with the normal mucleoside deoxycytidine triphosphate-dCTP [69, 72, 73]. The inhibition of chain elongation and the inability of proofreading polymerases to recognize and remove the erroneous nucleoside (mask chain termination), impair DNA synthesis and induce apoptosis [72, 73]. The above effect is self-potentiated by the function of dFdC-DP that inhibits ribonucleotide reductase, causing depletion of dCTP pool, and, hence, favors both dFdC phosphorylation and incorporation into DNA. Notably, in solid tumors, the cytotoxic effect of dFdC is not limited in the S-phase cells and the drug kill cells in all proliferative phases [71, 74]. Apoptosis is initiated several hours after exposure [71]. Moreover, the drug causes a G0/G1 and S phase arrest [71, 75]. dFdC is considered cell cycle specific [67, 68].	Affects only cycling tumor cells (regardless of phase). Lethally-hit tumor cells enter the apoptotic pathway at the end of S phase.
***Vinorelbine***	Vinorelbine, a member of the *Vinca* alkaloid class, is a microtubule-targeting agent. Microtubules are polymers involved in the formation of the mitotic spindle that pulls apart the sister chromatids during cell division. At relatively low, but clinically relevant, concentrations, vinorelbine can suppress microtubule dynamics (dynamic instability and treadmilling,) [76–78], whereas at higher concentrations it can prevent the polymerization of tubulin into microtubules, cause microtubule depolymerization or induce formation of tubulin paracrystals [76, 79]. In all cases, the subsequent disruption of mitotic splinde formation and function and, thus, the inhibition of chromosomal segregation leads to a prolonged arrest during mitosis [77, 79–81], and, eventually, apoptosis through the intrinsic mitochondrial apoptotic pathway [77, 78, 81, 82]. Even though vinorelbine is thought to act specifically during the M phase, its ability to induce cell death in all cell cycle phases, independently of mitotic arrest, has been demonstrated [83]. Vinorelbine is considered cell cycle specific [67, 68].	Affects only cycling tumor cells (regardless of phase). Lethally-hit tumor cells enter the apoptotic pathway at the end of M phase.
***Docetaxel***	Docetaxel is a second generation taxane. It binds to β- tubulin and, similar to Vinca alkaloids, it impairs microtubule dynamics; however, it does so by enhancing microtubulin assembly and, thus, by stabilizing microtubules against depolymerization [67]. As a consequence, cell cycle is arrested during mitosis and the cell dies. Even though various mechanisms of cell death in response to docetaxel have been reported in literature, e.g. mitotic catastrophe, aberrant mitosis, treatment-induced senescence, and lytic necrosis [84, 85], apoptosis is accepted to be the predominant mode of cell death induced by docetaxel at clinically relevant (relatively high) doses [86–88]. Two scenarios of taxane-induced apoptosis have been described, either directly from mitosis, after mitotic arrest, or following mitotic exit to G1 without division at a tetraploid state (mitotic slippage) [85, 88–91]. The lethal effect of docetaxel during interphase, maximal against S-phase cells and minimal against G1 cells, implies that the drug can cause damage to structures essential for the completion of division at various phases prior to mitosis [92]. The apoptosis pathway induced by docetaxel seems to be related to the phosphorylation and, hence, inactivation, of bcl-2 [93]. Docetaxel is considered cell cycle specific [67, 68].	Affects only cycling tumor cells (regardless of phase). Lethally-hit tumor cells enter the apoptotic pathway at the end of M phase.

In order to study the effect of stem cell-resistance to chemotherapy, the model allows the independent handling of stem and LIMP cell categories. More specifically, the resistance to chemotherapy can be adjusted separately for stem or LIMP cell categories, through the cell kill factor (CKF) parameter. In the current work we have assumed that the CKF of LIMP cells is always equal to 1, i.e. the number of LIMP cells that are lethally hit by chemo is defined by CKR solely. Furthermore, the cell cycle and the cell cycle phase durations can be defined separately for the stem and the LIMP cells.

Table 4 presents the input parameters of the simulation model and their range of values according to the conducted literature survey or based on logical assumptions supported by basic science or clinical experience.

**Table 4:** Model Parameters Related to Tumor Free Growth and Therapy.

Parameter symbol	Description	Units	Value range**	References
**CELL PHASE DURATIONS**				
T_C_[class*]	Cell cycle duration	hours	18–134	[94–103]
T_G0_[class*]	G0 (dormant phase) duration i.e. time interval before a dormant cell re-enters cell cycle or dies through necrosis	hours	90–1200	extension of [29]
T_A_[region^‡^]	Time needed for both apoptosis to be completed and its products to be removed from the tumor	hours	1–25	[32–34]
T_N_[region^‡^]	Time needed for both necrosis to be completed and its products to be removed from the tumor	hours	1–200	[35], estimation
**CELL CATEGORY/PHASE TRANSITION RATES AND FRACTIONS**^¦^				
R_A_	Apoptosis rate of living stem and LIMP cells, i.e. fraction of stem and LIMP cells dying through apoptosis per hour	hours^-1^	0–0.001	estimation
R_ADiff_	Apoptosis rate of differentiated cells, i.e. fraction of differentiated cells dying through apoptosis per hour	hours^-1^	0.0001–0.02	extension of [36–39]
R_NDiff_	Necrosis rate of differentiated cells, i.e. fraction of differentiated cells dying through necrosis per hour	hours^-1^	0–0.02	estimation
P_sym_[region^‡^]	Fraction of stem cells at mitosis that perform symmetric division	-	0–0.4	estimation based on [30, 31]
P_sleep_[region^‡^]	Fraction of stem and LIMP cells entering the G0 phase following mitosis	-	0–1	-
P_G0toG1_[class*][region^‡^]	Fraction of dormant (stem and LIMP) cells that re-enter cell cycle	-	0–1	-
**MISCELLANEOUS PARAMETERS**				
N_LIMP_	Number of mitoses performed by LIMP cells before becoming differentiated	-	8–24	estimation
**CHEMOTHERAPY PARAMETERS**				
T _chemo, adm_ [n][drug^†^]	Time point of nth drug administration, n = 1,…	days	-	clinical data
CKR[drug^†^]	Cell kill rate i.e. fraction of stem and LIMP cells lethally hit by the drug at each administration	-	0–1	-
CKF[class*]	Cell kill factor i.e. factor adapting cell kill rate to stem or LIMP cells	-	0–1 for stem, 1 for LIMP	-

*Defined separately for ***stem*** and ***LIMP*** cells (class: {stem, LIMP})

^**‡**^Defined separately for ***proliferating*** and ***necrotic*** region (region: {proliferating, necrotic})

^**†**^Defined separately for each drug administered

^**¦**^The parameters included under this term express fractions and, therefore, can theoretically take any value between zero and unity. Whenever possible this range has been narrowed based on logical assumptions supported by literature or basic science.

**Used for LHS/PRCC sensitivity analysis

### Sensitivity analysis

The effect of the proliferation kinetics of the tumor cells on the estimation of CKR has been explored. Measures of the kinetics of cell proliferation are the relevant parameter space of the model (Table 4) and the derived proliferation features of the simulated tumor, i.e. volume growth rate and tumor cell population constitution. The methodologies applied are based on the one-factor-at-a-time (OFAT) approach [20] and the Latin Hypercube Sampling/Partial Rank Correlation Coefficient (LHS/PRCC) sensitivity analysis [20, 21].

#### OFAT approach

Plots by varying one input parameter, while keeping the others at a baseline value, have been produced. The method has been applied for all model input parameters (Table 4). The output measure considered has been the value of the cell kill rate of cisplatin, *CKR*_*cis-DDP*_, plus a predefined constant value standing for the CKR of the second drug, *CKR*_*B*_, a sum that results in the target tumor volume reduction following chemotherapy treatment. The *CKR*_*cis-DDP*_ is determined using the optimization procedure described below.

The one-factor-at-a-time sensitivity measure (SM) utilized to quantitatively rank the strength of the relationship between the output measure and the model parameters is the 10% increase and decrease of inputs (±10%) [20]. Each input parameter is perturbed by 10% above and below its considered baseline value and the corresponding percentage change in the calculated CKR is recorded. The rest of the model parameters are kept at their baseline values. It should be noted that, for integer parameters, the rounding to the nearest integer may result to a small deviation from the ±10% variation of the input. Furthermore, for input parameters with considerably strong influence on output, for which the ±10% variation results in tumors beyond the boundary of biological relevant behavior in terms of sustainability of growth, a ±5% metric has been considered. All percentage changes in the output are then expressed in relation to ±1% variation of the input, by dividing with the percentage change of the input, according to the formulas:

$$SM_{\text{+\%}}=\frac{\left(CKR_{base+\%}-CKR_{base}\right)/CKR_{base}}{\left(p_{i,base+\%}-p_{i,base}\right)/p_{i,base}}$$ (1)

$$SM_{\text{-\%}}=\frac{\left(CKR_{base-\%}-CKR_{base}\right)/CKR_{base}}{\left(p_{i,base-\%}-p_{i,base}\right)/p_{i,base}}$$ (2)

where *p*_*i*,*base*_: the baseline value of the *i-*th parameter, *p*_*i*,*base*+(-)%_: the value of the *i-*th parameter 10% or 5% above (or below) its baseline value, *CKR*_*base*_: the calculated CKR with all parameters set at their baseline values, *CKR*_*base*+(-)%_: the calculated CKR with the *i-*th parameter, only, set at 10% or 5% above (or below) its baseline value. A positive correlation between an input parameter and the CKR is translated to a positive *SM*_+%_ and a negative *SM*_-%_. On the other hand, a negative correlation between an input parameter and the CKR is translated to a negative *SM*_+%_ and a positive *SM*_-%_. The highest the absolute value of *SM*, the strongest the correlation and, hence, the influence of the input parameter on the calculated CKR. This type of sensitivity analysis is considered as ‘local’, since it addresses sensitivity around individual values of model parameters.

It should be commented that due to the discrete, *monte carlo* nature of the simulation model, a small deviation in the output is observed, between runs with the same input parameter values. The choice of the ±10% metric (compared to the ±5%) ensures that the output change is higher than the aforementioned variation even for model parameters with a small effect on output.

An intrinsic shortcoming of the aforementioned sensitivity measure is that it does not consider the impact of parameter variability on the output. To overcome this, the sensitivity measure of each input parameter is weighted by a normalized measure of its variability. The measure of variability chosen here is the value range of the input parameter divided by its mean [22]. Due to absence of relevant data, the mean is calculated assuming a uniform distribution. A mean sensitivity score (SC) is derived by weighting the average of the sensitivity measures *SM*_+%_ and *SM*_-%_, according to the formula:

$$SC=\left|\frac{SM_{\text{+\%}}-SM_{-\%}}{2}\right|\times \left(\frac{p_{i,\text{max}}-p_{i,\text{min}}}{p_{i,mean}}\right)$$ (3)

where *p*_*i*,*max*_, *p*_*i*,*min*,_ and *p*_*i*,*mean*_: the maximum, minimum and mean value of the *i-*th parameter respectively.

#### LHS/PRCC analysis

OFAT is the most straightforward approach to determine parameter sensitivity, understand the relation between input and output and identify the more or less critical parameters of a model. However, this approach does not take into account the presence of correlations between inputs and cannot catch the effect on output of simultaneously varying more than one parameter. Sampling-based approaches are considered to be more appropriate for the study of models with a multidimensional input space. The Latin Hypercube Sampling method is considered to be an efficient tool used to generate a set of well distributed combinations of model parameter values [21]. It provides the best compromise between efficient coverage of input parameter space and realistic assessment of the combined effect of inputs on output, with a relatively small sample size.

LHS is run to produce *N* combinations of parameter values. Due to unavailability of relevant data, a uniform distribution over the value range considered, for all of the model parameters, has been assumed. However, in the absence of constraint conditions between the input parameters, a considerable large subset of the *N* combinations, gives biological non-relevant tumors that cannot sustain growth and shrink on their own. These combinations are excluded from the subsequent analysis. A further refinement then takes place by considering only those parameter combinations that give tumors with proliferation kinetics within the value range as defined by the literature survey. For this subset of *N*^*’*^ combinations the CKR of *cis*-DDP that results in the desired volume reduction is determined using the optimization procedure described below. For each model parameter, a scatter plot displaying the *N*^*’*^ values for the parameter under examination against CKR is produced. A uniform distribution of dots suggests no correlation between input and output. On the other hand, the closer the dots cluster to form shapes with negative or positive slopes, the stronger the relation between input parameter and output measure.

A partial rank correlation analysis of the Latin hypercube sample is subsequently performed in order to quantify and rank the correlation between inputs and output. A rank transformation has been applied to account for the effect of nonlinear but monotonic relationships between the inputs and output. Specifically, the values of inputs/output data are replaced by their ranks and the partial correlation coefficients, i.e. the correlation coefficients between each input and the output after controlling for the effect of the remaining inputs, are calculated on the rank transformed data. *P* < .01 has been assumed as statistically significant. The partial rank correlation analysis is considered a sampling-based global sensitivity method [21], because it takes into consideration the whole value distribution of model parameters via a generated sample.

The analysis has been performed using the Matlab toolbox. The built-in function ‘lhsdesign’ has been chosen to produce a latin hypercube sample, with values ranging between 0 and 1. For input parameters bounded in any range [*a*_*p*_, *b*_*p*_] other than [0, 1], the ‘lhsdesign’ output has been rescaled by applying the formula:

$$a_{p}+\left(b_{p}-a_{p}\right)*x_{p}$$ (4)

where *x*_*p*_ the vector of the *N* returned values for parameter *p*. For integer input parameters the rescaled values are rounded to the nearest integer. The rank transformation and the estimation of the partial correlation coefficients have been implemented by using the built-in functions ‘tiedrank’ and ‘partialcorr’ respectively.

### Definition of baseline parameter values used in OFAT sensitivity analysis

The aim is to define a set of model parameter values that corresponds to a cancer representative proliferation pattern in terms of volume doubling time, *T*_*d*_, growth fraction (GF), apoptotic cell fraction (AF), expressed as the apoptotic cell population out of total cells, necrotic cell fraction (NF), expressed as the necrotic cell population out of total cells, and stem cell fraction (SF), expressed as the stem cell population out of living cells. We consider the fraction of newborn cells that enter the G0 phase, *P*_*sleep*_, the necrosis rate of differentiated cells, *R*_*NDiff*_, the duration of apoptosis, *T*_*A*_, the duration of necrosis, *T*_*N*_, and the number of mitotic divisions performed by LIMP cells before becoming terminally differentiated, *N*_*LIMP*_, as the dependent parameters of this multi-constrained problem. The independent variables comprise the rest of the model parameters that regulate tumor proliferation pattern, i.e. cell cycle time, *T*_*C*_, the duration of G0 phase, *T*_*G0*_, the apoptosis rate of stem and LIMP cells, *R*_*A*_, the apoptosis rate of differentiated cells, *R*_*ADiff*_, and the symmetric division fraction, *P*_*sym*_, and the above mentioned tumor proliferation features: GF, *T*_*d*_, AF, NF and SF. Initially, biologically representative values, determined based on the cancer-specific cell kinetics and logical assumptions, are assigned to the independent input parameters *T*_*C*_, *T*_*G0*_, *R*_*A*_, *R*_*ADiff*_ and *P*_*sym*_. Following, the *P*_*sleep*_ value is computed so as to achieve the given *T*_*d*_:

$$P_{sleep}=\frac{1-e^{(a+R_{A})Tc}/\left(1+P_{sym}\right)}{1-\frac{P_{G0toG1}}{T_{G0}}/\left(a+R_{A}+\frac{1}{T_{G0}}\right)}$$ (5)

(derived from Eq (7) in [12])

*R*_*NDiff*_, is calculated in order to achieve the given GF:

$$R_{NDiff}=\frac{1-P_{sym}}{\frac{1}{A}\left(\frac{1}{GF}-1\right)-B}-a-R_{ADiff}$$ (6)

(derived from Eq (47) in S2 Text)

where

$$A=\frac{a+R_{A}}{e^{\left(a+R_{A}\right)T_{C}}-1}$$ (7)

$$B=\frac{1+P_{sym}}{a+R_{A}+\frac{1}{T_{G0}}}P_{sleep}$$ (8)

By taking into account that the ratio of apoptotic, *N*_*A*_, to proliferating, *N*_*p*_, population is given by:

$$\frac{N_{A}}{N_{P}}=\frac{AF}{GF}\frac{1}{Living/Total}=\frac{AF}{GF}\frac{1}{1-AF-NF}$$ (9)

*Τ*_*A*_, can been calculated based on Eq (13) in S2 Text:

$$T_{A}={\left(\frac{R_{A}}{\frac{N_{A}}{N_{P}}}+\frac{R_{A}}{\frac{N_{A}}{N_{P}}}\left(1+P_{sym}\right)P_{sleep}\frac{A}{a+R_{A}+\frac{1}{T_{G0}}}+\frac{R_{ADiff}}{\frac{N_{A}}{N_{P}}}\left(1-P_{sym}\right)\frac{A}{a+R_{ADiff}+R_{NDiff}}-a\right)}^{-1}$$ (10)

Finally, *Τ*_*Ν*_ has been determined for the given NF based on Eq (17) in S2 Text:

$$\frac{N_{N}}{N_{P}}=\frac{NF}{GF}\frac{1}{1-AF-NF}$$ (11)

$$T_{N}={\left(\frac{\frac{\left(1-P_{G0toG1}\right)}{T_{G0}}}{\frac{N_{N}}{N_{P}}}\left(1+P_{sym}\right)P_{sleep}\frac{A}{a+R_{A}+\frac{1}{T_{G0}}}+\frac{R_{NDiff}}{\frac{N_{N}}{N_{P}}}\left(1-P_{sym}\right)\frac{A}{a+R_{ADiff}+R_{NDiff}}-a\right)}^{-1}$$ (12)

The value of *N*_*LIMP*_ can be estimated based on Eq (46) in S2 Text:

$$\sum_{n=0}^{N_{LIMP}-1}\frac{2^{n}}{{\left(1+P_{sym}\right)}^{n+1}}=\frac{1}{\left(1-P_{sym}\right)\frac{N_{S}}{N_{L}}}$$ (13)

The ratio of stem to LIMP cells in Eq (13) can be derived based on SF, GF and ratio of dormant to proliferating cells given by Eq (5) in S2 Text:

$$\frac{N_{S}}{N_{L}}=\frac{SF}{\left(1+\frac{N_{G0}}{N_{P}}\right)GF-SF}$$ (14)

$$\frac{N_{S}}{N_{L}}=\frac{SF}{\left(1+\left(1+P_{sym}\right)P_{sleep}\frac{a+R_{A}}{\left(a+R_{A}+\frac{1}{T_{G0}}\right)}\frac{1}{\left(e^{(a+R_{A})Tc}-1\right)}\right)GF-SF}$$ (15)

The above methodology is applied for each NSCLC subtype considered, i.e. ADC and SCC. The derived two value sets of model parameters consist the two sets of baseline values used in the OFAT sensitivity analysis.

### Generation of a NSCLC subtype-specific LHS sample of parameter values used for CKR estimation

The Latin Hypercube Sampling method is used to generate a plausible collection of model parameter values that corresponds to virtual tumors having a common proliferation pattern in terms of *T*_*d*_, GF, AF and NF. We consider the fraction of newborn cells that enter the G0 phase, *P*_*sleep*_, the necrosis rate of differentiated cells, *R*_*NDiff*_, the duration of apoptosis, *T*_*A*_, and the duration of necrosis, *T*_*N*_, as the dependent parameters of this multi-constrained problem. The ‘lhsdesign’ function is run to produce *N* combinations of the independent model parameters: *T*_*C*_, *T*_*G0*_, *R*_*A*_, *R*_*ADiff*_, *P*_*G0toG1*,_ *P*_*sym*_, *N*_*LIMP*_, and *CKF* of stem cells. LHS output is modified in the case of parameters with value range other than [0, 1] and parameters of integer type, as previously described. For each combination of parameter values, parameters *P*_*sleep*_, *R*_*NDiff*_, *T*_*A*_ and *T*_*N*_, are derived from Eqs (5), (6), (10) and (12) as described previously, so as to achieve the given *T*_*d*_, GF, AF and NF. The above methodology is applied for each NSCLC subtype considered, i.e. ADC and SCC. The derived two LHS samples of parameter values are used for the estimation of the cell killing ability of the various cisplatin-based doublet regimens.

### CKR estimation

The cell kill rate of cisplatin, *CKR*_*cis-DDP*_, is adapted to the observed, in the case of clinical tumors, or assumed, in the case of sensitivity analysis, tumor size reduction. The adaptation of *CKR*_*cis-DDP*_ has been performed automatically using an optimization procedure (the built-in function ‘fzero’ of Matlab), that returns the value of the *CKR*_*cis-DDP*_ for which the difference between the observed or given volume reduction and the simulation outcome is zero. During this optimization procedure the rest of the model parameters, including the CKR of the second drug, are kept constant.

## Results

The structure of the ‘Results’ section is as follows. In the first subsection, the adequacy of the simulation model, in terms of a realistic representation of tumor dynamics, is presented. The second subsection investigates the sensitivity of CKR estimation with respect to simulation model parameters and various tumor proliferation features variations. The last subsections are dedicated to the estimation of treatment efficacy in terms of CKR for the 13 clinical cases and the early validation of the methodology. The proliferation features of NSCLC are described in S3 Text based on the findings of the literate survey. These findings have guided the selection of model parameter values for the sensitivity analysis and the CKR estimation of the real clinical cases.

### Pattern of tumor growth and response to treatment

The model supports the division of tumor volume into regions of distinct metabolic activity (i.e. necrotic, quiescent and proliferative). However, due to the lack of relevant info in the segmented tumor images, only a single metabolic region is considered. This implies that all model parameters affecting tumor cell kinetics have the same value throughout a tumor’s volume. Furthermore, the kinetics of tumor cell proliferation (i.e. phase durations and transition rates or fractions) are assumed constant throughout simulation, reflecting the mean values over the simulation time window. Such an approximation is considered applicable for a relatively short time interval compared to the tumor’s lifetime. Finally, the model aims at simulating fully developed, clinical tumors, well beyond their initiation phase. For such a biological system it is rational to assume that a state of population equilibrium and of balanced growth has been achieved. The methodology developed for the initialization of tumor cell populations [11–12] ensures conditions of balanced growth at the beginning of the simulation.

A tumor characterized by space and time invariant growth kinetics parameters and population equilibrium would demonstrate a grossly exponential growth pattern [12]. Even though it is generally accepted [23] that tumors grow in a Gompertzian manner during their lifetime, i.e. growth progressively slows down as the tumor enlarges, tumors that follow exponential law have been reported frequently in literature. Indeed, several lung cancer cases reported in literature exhibit fairly constant growth rates on the logarithmic scale [24–27] over prolonged periods of time, ranging from months to years. It has been argued that these tumors were probably still in the exponential phase of Gompertzian curve at the time of diagnosis and clinical surveillance [26].

**Fig 2:**
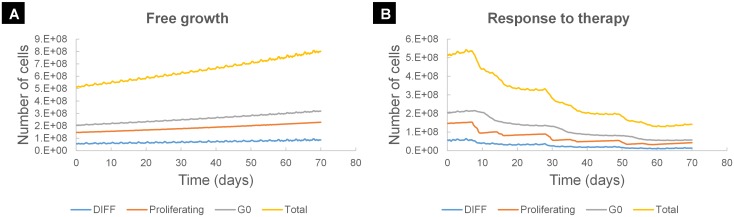
Simulated time course of selected cancer cell populations. (A) Various tumor cell populations as a function of time in the case of free tumor growth. A homogeneous spherical tumor of 10mm diameter is considered. The values of code input parameters that regulate tumor growth kinetics are given in Table 5 (Squamous Cell Carcinoma—SCC representative case). (B) Various tumor cell populations as a function of time in the case of treatment response. A homogeneous spherical tumor of 10mm diameter is considered. A cell cycle specific (gemcitabine) and a cell cycle non-specific (cisplatin) drug are administered as a three-week cycle. Gemcitabine is given on days 7, 14, 28, 35, 49, 56. Cisplatin is administered on days 7, 28 and 49. After each chemotherapeutic session a drop in the various tumor cell populations is observed, followed by tumor repopulation. The values of code input parameters are given in Table 5 (Squamous Cell Carcinoma—SCC representative case). Abbreviations: DIFF: terminally DIFFerentiated tumor cell, G0: dormant, resting phase.

Fig 2A and 2B show the simulated time course of a macroscopically homogeneous tumor under free growth or treatment conditions respectively. In the case of treatment a typical scheme for NSCLC has been assumed that is based on the combination of two chemotherapy drugs i.e. cisplatin and gemcitabine. The doublet regimen is administered three times as a three-weeks cycle. On the first day of treatment cycle the patient is given both gemcitabine and cisplatin. On the same day of the following week (day eight) only gemcitabine is administered. Qualitatively a fairly expected and reasonable tumor dynamics behavior can be easily noticed. An exponential pattern of growth in the absence of treatment and tumor regression followed by repopulation after each chemotherapeutic session in the case of treatment are successfully demonstrated.

### Effect of cellular proliferation kinetics on CKR evaluation: OFAT analysis results

Due to the lack of clinical data related to the presence of internal regions of distinct metabolic activity (necrotic, quiescent, proliferating), the analysis has focused on macroscopically homogeneous tumors, in the sense that all model parameters affecting tumor cell kinetics are spatially invariant throughout tumor volume. Moreover, the tumor is assumed to have a spherical shape of 925 mm^3^ initial volume (approximately 12mm initial diameter). A typical scheme has been considered that is based on the combination of two chemotherapy drugs i.e. cisplatin and gemcitabine. The doublet regimen is administered three times as a three-week cycle. On the first day of treatment cycle the patient is given both gemcitabine and cisplatin. On the same day of the following week (day eight) only gemcitabine is administered. The virtual tumor is allowed to grow for one week prior to treatment. A 72% treatment-induced shrinkage (final tumor equal to 258 mm^3^) is assumed on simulation day 49.

#### ADC and SCC baseline values

For the OFAT analysis two sets of baseline values have been specified, each one corresponding to a representative ADC and a representative SCC growth pattern (Table 5). The value sets have been obtained by adapting the model parameters to mean values of *GF*, *T*_*d*_, and fraction of stem, apoptotic and necrotic cells derived from literature (see Material and Methods). More specifically, the following assumptions/constraints were imposed:

1. A T_d_ equal to 225d for ADC and 109d for SCC corresponding to the average of median values reported in the reviewed literature for the two subtypes (Table A in S3 Text).
2. A GF equal to 0.18 for ADC and 0.36 for SCC corresponding to the average of median values reported in the reviewed literature for the two subtypes (Table B in S3 Text).
3. A cell cycle time within the range of the values reported in the reviewed literature (Table C in S3 Text).
4. A fraction of apoptotic cells (out of total cells) equal to 0.01 for both ADC and SCC corresponding to the average of median values reported in the reviewed literature (Table D in S3 Text).
5. A fraction of necrotic cells (out of total cells) equal to 0.02 for ADC and 0.20 for SCC—residing within the range of all values reported in the reviewed literature (Table E in S3 Text).
6. A fraction of stem cells (out of living cells) equal to 0.00005 for ADC and 0.0002 for SCC corresponding approximately to the average of frequencies reported in [28], for highly permissive xenotransplantation conditions (Table F in S3 Text).

**Table 5:** Baseline Values of Model Parameters Used in OFAT Sensitivity Analysis.

	ADC representative case	SCC representative case
**Model Parameter Values**		
T_C_ (h)	42	60
T_G0_ (h)	382	242
T_N_ (h)	23	79
T_A_ (h)	4	7
N_LIMP_	18	22
R_A_ (h^-1^)	0.0003	0.0001
R_ADiff_ (h^-1^)	0.008	0.017
R_NDiff_ (h^-1^)	0.0009	0.01
P_G0toG1_	0.5	0.1
P_sleep_	0.265733	0.27960
P_sym_	0.2	0.37
CKF of stem cells	0.5	0.5
CKR_dFdC_	0.2	0.2
Resulting proliferation kinetics of the simulated tumor*		
Doubling Time (d)	226.3	108.7d
Fraction of stem cells^‡^	0.000061	0.00021
Fraction of LIMP cells^‡^	0.62	0.86
Growth fraction^‡^	0.18	0.36
Fraction of dormant cells ^‡^	0.44	0.50
Fraction of DIFF cells^‡^	0.38	0.14
Fraction of apoptotic cells^†^	0.0093	0.012
Fraction of necrotic cells^†^	0.020	0.20

*Fractions rounded to two significant figures

^‡^out of living cells

^†^out of total cells

For the derivation of ADC and SCC baseline values, the same kinetics have been assumed for both stem and LIMP cell categories.

#### Cell cycle

**Fig 3:**
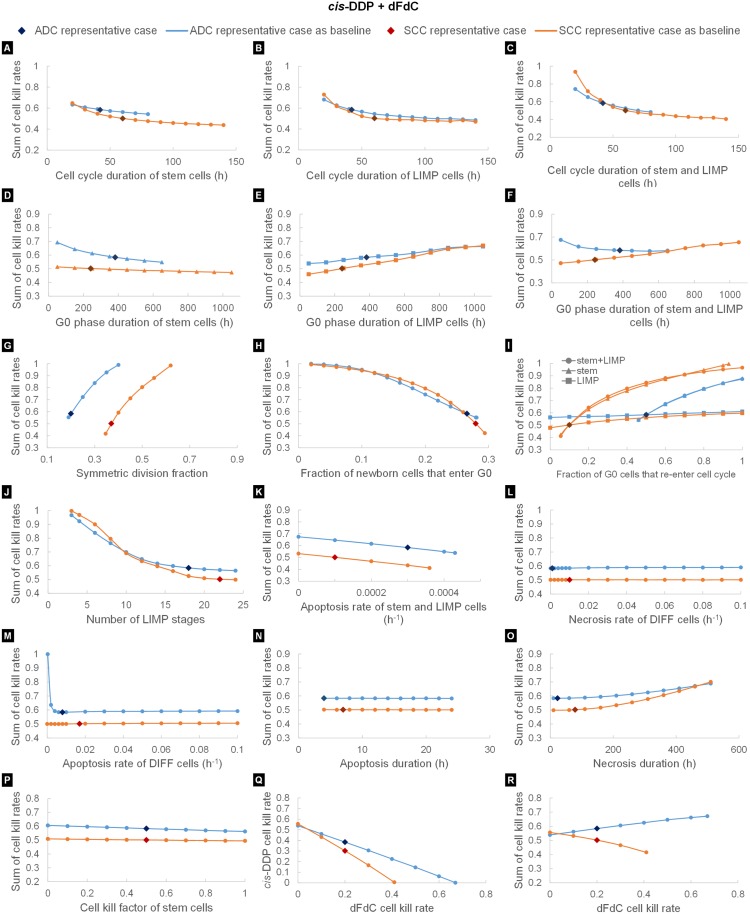
Results of the One-Factor-At-a-Time (OFAT) studies. The effect of model input parameters on the estimation of sum of cisplatin and gemcitabine cell kill rates is studied by varying one input parameter at a time, while keeping the others at a baseline value. Two sets of baseline values have been considered (Table 5), corresponding to a SCC and an ADC representative case. The model parameters investigated are: (A) duration of cell cycle of stem cells, (B) duration of cell cycle of LIMP cells, (C) duration of cell cycle, when considered equal for both stem and LIMP cells, (D) residence time of stem cells in G0 phase, (E) residence time of LIMP cells in G0 phase, (F) residence time of cells in a G0 phase, when considered equal for both stem and LIMP cells, (G) fraction of stem cells that undergo symmetric division, (H) fraction of newborn cells that enter a quiescent state following mitosis, (I) fraction of stem cells that re-enter cell cycle from a quiescent state, fraction of LIMP cells that re-enter cell cycle from a quiescent state, fraction of cells that re-enter cell cycle from a quiescent state, the latter considered equal for both stem and LIMP cells, (J) number of mitoses performed by LIMP cells before becoming terminally differentiated, (K) number of stem and LIMP cells that enter the apoptotic pathway per hour, (L) number of DIFF cells that enter the necrotic pathway per hour, (M) number of DIFF cells that enter the apoptotic pathway per hour, (N) time between the onset of apoptosis and the removal of the apoptotic bodies, (O) time between the onset of necrosis and the removal of its products, (P) resistance of stem cells to chemotherapy, expressed as the ratio of the stem cell kill rate to the estimated drug cell kill rate and (R) assumed cell kill rate of gemcitabine. In (Q) the effect of the assumed cell kill rate of gemcitabine on the estimation of cisplatin’s cell kill rate is depicted. Abbreviations: ADC: Adenocarcinoma, SCC: Squamous cell carcinoma, cis-DDP: cisplatin, dFdC: gemcitabine, LIMP: LImited Mitotic Potential tumor cell (also called committed or restricted progenitor cell), DIFF: terminally DIFFerentiated tumor cell. G0: dormant, resting phase.

As the cell cycle time of stem cells, *T*_*C*,*stem*_, increases, the estimated CKR decreases (Fig 3A). The magnitude of the effect is pronounced for short cell cycle times and depends on the values assigned to the rest of the models parameters. *T*_*C*,*stem*_ is related to the tumor growth rate [12]. Long cell cycle times indicate a slow population regrowth following chemotherapy administrations and, hence, require lower CKRs to achieve the target reduction in tumor size. It is noted that for the ADC representative case, values of *T*_*C*,*stem*_ above 80h are biological non-relevant as they result in a negative growth rate, namely, the tumor shrinks over time by itself. Similarly, when the cell cycle time of LIMP cells *T*_*C*,*LIMP*_, increases the estimated CKR decreases (Fig 3B). However, the parameter has no effect on the tumor growth rate (S1 Fig). Instead, increasing *T*_*C*,*LIMP*_ results in a notable increase in the GF (S2 Fig). As chemotherapy directly targets the cancer cells with proliferative capacity, a higher GF implies a higher amount of treatment-related cell losses. Therefore, a lower CKR is needed to achieve the target tumor shrinkage. For prolonged durations, the effect of *T*_*C*,*LIMP*_ is negligible. The combined effect when varying simultaneously the cell cycle time of both stem and LIMP cells is depicted in Fig 3C. Due to the analogous effect of *T*_*C*,*stem*_ and *T*_*C*,*LIMP*_ a similar overall pattern is observed of increased, however, magnitude for short cell cycle times.

The increase of dormant phase duration of stem cells, *T*_*G0*,*stem*_, leads to a decrease in the estimated CKR (Fig 3D), since higher values of the parameter are associated with lower growth rates. The effect is significant for the value combination of the ADC representative case; however, it is negligible for the SCC case. It is noted that for the ADC representative case, values of *T*_*G0*,*stem*_ above 650h result in negative growth rates. In contrast, increase of the residence time of LIMP cells in dormant phase, *T*_*G0*,*LIMP*_, leads to an increase in the estimated CKR (Fig 3E). The parameter affects the population composition of the tumor. Long-lived dormant cells indicate a higher proportion of G0 cells in tumor bulk at the expense of other populations including actively proliferating cells. Moreover, in our modelling approach we assume that G0 cells lethally hit by cisplatin need to re-enter cell cycle in order to die. However, for prolonged durations, few lethally hit G0 cells will eventually be removed during the simulation time. When both *T*_*G0*,*stem*_ and *T*_*G0*,*LIMP*_ are varied simultaneously, the effect of *T*_*G0*,*stem*_ prevails for the ADC representative case and the effect of *T*_*G0*,*LIMP*_ prevails for the SCC case (Fig 3F).

We observe a considerable increase in the estimated CKR when increasing the symmetric division fraction of stem cells, *P*_*sym*_ (Fig 3G). The parameter primarily regulates the rate of tumor growth [12], with high values indicating fast growing tumors and rapid repopulation of tumor cells during therapy. Therefore, a higher cytotoxic efficacy of the drug(s) is required to achieve the target reduction in tumor size. It is noted that low values of the parameter result in negative growth rates, whereas for high values the repopulation is so rapid that chemotherapy cannot achieve the target tumor shrinkage even when the sum of CKRs is 1. It is worth noting that a sum of CKRs equal to 1 does not imply that all cells with proliferative potential will die, since stem cells are assumed chemo-resistant and only a fraction determined by the product CKF*CKR will be lethally hit. Moreover, dormant cells are assumed to be affected only by cisplatin and not by gemcitabine.

An increase in the fraction of newborn cells that enter a quiescent state following mitosis, *P*_*sleep*_, leads to a considerable decrease in the estimated CKR (Fig 3H). A high *P*_*sleep*_ is associated with slow cancer cell repopulation in between chemotherapy intervals, due to the small number of newborn cells that begin a new cycle. Hence, a lower CKR is required to reduce tumor size. In contrast to parameter *P*_*sym*_, high values of the parameter result in negative growth rates, whereas for very low values, below 0.02, the target tumor shrinkage cannot be achieve due to the rapid tumor regrowth.

High values of the fraction of stem G0 cells that re-enter cell cycle, *P*_*G0toG1*,*stem*_, result in faster tumor repopulation in between chemotherapy intervals, as the number of dividing stem cells becomes higher. Hence, the estimated *CKR*_*sum*_ increases (Fig 3I). A less profound increase in the *CKR*_*sum*_, with the increase of the fraction of LIMP G0 cells that re-enter cell cycle, *P*_*G0toG1*,*LIMP*_, is observed (Fig 3I). This increase is insignificant in the case of ADC. The parameter does not affect the rate of cancer cell repopulation, but rather, it has a moderate effect on the proportion of cells with proliferative capacity. Higher values of *P*_*G0toG1*,*LIMP*_ indicate fewer cancer cells with proliferating capacity, therefore fewer cells will die following chemotherapy. When both *P*_*G0toG1*,*stem*_ and *P*_*G0toG1*,*LIMP*_ are varied simultaneously, the effect of *P*_*G0toG1*,*stem*_ prevails (Fig 3I).

The number of mitoses between stem cells and terminally differentiated cells, *N*_*LIMP*_, regulates the relative populations of stem and LIMP cells [12]. The decrease of *N*_*LIMP*_ leads to an increase in the percentage of stem cells. When therapy is considered to equally affect stem and LIMP cells, the parameter has no effect on CKR estimation (S3 Fig). However, when stem cells are assumed more resistant than LIMP cells, low values of *N*_*LIMP*_ require higher CKRs to achieve the target reduction in tumor size (Fig 3J). For very low numbers of mitoses, e.g. 1 or 2 (corresponding to more than 22% and 40% stem cells out of living cells for ADC and SCC representative cases, respectively), the target treatment-induced reduction in tumor size cannot be achieved for the value of CSC resistance considered.

#### Cell loss

Apoptosis rate, *R*_*A*_, has been considered the same for both stem and LIMP cells. As *R*_*A*_ increases, a decrease in the estimated CKR is observed (Fig 3K). By increasing *R*_*A*_, fewer cells reach mitosis and divide, thereby resulting in a slower tumor regrowth. There exists an upper limit on the value of *R*_*A*_, above which tumor growth cannot be sustained.

For the parameter value combinations of both ADC and SCC representative cases, the CKR estimate is not sensitive to the necrosis rate of terminally differentiated cells, *R*_*NDiff*_ (Fig 3L). Similarly, the effect of spontaneous apoptosis rate of terminally differentiated cells on CKR estimation is negligible for the SCC case and the corresponding value combination of model parameters (Fig 3M). However, a different behavior is observed for the ADC case. For values of *R*_*ADiff*_ close to zero, increase in the parameter value results in a sharp drop in the estimated CKR, whereas, for higher values, variation of the parameter has no effect. Low values of *R*_*ADiff*_ are associated with long-lived differentiated cells, thereby resulting in a high proportion of the population in the tumor bulk. Notably, when *R*_*ADiff*_ is close to 0, the proportion of differentiated cells can be as high as 80%. In the present study, chemotherapy is assumed to act by directly killing only tumor cells with proliferative potential. When the lifespan of the differentiated cell category is long, the effect of therapy on differentiated cells appears with considerable delay and their population declines at a lower extend during the simulation period. On the other hand, when the lifespan is small enough compared to the observation period, the effect of the above parameter on therapeutic outcome is negligible. The total absence of output variation for values of *R*_*ADiff*_ close to 0 in the case of SCC is caused by the high baseline value of *R*_*NDiff*_ (= 0.01h^-1^). Therefore, even when *R*_*ADiff*_ becomes 0, the overall elimination rate of differentiated cells remains high (corresponds to an overall lifespan of 100h).

Apoptosis duration seems to have no effect on the estimation of the CKRs of the drugs (Fig 3N), due to the rapid nature of the mechanism, and, consequently, the very low proportion of the apoptotic cells/debris in the tumor.

Increase in necrosis duration results in an increase in the estimated sum of CKRs (Fig 3O). However, the effect is significant only for prolonged durations of the order of several days, which are associated with slow elimination rates and high proportions of necrotic cells. On the contrary, for tumors characterized by relatively short necrosis duration and, consequently, low proportion of necrotic cells, *T*_*N*_ has a trivial effect in the estimation of CKRs.

#### Chemo-resistance of stem cells

Due to the very low frequency of stem cells considered in the present study, the resistance of stem cells seems to have a negligible effect, at least, on the short term treatment-induced reduction in tumor size and, subsequently, the CKR estimation, for both ADC and SCC representative cases (Fig 3P).

#### Cell killing efficacy of dFdC

We studied the effect of the CKR of gemcitabine, *CKR*_*dFdC*_, on both the estimated CKR of cisplatin, *CKR*_*cis-DDP*_ (Fig 3Q) and the sum of cell kill rates, *CKR*_*sum*_ (Fig 3R). As anticipated, an increase in the cytotoxic efficacy of gemcitabine results in a decrease in the *CKR*_*cis-DDP*_. We can observe that the plot intercepts the axis of *CKR*_*dFdC*_ at different points for ADC and SCC representative cases (Fig 3Q). In our modelling approach gemcitabine can cause lethal damage only to actively cycling cells and not to dormant cells. When only gemcitabine is administered (*CKR*_*cis-DDP*_ = 0), a higher CKR is required when GF is low, i.e. for the ADC representative case. Interestingly, the *CKR*_*sum*_ increases with the increase of *CKR*_*dFdC*_ for the ADC case, whereas the opposite behavior is observed for the SCC case.

#### OFAT sensitivity measures

**Fig 4:**
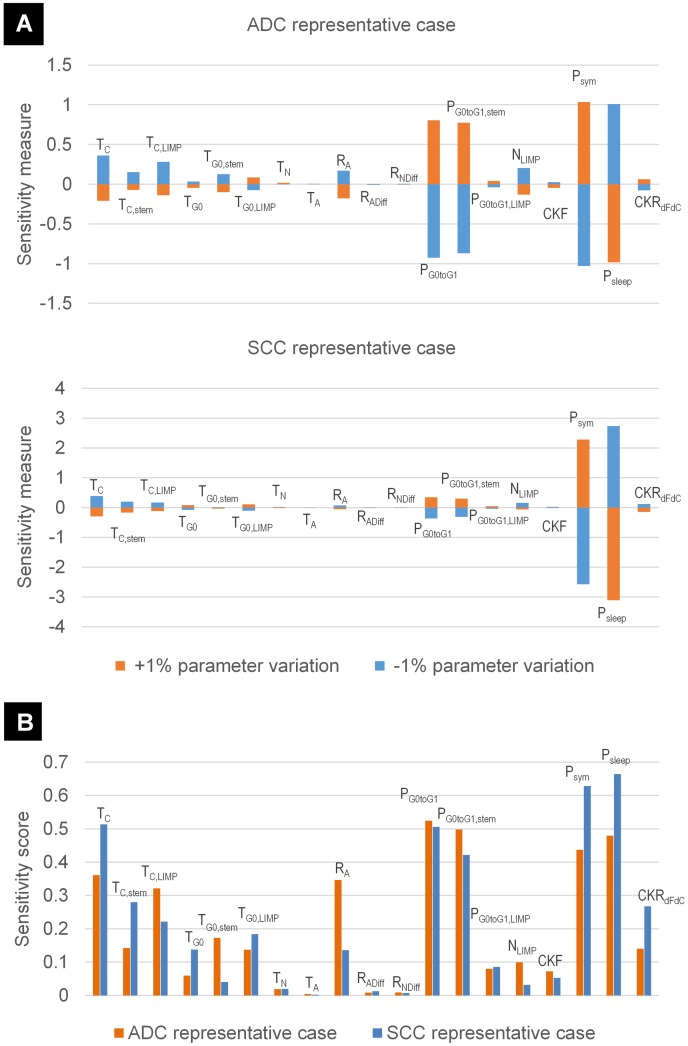
Results of the local sensitivity analysis. Each input parameter has been varied by ±10%, with the exception of *P*_*sym*_ and *P*_*sleep*_ that have been varied by ±5%. Following, the corresponding percentage change in the estimated sum of cisplatin and gemcitabine cell kill rates is recorded. The rest of the model parameters are kept at their baseline value (Table 5). Two sets of baseline values have been considered (Table 5), corresponding to a Squamous Cell Carcinoma (SCC) and an Adenocarcinoma (ADC) representative case. (A) Sensitivity measures for each input parameter defined as the % change in estimated sum of cell kill rates per +1% or -1% change in the input parameter, for ADC and SCC cases respectively. (B) Overall sensitivity score for each input parameter defined as the average of the sensitivity measures for ADC and SCC cases respectively, weighted by a normalized measure of input variability (the latter being the value range divided by the mean) (see Eq (3)). The value ranges considered are reported in S4 Text. Parameter symbols are explained in Table 4.

In addition to the qualitative study based on the OFAT plots, a quantitative sorting of the sensitivity of the model parameters is attempted in this paragraph. The results of the local sensitivity analysis, examining one parameter at a time, are presented in Fig 4. The percentage change in the estimated sum of CKRs, resulting from a ±1% change in each of the model parameters around its baseline value for the ADC and SCC representative cases are depicted in Fig 4A. We observe that the percentage change in the output is asymmetric for changes above and below the baseline value of the parameters. The local sensitivity highly depends on both the baseline value of the parameter under investigation and the value combination of the remaining model parameters. However, for all cases considered, the analysis shows that CKR estimation is most sensitive to the fraction of stem cells that perform symmetric divisions, *P*_*sym*_, and the fraction of newborn cells that withdraw from the active cycle to a dormant phase, *P*_*sleep*_. Variation in the aforementioned parameters may cause substantial changes in the estimated *CKR*_*sum*_ by a factor of 1 to 3, with the SCC representative case exhibiting a higher sensitivity. The remaining model parameters cause a variation in the *CKR*_*sum*_ that is always lower than the percentage variation of the input. More specifically, the model is relatively more sensitive to the fraction of stem G0 cells that re-enter cell cycle, *P*_*G0toG1*,*stem*_, with sensitivities ~0.8% and ~0.3% for the ADC and SCC cases, respectively. The 1% variation of the cell cycle time of stem, *T*_*C*,*stem*_, or LIMP, *T*_*C*,*LIMP*_, cells causes a moderate variation in the estimated *CKR*_*sum*_, in the range ~0.1%—~0.2%. The effect is more prevailing when both parameters are varied at once. For the ADC case the estimation of *CKR*_*sum*_ is more sensitive to *T*_*C*,*LIMP*_, whereas quite the opposite stands for the SCC case. The estimation of *CKR*_*sum*_ is fairly sensitive to the number of mitoses between stem and terminally differentiated cells, *N*_*LIMP*_, with sensitivities not exceeding ~0.17%. A low sensitivity is observed for the apoptosis rate of stem and LIMP cells, *R*_*A*_, approximately 0.17% and 0.07% for the ADC and the SCC cases, respectively. The model also exhibits a low sensitivity to the considered cytotoxicity of the second drug, *CKR*_*dFdC*_, with sensitivities of ~0.07% and ~0.13% per 1% variation of the parameter. The lower sensitivity is observed for ADC case. The model is relatively insensitive to the residence time of stem, *T*_*G0*,*stem*,_ or LIMP cells, *T*_*G0*,*LIMP*,_ in dormant phase with sensitivities of the order of ~0.1%. The model seems rather robust to the remaining model parameters, for both ADC and SCC representative cases, with sensitivities in the range ~0.003% to ~0.04% per 1% percentage change in the input.

A range sensitivity analysis has been realized (S4 Text) with the aim to evaluate the effect of the non-linear relationships between the model parameters and the estimated sum of CKRs on sensitivity. The method is a variation of the aforementioned local sensitivity measures. The parameters are now allowed to vary over the anticipated value range, instead of a small perturbation around their baseline values. Despite the non-linearity, the results of the local and the range methods are pretty consistent (S4 Text).

The consideration of parameters variability is expected to be an improvement over the local sensitivity measures. For example, a parameter with a high local sensitivity, but a narrow value range, may eventually be less influential than initially indicated. Fig 4B presents the overall sensitivity score for each parameter after taking into account parameters variability, for ADC and SCC representative cases respectively. The sensitivity score shows, as before, the *P*_*sleep*_, *P*_*sym*_, *P*_*G0toG1*,*stem*_ and *T*_*C*,*LIMP*_ to be the most influential parameters (with descending order). The analysis yields a moderate sensitivity score for *R*_*A*_, indicating a higher rank than before. A relatively low sensitivity is still indicated for *T*_*C*,*stem*_, *CKR*_*dFdC*_ and *T*_*G0*,*LIMP*_, whereas the model is rather insensitive to *T*_*G0*,*stem*_, *P*_*G0toG1*,*LIMP*_, *N*_*LIMP*_ and *CKF*. It should be noted that the sensitivity score ranks the parameter *N*_*LIMP*_ considerably lower than the sensitivity measure. Finally, the parameters *T*_*N*_, *R*_*ADiff*_, *R*_*NDiff*_ and *T*_*A*_ seem to have no effect on the estimation of the sum of CKRs, as before.

### Effect of cellular proliferation kinetics on CKR evaluation: LHS/PRCC analysis results

The LHS/PRCC method has been chosen to investigate the effect of the model input parameters as well as of selected macroscopical proliferation features of the tumor, i.e. volume growth rate and tumor cell population constitution, on the estimation precision of *CKR*_*sum*_, by taking into account the presence of correlations between the inputs.

#### Model parameters value ranges

The definition of appropriate value ranges for the input parameters is determinant for the results of the present analysis. Table 4 lists the value ranges considered. In our modelling approach hypoxic cells are assumed to be in a G0 state. The duration of G0 phase reflects the ‘average’ lifetime of cells found in G0 either due to hypoxia or due to lack of growth signals. We assume that shortly lived G0 cells are primarily associated with hypoxia. Based on literature, we consider approximately four days [29] to be the lower limit of *T*_*G0*_, corresponding to a tumor with a high hypoxic fraction of G0 cells. However, *T*_*G0*_ of few days is not consistent with tumors characterized by a low GF and a high fraction of quiescent cells and thus relatively prolonged *T*_*G0*_ should be considered. For study purposes we take 50 days to be the upper extreme value for *T*_*G0*_ for both stem and LIMP cells.

The ability of CD133^+^ lung cancer cells, to reconstitute the parental population within only a few doublings, indicates that asymmetric CSC divisions are favored over symmetric self-renewals [30]. Moreover, in [31] the CSC symmetric division frequency in sphere cultures of murine lung cancer cell lines was estimated below 0.2 (per day). The value range of *P*_*sym*_ and *N*_*LIMP*_ has been defined based on the above observations as well as the constraint for a rare CSC population (Table F in S3 Text). Assuming that the fraction of CSC in NSCLC cannot exceed 0.001 and that stem cells tend to divide asymmetrically, we have varied *P*_*sym*_ between 0 and 0.4 and *N*_*LIMP*_ between 8 and 24. When *N*_*LIMP*_ is lower than 8, the upper boundary for CSC fraction is exceeded for all of the parameter value combinations tested (S4 Fig). Furthermore, high values of *P*_*sym*_, besides implying a tendency towards symmetric self-renewals, are associated with high fractions of stem cells, and would require notably higher numbers of mitoses between stem cells and terminally differentiated cells, *N*_*LIMP*_, to be regarded.

Apoptosis, from initiation to phagocytic removal of apoptotic bodies, is a rapid process completed within hours. Here, we have considered that apoptosis time, *T*_*A*_, ranges between 1 and 25 hours [32–34]. In contrast, the elimination of necrosis products is a time consuming process [35]. A value range of necrosis time, *T*_*N*_, between 1 and 200 hours ensures that the percent of necrosis, despite its considerably wide range, is kept below 30% in the majority of virtual tumor implementations, in line with literature observations (Table E in S3 Text).

Taking into consideration that cancer cells have developed mechanisms to escape apoptosis, the rate of spontaneous apoptosis of stem and LIMP cells, *R*_*A*_, has been allowed to vary between 0 and 0.001 per hour. It is noted that apoptosis rate of stem cells is one of the model parameters that regulate tumor growth rate [12]. For a given set of parameter values, an upper limit exists, that correspond to a balance between proliferation and apoptosis, above which tumor growth cannot longer be sustained and growth rate becomes negative [12]. For the value range of *R*_*A*_ considered, value combinations of the rest of the model parameters that correspond to positive growth rates always exist for all values of *R*_*A*_ considered (see S5 Text).

Most normal epithelial cells of the lung are believed to have a lifespan of about four months; however, estimates span from 8 days to more than 17 months, depending on cell type and location [36–39]. The apoptosis rate of NSCLC terminally differentiated cells, *R*_*ADiff*_, corresponds to the reciprocal of their lifespan. Due to the lack of relevant data, as a first approximation, we allow *R*_*ADiff*_ to take values between 0.0001 h^-1^–0.02h^-1^, a value range that also includes the lifespans of the normal bronchial epithelium. Finally, for study purposes, we have allowed the rate at which terminally differentiated cells die due to inadequate oxygenation through necrosis, *R*_*NDiff*_, to vary between 0 and 0.02 per hour. It is noted that based on the results of the OFAT analysis (see below), the model is not sensitive to this parameter for values greater than the upper limit considered.

The parameter *CKR*_*dFdC*_ has been assumed to be equal to 0.2.

#### LHS scatterplots

**Fig 5:**
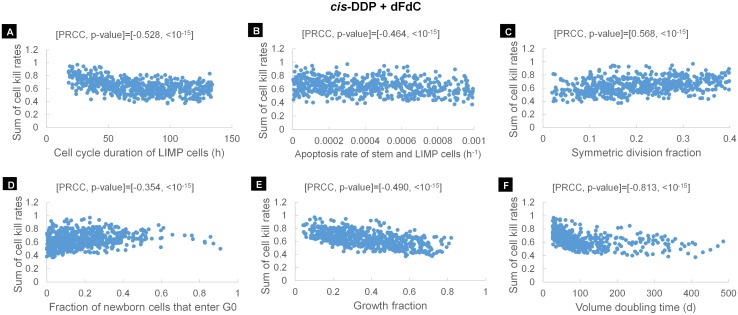
Partial Rank Correlation Coefficient (PRCC) scatterplots of indicative model parameters and tumor proliferation features. All model parameters are varied simultaneously. The ordinate represents the sum of cisplatin and gemcitabine cell kill rates. The sample has a size of N = 553. It originates from a Latin Hypercube Sampling run of 8000 combinations of parameter values after excluding the ones with negative growth rates, T_d_ below 26 days and stem cell fractions higher than 1‰. The PRCC value and the corresponding p-value are displayed in each plot. The scatterplots are displayed for the following model parameters and proliferation features: (A) duration of cell cycle of LIMP cells, (B) number of stem and LIMP cells that enter the apoptotic pathway per hour, (C) fraction of stem cells that undergo symmetric division, (D) fraction of newborn cells that enter a quiescent state following mitosis, (E) proportion of living tumor cells that are actively proliferating and (F) doubling time of tumor volume. Abbreviations: cis-DDP: cisplatin, dFdC: gemcitabine, LIMP: LImited Mitotic Potential tumor cell (also called committed or restricted progenitor cell), G0: dormant, resting phase.

LHS has been run to produce *8000* combinations of parameter values. After excluding combinations that result in biologically non relevant tumors, i.e. negative growth rates, or in tumors with NSCLC non relevant proliferation dynamics, i.e. T_d_ below 26 days and/or stem cell fractions higher than 1‰ (based on SoA results), a subset of 554 combinations has been finally considered for the subsequent analysis. For each of the 554 sets the *CKR*_*cis-DDP*_, and therefore *CKR*_*sum*_, that results in the targeted volume reduction is estimated. Furthermore, for each combination, the T_d_ and the distribution of the total population in the various cell types of the virtual tumor (denoted as tumor proliferation features) are recorded. Scatter plots of *CKR*_*sum*_ against the 554 values of each input parameter and tumor proliferation feature are then produced. Fig 5 displays the scatter plots for selected input parameters and cell proliferation features. Based on a visual inspection of these scatter plots, the variables that seem to have the strongest correlation with *CKR*_*sum*_ are *T*_*C*,*LIMP*_, *P*_*sym*_, *P*_*sleep*_, *T*_*d*_, GF and G0/living.

#### Partial rank correlation coefficients

PRCC analysis is suitable only when the outcome measure is monotonically related to the input parameters. The OFAT plots (Fig 3) verify the monotonic, and nonlinear, relationship between the *CKR*_*sum*_ and the model parameters and justify the eligibility of the method. The analysis attempts to assess the correlation between each input parameter and the output measure (*CKR*_*sum*_), while removing the effect of the remaining input parameters. The calculated PRCC values between each input model parameter and the estimated *CKR*_*sum*_ are given in Table 6. Values of PRCC close to ±1 and low p-values suggest a strong influence of the input parameter on output, whereas PRCC values close to 0 or high p-values suggest no correlation. The negative sign indicates an inverse proportionality between the input parameter and the outcome measure. For the parameter ranges considered, the analysis shows that parameters *P*_*sym*_, *T*_*C*,*LIMP*_ and *R*_*A*_ have the highest impact (with descending order) on the estimation of *CKR*_*sum*_, with a correlation coefficient that varies between 0.57 and 0.46 (absolute value). A less strong correlation, with coefficients approximately between 0.38 and 0.35, is observed in the cases of (with descending order) *P*_*G0toG1*_ of stem cells, *T*_*C*_ of stem cells, *P*_*sleep*_ and *T*_*G0*_ of LIMP cells. For the parameters *T*_*G0*_ of stem cells, *CKF* of stem cells and *N*_*LIMP*_, the coefficients do not exceed 0.25 (absolute value) indicating a relatively week correlation. The analysis did not reveal any correlation for the remaining input parameters.

**Table 6:** PRCC results.

1^st^ analysis			2^nd^ analysis		
	PRCC	p-value		PRCC	p-value
**T**_**C,stem**_	-0.362	4.30*10^−18*5*^	**SF**	0.126	3.14*10^−3^
**T**_**C,LIMP**_	-0.528	4.67*10^−40^	**LF**	0.129	2.56*10^−3^
**T**_**G0,stem**_	-0.245	8.53*10^−9^	**GF**	-0.490	2.33*10^−34^
**T**_**G0,LIMP**_	0.346	1.31*10^−16^	**QF**	-0.0859	0.0446
**T**_**N**_	0.0606	0.164	**AF**	0.0182	0.672
**T**_**A**_	-0.0212	0.613	**NF**	0.120	4.89*10^−3^
**R**_**A**_	-0.464	3.92*10^−30^	**T**_**d**_	-0.813	2.16*10^−130^
**R**_**ADiff**_	-0.064	0.138			
**R**_**NDiff**_	-0.0244	0.584			
**P**_**G0toG1,stem**_	0.381	4.14*10^−20^			
**P**_**G0toG1,LIMP**_	0.0633	0.142			
**P**_**sym**_	0.568	2.63*10^−47^			
**P**_**sleep**_	-0.354	2.46*10^−17^			
**N**_**LIMP**_	-0.194	5.47*10^−6^			
**CKF**	-0.205	1.55*10^*−6*^			

Values rounded to three significant figures

A second PRCC study has also been performed attempting to assess the correlation between various tumor proliferation features and the output measure, while controlling for the effect of the rest of the proliferation features considered. The proliferation features under investigation are the *T*_*d*_, the fraction of stem cells (SF) out of living cells, the fraction of LIMP cells (LF) out of living cells, the GF, the fraction of quiescent G0 cells (QF) out of living cells, the fraction of necrotic cells (NF) out of total cells and the fraction of apoptotic cells (AF) out of total cells. They all refer to the time at diagnosis prior treatment onset. They do not constitute input model parameters but are modulated based on the values attributed to the model parameters. The scatterplots of Fig 5 indicate a monotonic relationship between the above features and the *CKR*_*sum*_. Based on Table 6 the most prominent cell proliferation feature for the estimation of *CKR*_*sum*_ is the *T*_*d*_, with a PRCC value of -0.8. A strong correlation is also observed in the case of GF (PRCC value: -0.49). For SF, LF and NF, a week influence on CKR estimation is indicated, whereas no correlation is observed for QF and AF.

Additionally, we have examined the sensitivity of the ratios of stem cells to LIMP cells (SLF) and stem cells to DIFF cells (SDF). These ratios are strongly correlated with the SF. Since the PRCC method may underestimate the sensitivity in the case of inputs with very high correlations among them [40], two additional PRCC studies have been performed similar to the second one, but with the ratios SLF and SDF considered in the place of SF, respectively (S1 Table). A weak correlation with the estimated CKR is indicated, for both cases, and of the same magnitude as in the case of SF.

### CKR estimates

The histology, treatment and response data of 13 lung cancer clinical cases have been retrospectively analyzed in order to evaluate the *in vivo* efficacy of three different cisplatin-based doublet regimens: cisplatin plus gemcitabine, ciplatin plus docetaxel and ciplatin plus vinorelbine. The tumor proliferation features used for the OFAT sensitivity analysis have been also considered here. More specifically, for the clinical cases classified as ADC the assumptions are as follows: (a) T_d_ = 225d, (b) GF = 0.18, (c) AF = 0.01, (d) NF = 0.02 and (e) SF<0.001. For the clinical cases classified as SCC we have considered: (a) T_d_ = 109d, (b) GF = 0.36, (c) AF = 0.01, (d) NF = 0.20 and (e) SF<0.001. Furthermore, the ranges of the model parameters are the ones considered for the LHS/PRCC analysis, identical for both ADC and SCC subtypes, and are given in Table 4. LHS has been run to generate *2000* combinations of parameter values that fulfill the above requirements, as described in Material and Methods. After excluding combinations that result in biologically non-relevant tumors, i.e. negative cell class transition rates *P*_*sleep*_ and *R*_*NDiff*_, or in tumors with non-relevant proliferation dynamics, i.e. T_d_ beyond ±10% of the above assumptions and/or stem cell fractions higher than 1‰ (based on SoA results) and/or *T*_*A*_*>*24h and/or *T*_*N*_>200h and/or *R*_*NDiff*_>0.02h^-1^, a subset of N = 146 and 175 combinations has been finally considered for the SCC and ADC cases respectively.

**Fig 6:**
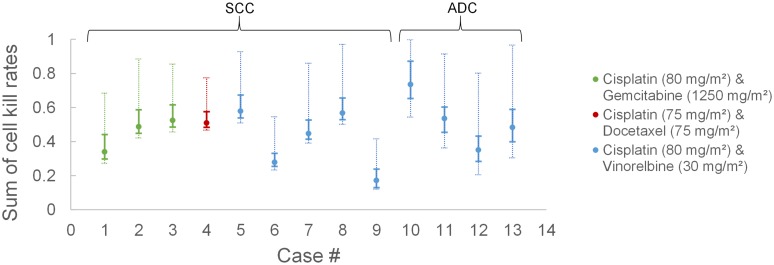
Drug cytotoxicity results for the clinical cases. The estimated sum of cell kill rates, *CKR*_*sum*_ for the cisplatin-based doublet regimen given to each clinical case (denoted by its ID number) is displayed. Latin Hypercube Sampling has been run to produce two sets of value combinations of model parameters, one for the Adenocarcinoma (ADC) and one for the Squamous Cell Carcinoma (SCC) clinical cases. For each value combination the *CKR*_*sum*_ that results in the clinically observed volume reduction is determined. At any given case the dot denotes the median (50^th^ percentile) of the N estimated values, the lower and upper boundaries of the solid line denote the 10^th^ and 90^th^ percentile, whereas the dashed line extends to the two extreme values of the estimated *CKR*_*sum*_.

For each clinical case and for each of the N sets, the *CKR*_*cis-DDP*_, and therefore *CKR*_*sum*_, that results in the observed volume reduction is estimated. The cell kill rate of the second drug, CKR_B_, has been assumed equal to 0.2 with the exception of clinical case 9 for which a value equal to 0.1 has been assumed. This selection of CKR_B_ value has been made with the criterion to have a solution for *CKR*_*cis-DDP*_ for all the N LHS combinations considered. Fig 6 shows the median (dot), 10^th^ and 90^th^ percentile (lower and upper boundary of solid line) and minimum and maximum (lower and upper boundary of dashed line) of the N *CKR*_*sum*_ estimates for each clinical case. Even though the estimated values of *CKR*_*sum*_ vary considerably for all clinical cases, the vast majority of the values (80%) is confined in a much smaller range, not exceeding, with the exception of one case, an average of 0.1 units around the median value. We observe that the treatment efficacy is very low in 2 cases (15.4% of the total cases) with a median *CKR*_*sum*_ below 0.3, low in 2 cases (15.4% of the total cases) with a median *CKR*_*sum*_ approximately 0.38, moderate in 6 cases (46.1% of the total cases) with a median *CKR*_*sum*_ between 0.47 and 0.57, high in 2 cases (15.4% of the total cases) with a median *CKR*_*sum*_ approximately 0.61 and very high in 1 case (7.7% of the total cases) with a median *CKR*_*sum*_ approximately 0.71. The efficacy of the three cisplatin-doublet regimens seems comparable; however, the sample size is too small, especially for cisplatin/docetaxel and cisplatin/gemcitabine regimens, to draw statistically significant conclusions.

**Fig 7:**
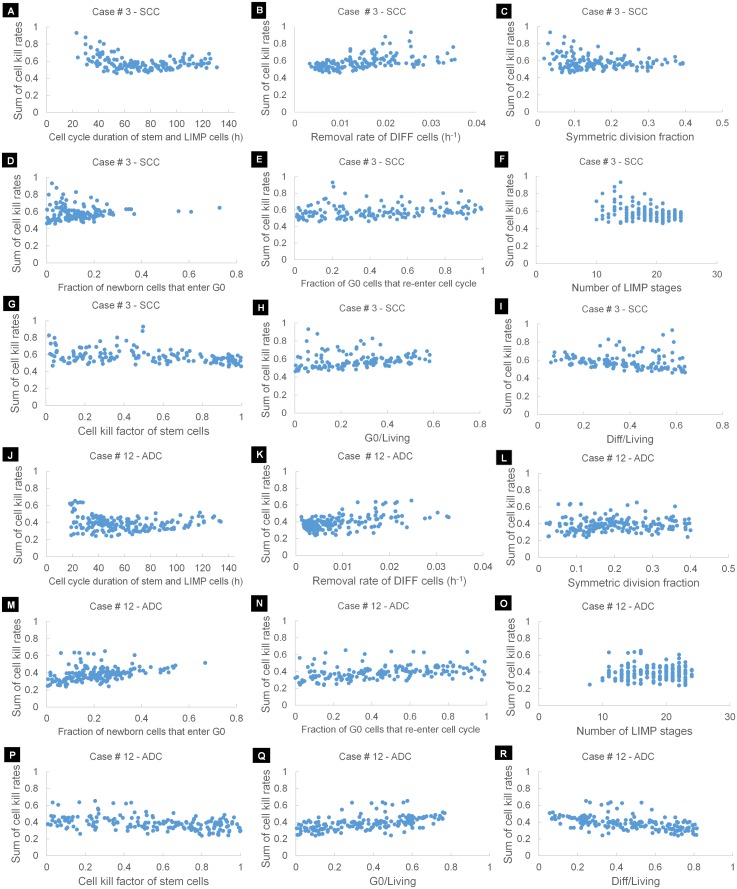
Scatterplots of the cell kill rate estimates vs. indicative model parameters and tumor proliferation features for the clinical cases 3 (Squamous Cell Carcinoma—SCC) and 12 (Adenocarcinoma—ADC). All model parameters are varied simultaneously. The ordinate represents the sum of cisplatin and gemcitabine cell kill rates for case #3 (panels (A)-(I)) and cisplatin and vinorelbine cell kill rates for case #12 (panels (J)-(R)). The samples have a size of 146 and 175 for the SCC (#3) and ADC (#12) cases, respectively. They originate from two Latin Hypercube Sampling runs of 2000 combinations of parameter values after excluding the ones with negative cell proliferation kinetics or cell proliferation kinetics that fall beyond the reference values for non-small cell lung cancer in general and SCC and ADC representative cases specifically (see Results section). The scatterplots are displayed for the following model parameters and proliferation features: (A) and (J) duration of cell cycle, when considered equal for both stem and LIMP cells, (B) and (K) removal rate of DIFF cells defined as the number of cells that enter the apoptotic or necrotic pathway per hour, (C) and (L) fraction of stem cells that undergo symmetric division, (D) and (M) fraction of newborn cells that enter a quiescent state following mitosis, (E) and (N) fraction of cells that re-enter cell cycle from a quiescent state, when considered equal for both stem and LIMP cells, (F) and (O) number of mitoses performed by LIMP cells before becoming terminally differentiated, (G) and (P) resistance of stem cells to chemotherapy, expressed as the ratio of the stem cell kill rate to the estimated drug cell kill rate, (H) and (Q) the proportion of living tumor cells that are in a quiescent state, (I) and (R) the proportion of living tumor cells that are terminally differentiated. Abbreviations: ADC: Adenocarcinoma, SCC: Squamous cell carcinoma, LIMP: LImited Mitotic Potential tumor cell (also called committed or restricted progenitor cell), DIFF: terminally DIFFerentiated tumor cell. G0: dormant, resting phase.

In Fig 7 the scatterplots of the N *CKR*_*sum*_ values for one SCC and one ADC clinical case are shown versus indicative model parameters and the proportion of dormant and terminally differentiated cells. We observe that for both ADC and SCC cases, the highest values of *CKR*_*sum*_ are associated with short cell cycle durations (Fig 7A and 7J), shortly lived terminal differentiated cells (or equivalent removal rate of DIFF cells above 0.01h^-1^) (Fig 7B and 7K) and resistant stem cells by a factor of 2 or above (or equivalent CKF of stem cells<0.5) (Fig 7G and 7P). For the SCC case the highest values of *CKR*_*sum*_ are found for low P_sym_ (Fig 7C) and low P_sleep_ (Fig 7D). Furthermore, tumors with higher fractions of dormant cells, at the expense of terminally differentiated cells (GF is constant), tend to require a higher *CKR*_*sum*_ to achieve the same volume reduction (Fig 7H, 7I, 7Q and 7R).

### Model validation

The three mutually perpendicular dimensions of the surgically resected tumor has been provided for the 12 out of the 13 patients (Table 7). An early validation of the proposed model/methodology is attempted here, by comparing the post-surgical excision measurements with the simulated tumor size at the time of surgery. It should be noted that the size of the resected tumor has not been considered for the estimation of *CKR*_*cis-DDP*_ in the previous section; however, it is exploited here for validation purposes. In particular, for each clinical case and for each of the N parameter sets with corresponding *CKR*_*cis-DDP*_ derived in the previous section, an extended simulation has been performed starting from the first CT acquisition until the date of surgery. The extra time interval simulated for each clinical case is reported in Table 7. For three clinical cases, additional drug administrations have taken place during this interval and have been modelled assuming the estimated values of *CKR*_*cis-DDP*_.

**Table 7:** Clinical data for model validation.

Case #	Interval between second CT and surgery (days)	Tumor maximum diameter a (cm)	Tumor maximum diameter b (cm)	Tumor maximum diameter c (cm)	Volume (ml)*	Equivalent tumor diameter (cm)*	Additional drug administrations (day)^‡^
**1**	20	6	4.5	3	42.41	4.33	none
**2**	21	6	5.5	4	69.12	5.09	GEM: 43^rd^, 50^th^, CIS: 43^rd^
**3**	18	2.5	1.6	1.6	3.35	1.86	none
**4**	36	3	2	1.5	4.71	2.08	DOC: 44^th^, CIS: 44^th^
**5**	5	2.5	1.8	1.5	3.53	1.89	none
**6**	39	4	3.7	3	23.25	3.54	VIN: 50^th^
**7**	7	3.5	3.3	2.5	15.12	3.07	none
**8**	3	3.5	3.5	3.3	21.17	3.43	none
**9**	28	8	7.5	5.5	172.79	6.91	none
**10**	20	2.1	1.9	1.3	2.72	1.73	none
**11**	6	4	3.5	2	14.66	3.04	none
**13**	22	4.7	4	3.1	30.52	3.88	none

*Values rounded to two decimal places.

^**‡**^ Day 1 is treatment onset. Only the drug administration time points in the interim between the second CT and the surgery are recorded.

**Fig 8:**
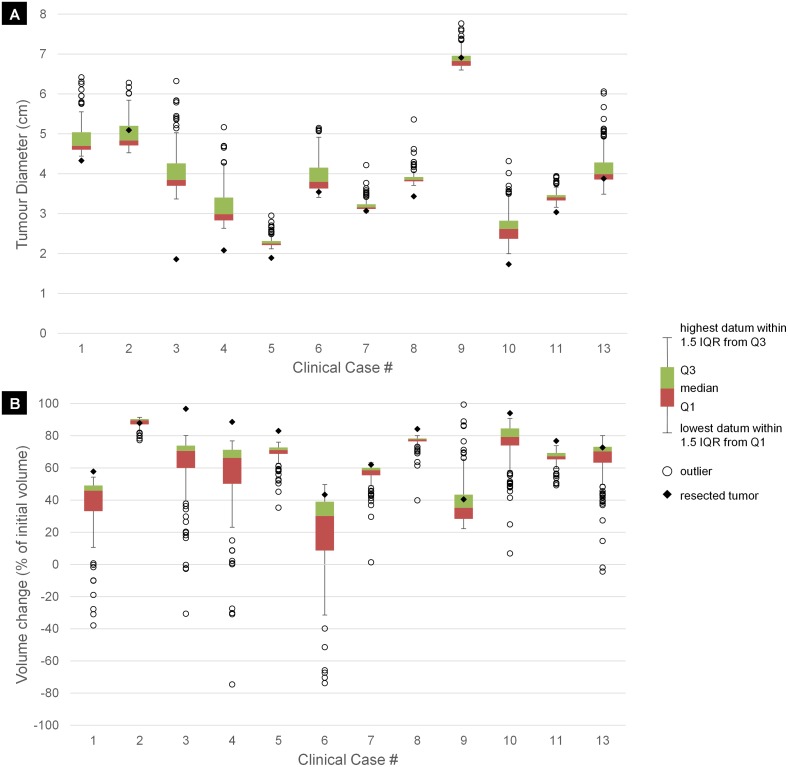
Box-and-whisker plots of model predictions at the time of surgery for the clinical cases. Each box and whisker plot corresponds to N (146 and 175 for the squamous cell carcinoma and the adenocarcinoma cases respectively) independent predictions. Each prediction results from an extended model simulation starting from the first CT examination till the time of surgery. The simulations have assumed the estimated sum of cell kill rates, *CKR*_*sum*_ of Fig 6. At any given case the horizontal line between the green and the red boxes denotes the median (50^th^ percentile) of the N predictions, the lower boundary of the red box and the upper boundary of the green box denote the first (Q1) and third (Q3) quartiles, whereas the predictions more than 1.5 interquartile (IQR) distance from the end of the boxes are denoted as outliers (depicted as circles). The whiskers extend from the lowest to the highest prediction that falls within 1.5 IQR from the outer edge of the boxes. The predictions correspond to (A) the equivalent diameter of the tumor at the time of surgery, defined as the diameter of a sphere with the same tumor volume as the predicted one and (B) the absolute value of the volume change of the tumor, expressed as a percentage of the initial volume. For the clinical case # 9 the volume change corresponds to an increase, whereas for rest cases to a reduction. The corresponding values derived by the measurements of the surgical resected tumor are denoted as a filled rhomb.

Fig 8A depicts the boxplots of the N predicted tumor sizes for each clinical case in comparison with the real ones. Since it is customary in clinical practice to assess the size of a solid tumor by measuring its maximum dimension(s), tumor size is expressed by means of an equivalent diameter. The predicted tumor volume, V, is translated to the equivalent diameter, d, assuming that the tumor is approximated by a sphere of the same volume:

$$d=2\times \sqrt[{3}]{\frac{3V}{4\pi }}$$ (16)

The equivalent diameter of the surgically resected tumor is calculated as before. The volume of the resected specimen is determined by its three mutually perpendicular dimensions, after assuming that its shape is approximated by a triaxial ellipsoid [41] (Table 7). In Fig 8B the predicted volume reduction is compared with the real one. Despite the large deviation observed between the minimum and the maximum values in both equivalent diameter and volume reduction graphs, the majority of the predictions are in fact confined around the median value. To that respect, the first, second (median) and third quartiles are considered for the validation of our predictions.

A good agreement between the prediction and the actual data is observed for the six out of twelve cases. In particular, the equivalent diameter of the resected tumor and, hence, the observed volume reduction, falls between the first and third quartiles of the predicted ones in three cases (# 2, 9, 13). Moreover, a deviation less than 10% (corresponding to less than 4mm difference) between the predicted median diameter and the one of the resected tumor is observed for three cases (# 1, 6 and 7). However, due to the small size of the initial tumor, a somewhat larger deviation is observed in the case of volume reduction for cases # 1 and 6. It is noteworthy that the cases # 2 and 6 received additional therapy during the extra simulation period.

A slightly higher deviation (12% to 20%) between the equivalent diameters of the median of the predicted values and the resected specimen is observed for the cases 5, 8 and 11. An even lower deviation that does not exceed 14% exists for volume reduction in the same cases. It is worth mentioning that for these cases the surgery takes place shortly after the second CT acquisition (Table 7), corresponding to a limited (after the second CT acquisition) simulated period; therefore, it is possible that any uncertainties in estimating the true tumor volume by both the imaging techniques and the post-surgical excision measurements may be comparable to the change in tumor sizes and play a role in the observed deviations. For example, volume estimation errors of 10 to 20% are typically expected (more prominent for small lesions) [15], whereas based on our simulation results the volumetric change in a period of only a few days is less than 10%.

The less agreement is observed for cases # 3, 4 and 10. The model seems to underestimate the cell killing efficacy of treatment and predicts a tumor at surgery that deviates from the resected tumor around 1cm for # 4 and #10 and 2cm for #3. However, for #10 the median volume reduction differs only 15% from the real one. The tumor proliferation profile considered may be a possible source of the observed discrepancies. Our literature study (S3 Text) has revealed a considerable intra-patient variability in the macroscopic characteristics of NSCLC. It is therefore expected our assumptions to deviate from reality for some of the cases. For more accurate results, the clinical data should contain information about the proliferation profile per patient as well.

By taking into account the existence of uncertainties in estimating the true tumor volume as described above, the limited number of data sets available for the estimation of CKR and the consideration of a single proliferation profile differentiated only on the grounds of the two histological subtypes of the available clinical cases, the gross agreement between the predicted tumor values and the prost-surgery measured ones indicate that the proposed methodology/ model has a clear clinical potential. Additional adaptation and validation work has been planned in order to further adapt and validate both the model and the methodology.

## Discussion

Cancer is a highly complex phenomenon. Prediction of treatment failure and selection of the optimal therapeutic strategy can be greatly potentiated by the use of mechanistic models that summarize our knowledge on cancer progression and response to treatment. However, up to now, the adaptation, validation and establishment of mechanistic models in predictive medicine has been hindered by the limited availability of data directly linked to the input parameters. Needless to say, in several occasions, no established method exists for the direct measurement of model parameters.

Determinant for the personalized prediction of cancer response is the resistance/sensitivity profile of tumor cells to the specific anti-tumor regimen [11, 42, 43]. *In-vitro* experiments for the evaluation of drug cytotoxicity on cancer cell lines have been proven only indicative, as they cannot reproduce the conditions of tumor microenvironment [44]. On the other hand, mechanistic models may provide means to estimate *in vivo* drug efficacy.

In the present paper, a range of plausible values of the cell killing efficacy of chemotherapy has been estimated for NSCLC treated with various cisplatin-based doublet regimens by using a mechanistic model of cancer response to treatment. The cell killing efficacy of chemotherapy is expressed as the sum of cell kill rates, *CKR*_*sum*_, of the two drugs considered. Our modelling methodology takes into account cell re-population between drug administrations. Parameterization has been driven by principal mechanisms characterizing cancer biology, namely uncontrolled cell proliferation, reversible dormancy, clonal heterogeneity, hypoxia and attenuated apoptosis.

Two types of tumor cell growth have been considered, corresponding to the ADC and SCC histological subtypes, respectively. A comprehensive literature survey has guided the selection of the most appropriate values of characteristic proliferation kinetics for the two histological subtypes. The more aggressive nature of SCC in respect to ADC has been reflected on the higher *T*_*d*_, the higher proliferation, i.e. GF and stem cell frequency, and the greater extend of necrosis. As a first approximation, inter-patient variation in tumor grade has not been taken into account, and a single differentiation profile has been assumed for each histological subtype. Since, in the patient cohort considered, most cancers are moderately to poorly differentiated, terminally differentiated cells have been assumed to comprise the minority of non-dividing cells, with a lifespan of only a few days. As a consequence, the majority of non-dividing cells are found at a reversible dormancy. It is noteworthy that long-lived differentiated cells, with a lifespan of a few months, similar to the normal bronchial epithelium, would most probably be associated with well-differentiated tumors.

A number of sensitivity analyses have been performed in order to investigate the effect of model parameters and cancer proliferation kinetics on the estimation of CKR. OFAT analysis has been chosen as an essential first step in order to gain insight into the mechanisms, verify the biological relevance, validate the assumptions and reveal non-linear patterns or extreme behaviors of individual parameters. In particular, OFAT plots and local sensitivity studies for each model parameter and for each histological subtype have been presented. Subsequently, the OFAT analysis has been supplemented by a number of global sensitivity studies performed by applying the partial rank correlation coefficient (PRCC) method on a latin hypercube sample (LHS) either of the input parameters of the model or of characteristic cancer proliferation kinetics. For the global analyses no distinction between ADC or SCC subtypes has been considered. Although all input parameters are conceptually independent, the fact that the tumor as a whole should satisfy certain macroscopic characteristics (such as volume doubling time, typical fraction of stem cells etc.) dictates numerical correlations among the input parameters. The PRCC method, which by definition estimates parameters’ sensitivity, after controlling for the effects of the remaining parameters, is suitable for models with correlated inputs. A thorough investigation of input correlations is out of the scope of the present work. However, we intend to address it in subsequent publications. The local sensitivity analysis has demonstrated that from the regulating proliferation kinetics those with the highest influence on the estimation of drug efficacy are the fraction of stem cells that perform symmetric divisions and the fraction of newborn cells that withdraw from the active cycle to a dormant phase. Overall, the ADC representative case seems to be less sensitive to all model parameters.

The global sensitivity analysis have revealed that the parameters related to the proliferation kinetics of stem and LIMP cells are the most important determinants of the accuracy of CKR estimates. However, these parameters seem to be reduced to primarily two variables, the T_d_ and the GF at the time of diagnosis. In other words, what is of importance is not only the value of the parameters itself but also the value combination of the parameters that regulate *T*_*d*_ and GF. Therefore uncertainties in the value of the model parameters could be compensated for by an estimation of *T*_*d*_ and GF at diagnosis.

More specifically, the global sensitivity analysis has demonstrated that the rate that a tumor grows in the absence of treatment, expressed by *T*_*d*_, has the strongest effect on the estimation of drug efficacy. This variable reflects the rate at which the tumor replenishes its treatment-induced losses. The estimation of *T*_*d*_ would involve at least two volumetric measurements of the tumor prior to treatment. The use of CT imaging, and especially high-resolution CT, is necessary to derive more accurate estimations that take into consideration the usually asymmetrical growth rate of the tumor in the three dimensions [45]. The interval between CT measurements should be chosen with caution. Yankelevitz et al [46] has suggested that a minimum of 12% increase in the cross-sectional area between successive CT examinations is required for the reliable estimation of *T*_*d*_. For moderate to slow growing tumors an interval of 25 days would be required [45]. Shorter intervals would be needed for fast growing tumors. For example, based on the formula used in [45], a tumor with *T*_*d*_ = 26 days would require a time interval of 6 days between successive CTs. Such waiting periods are common in the case of lung cancer. For half of our patient cohort, the time elapsed between diagnosis and treatment onset is on the order of 20 days and more, so the acquisition of two volumetric measurements prior to treatment might have been feasible.

The GF at diagnosis seems to be the second factor of importance for the estimation of drug efficacy. GF estimations based on Ki-67 protein are routinely performed in clinical practice. In general, a deviation of Ki-67 expression levels between biopsies and surgical samples for the same patients has been observed, which has been attributed to differences in sampling, recruitment and Ki-67 staining procedures [47]. Additionally, since a tumor may include areas of low or high proliferation activity, a single small biopsy may not be accurate. However, a significant correlation for this marker between biopsies and resected tumors has been demonstrated [47, 48]. Another method that has been used in literature for the estimation of GF is the proliferating cell nuclear antigen (PCNA). However, the latter systematically gives higher values than Ki-67 and is considered less reliable [49].

Based on the results of the sensitivity analyses and Fig 7, the duration of cell cycle, *T*_*C*_, seems to have a relatively significant effect on the estimation of treatment efficacy. Many techniques have been reported in literature, either *in vivo* or *in vitro*, for the measurement of cell cycle time, *T*_*C*_, and time-dependent indices in general; however, none has been established and routinely used in clinical practice, mainly due to their slow or laborious nature [23, 50]. The best proposed *in vivo* technique is based on *in vivo* pulse labelling with a thymidine analogue, either bromodeoxyuridine (BrdUrd), or iododeoxyuridine (IdUrd), followed by flow cytometry analysis and application of relative movement (RM) method [23, 50, 51]. It requires just a single biopsy, taken a few hours, e.g. 6–8 hours [23], after BrdUrd (or IdUrd) administration. The method allows for the estimation of the duration of DNA synthesis, *T*_*S*_, and the labelling index, LI, which corresponds to the proportion of cells in S-phase. As a first approximation, *T*_*C*_ can be calculated based on the equation *T*_*C*_ = (*T*_*S*_/LI)*GF, on the condition that the GF is known. More elaborate relationships can be used that take into consideration the non-uniform distribution of an exponentially growing cell population through the cell cycle. In [52] a method to estimate *T*_*C*_ and GF from flow cytometric analysis of a single tumor sample after BrdUrd labelling is proposed.

Apoptosis is the main cell death mechanism triggered by chemotherapy. However, the global and local sensitivity analyses indicate that both the kinetics of apoptosis and the measure of apoptotic index at diagnosis have no effect on the estimation of CKR. The model’s robustness to the kinetics of apoptosis can be explained based on the rapid nature of the phenomenon. Since apoptosis is a process completed within a few hours, it is characterized by a time scale that it is more than one order of magnitude lower than the kinetics of the rest of the cell populations (days to weeks) and the simulation time window, i.e. the time interval between tumor volume measurements (month(s)) or the time interval between the last drug administration and the last volume measurement (week).

The model seems also rather robust to the kinetics of necrosis and the extent of necrosis at diagnosis. It is noted that the results of the local and global sensitivity analyses correspond to relatively fast necrosis kinetics, with *T*_*N*_ not exceeding ten days, and a low to medium extent of necrosis. Furthermore, the importance of CSC resistance in short-term treatment response seems to be moderate due to the very low frequency of cancer stem cells. Finally, for the value range considered, both the local and the global sensitivity analyses demonstrate that the kinetics of terminally differentiated cells have a trivial effect on the CKR estimation. However, as indicated by Fig 3M, a significant sensitivity is expected in the case of long-lived tumor terminally differentiated cells with a lifespan of the order of months. Even though statin has been suggested as a marker of differentiated, non-proliferating cells [53], currently there is no established standard to distinguish terminally differentiated from quiescent cells and, hence, quantify them or measure their kinetics.

Finally, this paper attempted an estimation of cell killing efficacy of cisplatin-based doublet regimen by adapting the model to 13 real clinical cases of NSCLC. The purpose of this process has been the determination of set(s) of values of the input parameters of the model that correspond to the clinical case under test (type of cancer, patient, treatment). CKR estimation can be viewed as a two-step adaptation process. The first step aims at adapting the growth kinetic parameters of the model (Table 4). As the modelling approach provides the ground to simulate tumors of diverse growth kinetic characteristics, by assigning the proper values to the model parameters, narrowing the vast number of possible value combinations, ideally to one, for a specific clinical case is a critical first step. In this context, the exploitation of measurable proliferation indices, such as Ki-67, or data that could allow the precise estimation of the tumor growth rate, such as at least two imaging data before therapy, is decisive. However, due to non-availability of patient-specific tumor cell kinetics data, a literature review survey has provided biologically reasonable values for critical proliferation features.

The second step involves the adaptation of pharmacokinetic and pharmacodynamic parameters of the model in the case of chemotherapy. The driver in this process is the achievement of volumetric match between the clinical data, i.e. the observed shrinkage in volume, and the result of the simulation, after applying the treatment regimen that was administered in the clinical case under test. The pharmacokinetic and pharmacodynamic properties of the agents considered were summarized under the umbrella of one parameter, the apparent CKR. The purpose of the adaptation process was to evaluate a range of CKR values of the above parameter for the clinical case under test (treatment regimen, dose, patient) based on the results of the adjustment of the first step and the recorded change in tumor size due to the treatment.

Since the regimen administered consists of two chemotherapeutic agents, it is not possible to accurately determine the CKR of each drug from the data provided, even in the ideal case of the availability of all required proliferation indices and tumor free growth kinetic features that would enable an excellent fitting of the model parameters to the clinical case examined. In the study presented here an ‘apparent’ combination of the CKR of the drugs involved, for the virtual tumor implementations considered, is determined, by assuming an arbitrary value for one of the drugs. In the clinical data set considered, the majority of the patients (6/13) appeared to have a moderate *CKR*_*sum*_, whereas 2/13 patients had a very low *CKR*_*sum*_ and one had a very high *CKR*_*sum*_. Based on the results, the cell killing efficacy of treatment varies considerably among the patients, a fact that verifies the importance of a timely estimation of treatment response through *in silico* simulations.

The accuracy of the conclusions on the treatment effect that was attempted in this paper was subject to the uncertainties with respect to the tumor kinetics. As the number of available data was the minimum required for the model adaptation, averages or ranges that were encountered in the literature were used. More specifically, the data were limited to the assessment of the tumor size in two time points (one before and the other after or during treatment) and to information about the treatment, whereas the data regarding the proliferation features of the tumor was absent. It is noteworthy that the provision of additional tumor size measurements, during the course of therapy, could allow for more accurate estimates. Furthermore, any errors in the estimation of the tumor size were not taken into account, as it is estimated that the uncertainty that they can introduce is much smaller than the lack of knowledge of the characteristics of free tumor growth.

Despite the aforementioned sources of uncertainties, the short-term predictive potential of our model has been clearly demonstrated (Fig 8). The gross agreement between the tumor size predicted based on our CKR estimates and the real post-surgery measured one (Fig 8), substantiates the potential utility of the proposed cancer model/methodology for personalized predictions of treatment response at diagnosis. Studies between *in vivo CKR* estimates, as attempted in the present work, and the molecular profile of a patient, hold great potential for the identification of resistance and sensitivity profiles. For that purpose, statistical learning techniques, either classification or regression ones, can be recruited to distinguish among drug sensitivity phenotypes and to quantify tumor cell response to treatment, based on whole genome analysis data, gene expression profiles and transcriptomic or proteomic signatures. *In vivo CKR* estimates can be utilized together with the molecular data to train these machine learning models. Eventually, such studies could allow for the prediction of treatment outcome based on the molecular or genetic data of a newly diagnosed patient and will constitute a future step of our work. Our preliminary efforts in this direction [54, 55], attempting to correlate prednisone *CKR* estimates with the pathway gene expression data in paediatric acute lymphoblastic leukaemia, have shown promising first results.

## Supporting Information

S1 Text Discussion on delays in treatment-induced apoptosis due to pharmacokinetics and pharmacodynamics. (PDF)

S2 Text Estimation of initial tumor cell composition (at balanced exponential growth). (PDF)

S3 Text Literature Survey: Proliferation features of non-small cell lung cancer (NSCLC). (PDF)

S4 Text Range sensitivity analysis. (PDF)

S5 Text Value range of spontaneous apoptosis rate. (PDF)

S1 Fig Effect of the cell cycle time of stem and LIMP cells on the tumor doubling time. The values assigned to the rest of the model input parameters correspond to the baseline values of SCC (Table 5). Abbreviations: LIMP: LImited Mitotic Potential tumor cell (also called committed or restricted progenitor cell), SCC: Squamous Cell Carcinoma.
(TIF)

S2 Fig Effect of the cell cycle time of stem and LIMP cells on the initial growth fraction. The values assigned to the rest of the model input parameters correspond to the baseline values of SCC (Table 5). Abbreviations: LIMP: LImited Mitotic Potential tumor cell (also called committed or restricted progenitor cell), SCC: Squamous Cell Carcinoma.
(TIF)

S3 Fig Effect of the number of mitoses between stem and terminally differentiated cells on the estimation of the sum of cisplatin and gemcitabine cell kill rates. The rest of the model parameters are kept constant at a baseline value. Stem and LIMP cells are assumed to be equally sensitive to treatment, i.e. the cell kill factor of stem cells, CKF, is set equal to unity. Two sets of baseline values have been considered for the rest of the model input parameters, corresponding to a SCC and an ADC representative case (Table 5). Abbreviations: LIMP: LImited Mitotic Potential tumor cell (also called committed or restricted progenitor cell), ADC: Adenocarcinoma, SCC: Squamous Cell Carcinoma.
(TIF)

S4 Fig Scatterplot of the fraction of stem cells vs. the number of mitoses between stem and terminally differentiated cells (*N*_*LIMP*_). Latin Hypercube Sampling has run to produce two sets of 8000 combinations of model parameters (Table 4), with the given value ranges (cases A and B respectively). Combinations with negative cell proliferation kinetics have been excluded. The red line corresponds to the upper limit of 0.001 considered in the present study for the cancer stem cell fraction. We observe that for values of *N*_*LIMP*_ lower than 8, this limit is always exceeded. Abbreviations: LIMP: LImited Mitotic Potential tumor cell (also called committed or restricted progenitor cell).
(TIF)

S1 Table Supplemental PRCC analyses. (PDF)

S1 File Image files. (ZIP)
