# Supplementary material for: In Silico Oncology: Quantification of the In Vivo Antitumor Efficacy of Cisplatin-Based Doublet Therapy in Non-Small Cell Lung Cancer (NSCLC) through a Multiscale Mechanistic Model
Source: PLoS Comput Biol. 2016 Sep 22;12(9):e1005093. doi: 10.1371/journal.pcbi.1005093 (PMC5033576; doi:10.1371/journal.pcbi.1005093)
Supplement: S1 Text — (PDF) [file pcbi.1005093.s001.pdf]

Eleni Kolokotroni, Dimitra Dionysiou, Christian Veith, Yoo-Jin Kim, Jörg Sabczynski, Astrid Franz, Aleksandar Grgic, Jan Palm, Rainer M. Bohle, Georgios Stamatakos

S1 Text

Discussion on delays in treatment-induced apoptosis due to pharmacokinetics and pharmacodynamics

A. Time delays vs. final tumor volumetric measurement (CT acquisition)

Let's assume a hypothetical tumor with an initial population of 100 cells. The tumor is treated with a single dose of chemotherapy corresponding to a cell kill rate (CKR) equal to 0.4. The chemo kills 40 cancer cells, resulting in a final population of 60 tumor cells. Let's also assume that in the simulated tumor, lethally hit cells disappear at the instance of drug administration. In reality, lethally hit cells will occupy space in the tumor for some time before their permanent removal. This time will depend on the pharmacokinetics and pharmacodynamics of the drug. If CT acquisition takes place while the effect of treatment is still ongoing, e.g. when only 20 out of 40 cells have disappeared and, hence, the tumor consists of 80 cells, then the estimated CKR will be less, because it will be derived assuming a higher final volume. On the other hand, if the tumor is measured after the completion of the treatment effect, then the estimated CKR will be accurate, regardless of the exact time point of measurement. It should be noted that, in the above example, the proliferation during and after treatment is ignored for simplicity reasons, without loss of generality; however, it is explicitly modelled in our approach. Moreover, in our approach, lethally hit cells are not removed from the tumor instantaneously but follow a rudimentary cell cycle that leads them to apoptotic death.

Additional time delays in the permanent removal of lethally hit cells are expected due to the method of administration (infusion in our cases), and the specific pharmacokinetics and pharmacodynamics of the drug. Table A and B lists characteristic pharmacokinetic and pharmacodynamic properties of the drugs considered in the present work. For example, in the case of cisplatin, maximum plasma concentration is reached at the end of infusion (pharmacokinetics) [Delord *et al.*, 2009], thus 1 hour after drug administration, whereas the drug may cause a cell arrest of a couple of days (pharmacodynamics), before the triggering of the apoptotic mechanism, in an attempt of the cell to repair its damages. The consideration of these delays are determinant when the final CT acquisition takes place while the effect of therapy is still ongoing. However, in all cases, the final CT acquisition takes place in the very late terminal phase of elimination of the last drug dose and the interval between final CT acquisition and last drug administration is long enough relative to both the terminal half-life of the drug (Table C) and the possible delays due to the drug pharmacodynamics (Table B). It should be noted that this late terminal phase has no therapeutic significance but rather represents drug persistence in body.

B. Time delays vs. dosage intervals

In all dosage regimens, the interval between consecutive administrations of the same drug is large relative to terminal half-life (Table C). The doses are administered in the very late terminal phase of elimination of the previous one, when the residual plasma concentration is either below the detection limit (e.g. cisplatin, gemcitabine) or corresponds to a very small fraction (<1%) of the maximum concentration (e.g. vinorelbine on day 8 of each cycle and docetaxel). Therefore, no drug accumulation is anticipated [Delord *et al.*, 2009; Fan *et al.*, 2010; Brunsvig *et al.*, 2007; de Lange *et al.*, 2005].

Table A. Pharmacokinetic properties of cisplatin, docetaxel, gemcitabine and vinorelbine.

Drug	References	No of patients	Dose (mg/m^2)	Infusion time	Terminal half-life[b], $t_{1/2}$	Residual[e] plasma[f]/blood[g] concentration (ng/ml)	Comment
**Cisplatin[a]**	Watanabe *et al.*, 2003	3	80	30 min	0.70±0.22 h	Day 2 - 14: below detection limit[f]	For patients with normal renal function
	Schellens *et al.*, 1996	45	70, 80		38 ± 10 min, (23 – 72) min		
	Delord *et al.*, 2009	11	100	1h	Cycle 1: 0.865± 0.198 h<br>Cycle 2: 0.777 ±0.150 h<br>Cycle 3: 0.756± 0.058		Pharmacokinetic parameters not statistically different amongst the 3 cycles
	Vermorken *et al.*, 1986	11	60-100	6 min-24 h	(26.0 - 78.8) min		
**Docetaxel**	Felici *et al.*, 2006	9	75	1 h	11.7± 7.1 h<br>(4.0–24.0) h		
	Baker *et al.*, 2004	9	75	1.04± 0.036h [b]	17.5 ± 7.3 h[c]<br>91.7±32.1[d]	Day 22: 0.47±0.08 (~0.02% of C_{max})[f]	Concentrations on day 22 were below the lower limit of quantitation in 5 of 9 patients
	Brunsvig *et al.*, 2007	19	100	1h	18 (9-52)		The median ratio for AUCs of the second to the first cycle was close to 1
**Gemcitabine**	de Lange *et al.*, 2005	14	1000	30 min	11-26 min	Day 8: 0 [f]	No drug accumulation (weekly administration)<br>C_{max} observed at the end of the infusion
	Fan *et al.*, 2010	7	1250	30 min	Day 1: 0.42± 0.20 h<br>Day 5:0.67± 0.31 h	Day 5: 0 [f]	Pharmacokinetic parameters not statistically different between first (day 1) and second (day 5) dose administration
	Gan *et al.* 2006	9	1250	30 min	11±4 min		
**Vinorelbine**	Khayat *et al.*, 2004	18	30	20 min	32.0 ± 14.2 h	Day 3: ~ 3.6 (~0.35% of C_{max}) [g]	
	Lush *et al.*, 2005	24	30	20 min	49.13±10.04 h (36.07–73.38)	Day 8: ~0.6 (~0.03% of C_{max}) [g]	
	Delord *et al.*, 2009	11	60	oral			Pharmacokinetic parameters not statistical different between day 1 and day 8 of vinorelbine administration in the same cycle, or between the 3 cycles

[a] pharmacologically active unbound form of cisplatin in plasma, [b] Data represent mean ± SD (range), [c] Based on sampling up to 24h post-treatment, [d] Based on extended sampling up to 22 days post-treatment, [e] Drug administration takes place in day 1, C_{max}: maximum plasma concentration, AUC: Area under the plasma concentration vs. time curve

Table B. Pharmacodynamic properties of cisplatin, docetaxel, gemcitabine and vinorelbine.

Drug	Onset of apoptosis after treatment	References
**Cisplatin**	96h	Sorenson *et al.*, 1990
**Docetaxel**	24-48 h	Fabbri *et al.*, 2006; Fabbri *et al.*, 2008
**Gemcitabine**	0-48 h	Cappella *et al.*, 2001
**Vinorelbine**	~ 8 h	Aggarwal *et al.*, 2008

Table C. Comparison of time scales

Drug	Dosage interval, τ_D (days)	Interval between last administration and final CT acquisition, τ_A (days)	Typical terminal half-life, $t_{1/2}$ (hours)	$\tau_D/t_{1/2}$	$\tau_A/t_{1/2}$
**Cisplatin**	21-35	19-28 (one patient 7)	0.7	>720	>240
**Docetaxel**	21	19	17	30	27
**Gemcitabine**	Same cycle:7-8<br>Between cycles:14	12-14	0.4	Same cycle:>420<br>Between cycles: 840	>720
**Vinorelbine**	Same cycle:7<br>Between cycles:14-28	13-21 (one patient 7)	40	Same cycle:4.2<br>Between cycles:8.4-16.8	12.6 (4.2)

References

Aggarwal A, Kruczynski A, Frankfurter A, Correia JJ, Lobert S. Murine leukemia P388 vinorelbine-resistant cell lines are sensitive to vinflunine. Invest New Drugs. 2008 Aug;26(4):319-30.

Baker SD, Zhao M, Lee CK, Verweij J, Zabelina Y, Brahmer JR, Wolff AC, Sparreboom A, Carducci MA. Comparative pharmacokinetics of weekly and every-three-weeks docetaxel. Clin Cancer Res. 2004 Mar 15;10(6):1976-83.

Brunsvig PF, Andersen A, Aamdal S, Kristensen V, Olsen H. Pharmacokinetic analysis of two different docetaxel dose levels in patients with non-small cell lung cancer treated with docetaxel as monotherapy or with concurrent radiotherapy. BMC Cancer. 2007 Oct 23;7:197.

Cappella P, Tomasoni D, Faretta M, Lupi M, Montalenti F, Viale F, et al. Cell cycle effects of gemcitabine. Int J Cancer. 2001 Aug 1;93(3):401-8.

Delord JP, Puozzo C, Lefresne F, Bugat R. Combination chemotherapy of vinorelbine and cisplatin: a phase I pharmacokinetic study in patients with metastatic solid tumors. Anticancer Res. 2009 Feb;29(2):553-60.

Fabbri F, Carloni S, Brigliadori G, Zoli W, Lapalombella R, Marini M: Sequential events of apoptosis involving docetaxel, a microtubule-interfering agent: a cytometric study. BMC Cell Biology. 2006, 7: 6-10.1186/1471-2121-7-6

Fabbri F, Amadori D, Carloni S, Brigliadori G, Tesei A, Ulivi P, Rosetti M, Vannini I, Arienti C, Zoli W, Silvestrini R: Mitotic catastrophe and apoptosis induced by docetaxel in hormone-refractory prostate cancer cells. J Cell Physiol. 2008, 217: 494-501. 10.1002/jcp.21522

Fan Y, Lin NM, Ma SL, Luo LH, Fang L, Huang ZY, Yu HF, Wu FQ. Phase II trial of gemcitabine plus cisplatin in patients with advanced non-small cell lung cancer. Acta Pharmacol Sin. 2010 Jun;31(6):746-52. doi: 10.1038/aps.2010.50.

Felici A, Loos WJ, Verweij J, Cirillo I, de Bruijn P, Nooter K, Mathijssen RH, de Jonge MJ. A pharmacokinetic interaction study of docetaxel and cisplatin plus or minus 5-fluorouracil in the treatment of patients with recurrent or metastatic solid tumors. Cancer Chemother Pharmacol. 2006 Nov;58(5):673-80.

Gan HK, Mitchell PL, Galettis P, Davis ID, Cebon J, de Souza P, Links M. A phase 1 and pharmacokinetic study of gemcitabine and oxaliplatin in patients with solid tumors. Cancer Chemother Pharmacol. 2006 Aug;58(2):157-64. Epub 2005 Nov 19.

Khayat D, Rixe O, Brunet R, Goupil A, Bugat R, Harousseau JL, Ifrah N, Puozzo C. Pharmacokinetic linearity of i.v. vinorelbine from an intra-patient dose escalation study design. Cancer Chemother Pharmacol. 2004 Sep;54(3):193-205.

de Lange SM, van der Born K, Kroep JR, Jensen HA, Pfeiffer P, Cleverly A, van Groeningen CJ, Peters GJ. No evidence of gemcitabine accumulation during weekly administration. Eur J Clin Pharmacol. 2005 Dec;61(11):843-9.

Lush RM, McCune JS, Tetteh L, Thompson JA, Mahany JJ, Garland L, Suttle AB, Sullivan DM. The absolute bioavailability of oral vinorelbine in patients with solid tumors. Cancer Chemother Pharmacol. 2005 Dec;56(6):578-84.

Schellens JH, Ma J, Planting AS, van der Burg ME, van Meerten E, de Boer-Dennert M, Schmitz PI, Stoter G, Verweij J. Relationship between the exposure to cisplatin, DNA-adduct formation in leucocytes and tumour response in patients with solid tumours. Br J Cancer. 1996 Jun;73(12):1569-75.

Sorenson CM, Barry MA and Eastman A. Analysis of Events Associated With Cell Cycle Arrest at G2 Phase and Cell Death Induced by Cisplatin. J Natl Cancer Inst. 1990 May 2;82(9):749-55

Vermorken JB, van der Vijgh WJ, Klein I, Gall HE, van Groeningen CJ, Hart GA, Pinedo HM. Pharmacokinetics of free and total platinum species after rapid and prolonged infusions of cisplatin. Clin Pharmacol Ther. 1986 Feb;39(2):136-44.

Watanabe R, Takiguchi Y, Moriya T, Oda S, Kurosu K, Tanabe N, Tatsumi K, Nagao K, Kuriyama T. Feasibility of combination chemotherapy with cisplatin and etoposide for haemodialysis patients with lung cancer. Br J Cancer. 2003 Jan 13;88(1):25-30.
