# Supplementary material for: In Silico Oncology: Quantification of the In Vivo Antitumor Efficacy of Cisplatin-Based Doublet Therapy in Non-Small Cell Lung Cancer (NSCLC) through a Multiscale Mechanistic Model
Source: PLoS Comput Biol. 2016 Sep 22;12(9):e1005093. doi: 10.1371/journal.pcbi.1005093 (PMC5033576; doi:10.1371/journal.pcbi.1005093)
Supplement: S2 Text — (PDF) [file pcbi.1005093.s002.pdf]

Eleni Kolokotroni, Dimitra Dionysiou, Christian Veith, Yoo-Jin Kim, Jörg Sabczynski, Astrid Franz, Aleksandar Grgic, Jan Palm, Rainer M. Bohle, Georgios Stamatakos

# S2 Text

# Estimation of initial tumor cell composition (at balanced exponential growth)

At conditions of balanced exponential growth all tumor cell populations grow with the same rate constant and their fraction in the tumor bulk is invariant with time. In our modelling approach, the simulated tumor is initialized with the population fractions that the cancerous system tends to establish at balanced exponential growth, for the given values of input parameters. Prior to simulation, these fractions are computationally estimated [Stamatakos *et al.*, 2010; Kolokotroni *et al.*, 2011]. In the present section analytical expressions describing the proportion of the various tumor cell categories are derived, based on a compartmental analysis of the adopted kinetic model of tumor cells. All cell category populations are expressed in respect to the total proliferating cells, unless otherwise stated.

## 1. Dormant cells

According to the analysis in Kolokotroni *et al.* (2011) and Bertuzi *et al.* (1997), the population of stem proliferating $N_P(\tau,t)$ and dormant $N_{G0}(t)$ cells, assuming conditions of balanced exponential growth, are given by Eqs (1) and (2) respectively:

$$N_P(\tau,t) = \overline{N}_P e^{-(a+R_A)\tau} e^{at} \tag{1}$$

$$N_{G0}(t) = \overline{N}_{G0} e^{at} \tag{2}$$

where $\tau \in [0, T_c]$ is the time elapsed since proliferating cells have entered the cell cycle, $\overline{N}_P$ and $\overline{N}_{G0}$ are the initial populations (at $t=0$ and $\tau=0$), and $a$ is the growth rate constant. The total population of proliferating cells at time $t$ is derived by integrating Eq (1) with respect to $\tau$, over the cell cycle duration $T_c$:

$$N_P(t) = \int_0^{Tc} N_P(\tau,t)d\tau = \int_0^{Tc} \overline{N}_P e^{-(a+R_A)\tau} e^{at} d\tau \Rightarrow$$

$$N_P(t) = \overline{N}_P \frac{(1-e^{-(a+R_A)Tc})}{a+R_A} e^{at} \tag{3}$$

The ratio between the initial populations can be derived by Eq (6) in Kolokotroni *et al.* (2011):

$$\frac{\overline{N}_{G0}}{\overline{N}_P} = \frac{(1+P_{sym})P_{sleep} e^{-(a+R_A)T_C}}{\left(a+R_A+\dfrac{1}{T_{G0}}\right)} \tag{4}$$

Based on Eqs (2) and (3) and by taking into account Eq (4) the ratio between dormant and proliferating cells can be computed:

$$\frac{N_{G0}(t)}{N_P(t)} = \frac{\overline{N}_{G0} e^{at}}{\overline{N}_P \dfrac{(1-e^{-(a+R_A)Tc})}{a+R_A} e^{at}} = \frac{\overline{N}_{G0}}{\overline{N}_P} \frac{a+R_A}{(1-e^{-(a+R_A)Tc})} \Rightarrow$$

$$\frac{N_{G0}(t)}{N_P(t)} = (1+P_{sym})P_{sleep}\frac{a+R_A}{\left(a+R_A+\dfrac{1}{T_{G0}}\right)}\frac{e^{-(a+R_A)T_C}}{\left(1-e^{-(a+R_A)Tc}\right)} \Rightarrow$$

$$\frac{N_{G0}(t)}{N_P(t)} = (1+P_{sym})P_{sleep}\frac{a+R_A}{\left(a+R_A+\dfrac{1}{T_{G0}}\right)}\frac{1}{\left(e^{(a+R_A)Tc}-1\right)} \quad (5)$$

In the present analysis stem and LIMP cells are considered to be of equal cell cycle duration and of equal residence time in dormant phase. Thus, Eq (5) is valid not only for stem cells but for the total population of proliferating and dormant cells (stem and LIMP).

## 2. Terminally differentiated cells

In the following analysis the compartment of DIFF cells perceives the population of proliferating cells, either stem or LIMP, as one compartment. Taking into account that (a) the terminally differentiated cells may die through spontaneous apoptosis or necrosis and, hence, are removed from the corresponding compartment at rates equal to $R_{ADiff}$ και $R_{NDiff}$ respectively, and (b) at time $t$ a fraction equal to (1-$P_{sym}$) out of the dividing population $N_P(T_c,t)$ of stem cells will follow the path towards differentiation, the population of DIFF cells $N_{Diff}(t)$ must satisfy the following equation:

$$\frac{d}{dt}N_{Diff}(t) = (1-P_{sym})N_P(T_c,t) - (R_{ADiff}+R_{NDiff})N_{Diff}(t) \quad (6)$$

Under conditions of balanced exponential growth with growth rate constant $\alpha$, the time course of the DIFF cell population is given by:

$$N_{Diff}(t) = \overline{N}_{Diff}e^{at} \quad (7)$$

where $\overline{N}_{Diff}$ the initial population. By substituting in Eq (6) the populations of proliferating and DIFF cells as given by Eqs (1) and (7) respectively, we obtain the ratio $\overline{N}_{Diff}/\overline{N}_P$ :

$$\alpha\overline{N}_{Diff}e^{\alpha t} = (1-P_{sym})\overline{N}_P e^{-(a+R_A)T_C}e^{at} - (R_{ADiff}+R_{NDiff})\overline{N}_{Diff}e^{\alpha t} \Rightarrow$$

$$\frac{\overline{N}_{Diff}}{\overline{N}_P} = \frac{(1-P_{sym})e^{-(a+R_A)T_C}}{(\alpha+R_{ADiff}+R_{NDiff})} \quad (8)$$

The ratio between terminally differentiated and proliferating cells can be calculated by Eqs (3), (7) and (8):

$$\frac{N_{Diff}(t)}{N_P(t)} = \frac{\overline{N}_{Diff}e^{\alpha t}}{\overline{N}_P\dfrac{(1-e^{-(\alpha+R_A)T_C})}{\alpha+R_A}e^{\alpha t}} = (1-P_{sym})\frac{\alpha+R_A}{\alpha+R_{ADiff}+R_{NDiff}}\frac{e^{-(\alpha+R_A)T_C}}{1-e^{-(\alpha+R_A)T_C}} \Rightarrow$$

$$\frac{N_{Diff}(t)}{N_P(t)} = (1-P_{sym})\frac{\alpha+R_A}{\alpha+R_{ADiff}+R_{NDiff}}\frac{1}{e^{(\alpha+R_A)T_C}-1} \quad (9)$$

## 3. Apoptotic cells

The compartment of apoptotic cells is populated from proliferating and dormant cells with a rate constant $R_A$ and from terminally differentiated cells with a rate constant $R_{ADiff}$. The residence time of apoptotic cells in their compartment, defined as the time needed for apoptosis process to be completed and apoptotic bodies to be removed from the tumor bulk, is $T_A$. Hence, the removal rate constant of apoptotic cells is equal to $1/T_A$. Under the preceding assumptions the apoptotic cell population, $N_A(t)$, must satisfy the following equation:

$$\frac{d}{dt}N_A(t) = R_A N_P(t) + R_A N_{G0}(t) + R_{ADiff} N_{Diff}(t) - \frac{1}{T_A} N_A(t) \tag{10}$$

Under conditions of balanced growth the time course of apoptotic cell population is given by:

$$N_A(t) = \overline{N}_A e^{at} \tag{11}$$

where $\overline{N}_A$ the initial population. From Eqs (11) and (10) it follows:

$$\alpha \overline{N}_A e^{\alpha t} = R_A N_P(t) + R_A N_{G0}(t) + R_{ADiff} N_{Diff}(t) - \frac{1}{T_A} N_A(t) \Rightarrow$$

$$\alpha N_A(t) = R_A N_P(t) + R_A N_{G0}(t) + R_{ADiff} N_{Diff}(t) - \frac{1}{T_A} N_A(t) \Rightarrow$$

$$(\alpha + \frac{1}{T_A}) N_A(t) = R_A N_P(t) + R_A N_{G0}(t) + R_{ADiff} N_{Diff}(t) \Rightarrow$$

$$\frac{N_A(t)}{N_P(t)} = \frac{R_A}{(\alpha + \frac{1}{T_A})} + \frac{R_A}{(\alpha + \frac{1}{T_A})} \frac{N_{G0}(t)}{N_P(t)} + \frac{R_{ADiff}}{(\alpha + \frac{1}{T_A})} \frac{N_{Diff}(t)}{N_P(t)} \tag{12}$$

By substituting in (12) the fraction of dormant and differentiated cells in respect to proliferating cells from Eqs (5) and (9), respectively, we obtain:

$$\frac{N_A(t)}{N_P(t)} = \frac{R_A}{(\alpha + \frac{1}{T_A})} + \frac{R_A}{(\alpha + \frac{1}{T_A})} \left(1 + P_{sym}\right) P_{sleep} \frac{a + R_A}{\left(a + R_A + \frac{1}{T_{G0}}\right)} \frac{1}{\left(e^{(a+R_A)Tc} - 1\right)} + \frac{R_{ADiff}}{(\alpha + \frac{1}{T_A})} (1 - P_{sym}) \frac{\alpha + R_A}{\alpha + R_{ADiff} + R_{NDiff}} \frac{1}{e^{(\alpha + R_A)T_C} - 1} \tag{13}$$

## 4. Necrotic cells

Necrotic cells are populated from the compartments of dormant and terminally differentiated cells. The residence time of tumor cells in dormant phase is $T_{G0}$, and when this time expires the cells will either become cycling, with a probability $P_{G0toG1}$, or will enter the necrotic pathway with a probability 1-$P_{G0toG1}$. Hence, the transition rate constant between the compartments of dormant and necrotic cells is (1-$P_{G0toG1}$)$/T_{G0}$. Moreover differentiated cells die through necrosis with a rate constant $R_{NDiff}$. Necrotic cells are permanently removed from the tumor bulk with a rate constant $1/T_N$, where $T_N$, the time needed for

the completion of necrosis and the removal of lysis' products.Under the above assumptions the necrotic cell population, $N_N(t)$, must satisfy the following equation:

$$\frac{d}{dt}N_N(t)=\frac{(1-P_{G0toG1})}{T_{G0}}N_{G0}(t)+R_{NDiff}N_{Diff}(t)-\frac{1}{T_N}N_N(t) \tag{14}$$

As previously, under conditions of balanced growth we have:

$$N_N(t)=\overline{N}_N e^{at} \tag{15}$$

From Eqs (14) and (15) it yields:

$$\alpha\overline{N}_N e^{\alpha t}=\frac{(1-P_{G0toG1})}{T_{G0}}N_{G0}(t)+R_{NDiff}N_{Diff}(t)-\frac{1}{T_N}N_N \Rightarrow$$

$$\alpha N_N(t)=\frac{(1-P_{G0toG1})}{T_{G0}}N_{G0}(t)+R_{NDiff}N_{Diff}(t)-\frac{1}{T_N}N_N \Rightarrow$$

$$(\alpha+\frac{1}{T_N})N_N(t)=\frac{(1-P_{G0toG1})}{T_{G0}}N_{G0}(t)+R_{NDiff}N_{Diff}(t)\Rightarrow$$

$$\frac{N_N(t)}{N_P(t)}=\frac{(1-P_{G0toG1})}{T_{G0}(\alpha+\frac{1}{T_N})}\frac{N_{G0}(t)}{N_P(t)}+\frac{R_{NDiff}}{(\alpha+\frac{1}{T_N})}\frac{N_{Diff}(t)}{N_P(t)} \tag{16}$$

By substituting in (16) the fraction of dormant and differentiated cells in respect to proliferating cells from Eqs (5) and (9), we obtain:

$$\frac{N_N(t)}{N_P(t)}=\frac{(1-P_{G0toG1})}{T_{G0}(\alpha+\frac{1}{T_N})}(1+P_{sym})P_{sleep}\frac{a+R_A}{\left(a+R_A+\frac{1}{T_{G0}}\right)}\frac{1}{\left(e^{(a+R_A)Tc}-1\right)}+ \\ +\frac{R_{NDiff}}{(\alpha+\frac{1}{T_N})}(1-P_{sym})\frac{\alpha+R_A}{\alpha+R_{ADiff}+R_{NDiff}}\frac{1}{e^{(\alpha+R_A)T_C}-1} \tag{17}$$

## 5. Stem and LIMP cells

In the following analysis, stem and LIMP cells are considered to be of equal cell cycle duration and of equal residence time in dormant phase. Let $N$ be the number of mitoses performed by LIMP cells before becoming terminally differentiated. The population of stem cells constitutes one compartment, while LIMP cells are divided into as many compartments as the number of mitoses $N$. Each compartment of class $n$, $n$=0,…,$N$-1, contains the LIMP cells that have performed the same number $n$ of mitotic divisions. Let $N_{S,P}$ and $N_{S,G0}$ be the number of stem cells residing in the active cell cycle and dormant (quiescent) phase, respectively, whereas $N_{Ln,P}$ and $N_{Ln,G0}$ represent the number of proliferating and dormant LIMP cells that have undergone $n$ mitoses. Therefore, the compartment of class $n$=0 corresponds to the LIMP cells that have arisen directly from the asymmetric division of stem cells and have performed no mitotic division yet, while the compartment of class $n$=$N$-1 contains the LIMP cells that have one more mitotic division left before their terminal differentiation. We assume that the number of proliferating cells, stem or LIMP, is a function of two independent time variables: the time $t$ measured from the beginning of the simulation and the time $\tau$ that has elapsed since the cells have entered the cell cycle, $\tau \in [0, T_c]$. At each time $t$, the number of stem cells that divide is $N_{S,P}(T_c,t)$. A fraction equal to (1-$P_{sym}$)*$N_{S,P}(T_c,t)$ will give birth to one stem cell and one LIMP cell of $n$=0 class. LIMP cells perform only symmetric divisions giving birth to LIMP cells that are placed in the next compartment. According to the above described kinetic model, the populations of stem and LIMP cells must satisfy the following equation:

*Stem cells:*

$$\frac{\partial}{\partial t}N_{S,P}(\tau,t)+\frac{\partial}{\partial \tau}N_{S,P}(\tau,t)=-R_A N_{S,P}(\tau,t) \tag{18}$$

$$\frac{d}{dt}N_{S,G0}(t)=(1+P_{sym})P_{sleep}N_{S,P}(T_C,t)-\left(R_A+\frac{1}{T_{G0}}\right)N_{S,G0}(t) \tag{19}$$

with boundary condition

$$N_{S,P}(0,t)=(1+P_{sym})(1\text{-}P_{sleep})\,N_{S,P}(T_C,t)+\left(\frac{P_{G0toG1}}{T_{G0}}\right)N_{S,G0} \tag{20}$$

*LIMP cells of class n=0:*

$$\frac{\partial}{\partial t}N_{L0,P}(\tau,t)+\frac{\partial}{\partial \tau}N_{L0,P}(\tau,t)=-R_A N_{L0,P}(\tau,t) \tag{21}$$

$$\frac{d}{dt}N_{L0,G0}(t)=(1-P_{sym})P_{sleep}N_{S,P}(T_C,t)-\left(R_A+\frac{1}{T_{G0}}\right)N_{L0,G0}(t) \tag{22}$$

with boundary condition

$$N_{L0,P}(0,t)=(1-P_{sym})(1\text{-}P_{sleep})\,N_{S,P}(T_C,t)+\left(\frac{P_{G0toG1}}{T_{G0}}\right)N_{L0,G0} \tag{23}$$

*LIMP cells of n class:*

$$\frac{\partial}{\partial t}N_{Ln,P}(\tau,t)+\frac{\partial}{\partial \tau}N_{Ln,P}(\tau,t)=-R_A N_{Ln,P}(\tau,t), \quad n=1,\ldots,N-1 \tag{24}$$

$$\frac{d}{dt}N_{Ln,G0}(t)=2*P_{sleep}N_{Ln-1,P}(T_C,t)-\left(R_A+\frac{1}{T_{G0}}\right)N_{Ln,G0}(t), \quad n=1,\ldots,N-1 \tag{25}$$

with boundary condition

$$N_{Ln,P}(0,t) = 2*(1\text{-}P_{sleep})\, N_{Ln-1,P}(T_C,t) + \left(\frac{P_{G0toG1}}{T_{G0}}\right) N_{Ln,G0}, \quad n=1,\dots,N-1 \tag{26}$$

Under conditions of balanced exponential growth, all populations expand with the same growth rate *α*. Their time courses are given by:

$$N_{S,P}(\tau,t) = \overline{N}_{S,P} e^{-(a+R_A)\tau} e^{at} \tag{27}$$

$$N_{S,G0}(t) = \overline{N}_{S,G0} e^{at} \tag{28}$$

$$N_{Ln,P}(\tau,t) = \overline{N}_{Ln,P} e^{-(a+R_A)\tau} e^{at}, \quad n=0,\dots,N-1 \tag{29}$$

$$N_{Ln,G0}(t) = \overline{N}_{Ln,G0} e^{at}, \quad n=0,\dots,N-1 \tag{30}$$

where $\overline{N}_{S,P}$, $\overline{N}_{S,G0}$, $\overline{N}_{Ln,P}$ και $\overline{N}_{Ln,G0}$ the initial populations (for *t=0* and/or *τ=0).* The total population of proliferating cells at time *t* is derived by integrating Eqs (27) and (29) in respect to *τ*, over the cell cycle duration $T_c$. It yields:

$$N_{S,P}(t) = \overline{N}_{S,P} \frac{(1-e^{-(a+R_A)Tc})}{a+R_A} e^{at} \tag{31}$$

$$N_{Ln,P}(t) = \overline{N}_{Ln,P} \frac{(1-e^{-(a+R_A)Tc})}{a+R_A} e^{at}, \quad n=0,\dots,N-1 \tag{32}$$

### 5.1 Ratio of zero class LIMP cells to stem cells

We will compute the ratio between the LIMP cells of *n*=0 class and the stem cells:

$$\frac{N_{L0}(t)}{N_S(t)} = \frac{N_{L0,P}(t) + N_{L0,G0}(t)}{N_{S,P}(t) + N_{S,G0}(t)}$$

By substituting in Eq (19) the population of stem proliferating and dormant cells from Eqs (27) and (28), we obtain:

$$\overline{N}_{S,G0} = (1+P_{sym}) \frac{P_{sleep}}{\left(a + R_A + \frac{1}{T_{G0}}\right)} e^{-(a+R_A)T_C}\, \overline{N}_{S,P} \tag{33}$$

By substituting in Eq (22) the population of LIMP proliferating and dormant cells from Eqs (29) and (30), we get:

$$\overline{N}_{L0,G0} = (1-P_{sym}) \frac{P_{sleep}}{\left(a + R_A + \frac{1}{T_{G0}}\right)} e^{-(a+R_A)T_C}\, \overline{N}_{S,P} \tag{34}$$

Based on the above, the ratio between stem and LIMP dormant cells of *n*=0 class can be calculated:

$$\frac{N_{L0,G0}}{N_{S,G0}} \overset{(28),(30)}{=} \frac{\overline{N}_{L0,G0}}{\overline{N}_{S,G0}} \overset{(33),(34)}{=} \frac{(1-P_{sym})}{(1+P_{sym})} \tag{35}$$

By substituting in Eq (23) the populations of stem proliferating, LIMP proliferating and LIMP dormant cells from Eqs (27), (29) and (30), and by taking into consideration Eq (7) in Kolokotroni *et al.* (2011) we get:

$$\overline{N}_{L0,P} = \frac{(1-P_{sym})}{(1+P_{sym})}\overline{N}_{S,P} \tag{36}$$

For the ratio between the LIMP proliferating cells of *n*=0 class and the stem proliferating cells we get:

$$\frac{N_{L0,P}(t)}{N_{S,P}(t)} \overset{(31),(32)}{=} \frac{\overline{N}_{L0,P}}{\overline{N}_{S,P}} \overset{(36)}{=} \frac{1-P_{sym}}{1+P_{sym}} \tag{37}$$

Therefore, the ratio between the LIMP cells of *n*=0 class and the stem cells is:

$$\frac{N_{L0}(t)}{N_S(t)} = \frac{N_{L0,P}(t)+N_{L0,G0}(t)}{N_{S,P}(t)+N_{S,G0}(t)} \overset{(35),(37)}{=} \frac{\frac{(1-P_{sym})}{(1+P_{sym})}N_{S,P}(t)+\frac{(1-P_{sym})}{(1+P_{sym})}N_{S,G0}(t)}{N_{S,P}(t)+N_{S,G0}(t)} \Rightarrow$$

$$\frac{N_{L0}(t)}{N_S(t)} = \frac{(1-P_{sym})}{(1+P_{sym})}\frac{N_{S,P}(t)+N_{S,G0}(t)}{N_{S,P}(t)+N_{S,G0}(t)} \Rightarrow$$

$$\frac{N_{L0}(t)}{N_S(t)} = \frac{(1-P_{sym})}{(1+P_{sym})} \tag{38}$$

### 5.2 Ratio of LIMP cells n class to n-1 class

In this section we will compute the ratio between the LIMP cell population of class *n* and the one of the preceding class *n*-1:

$$\frac{N_{Ln}(t)}{N_{Ln-1}(t)} = \frac{N_{Ln,P}(t)+N_{Ln,G0}(t)}{N_{Ln-1,P}(t)+N_{Ln-1,G0}(t)}, \quad n=1,\ldots,N-1 \tag{39}$$

By substituting the populations of LIMP proliferating and dormant cells from Eqs (29) and (30) in the differential equation (25) and the boundary condition (26), and by taking into consideration Eq (7) in Kolokotroni *et al.* (2011), it yields:

$$\overline{N}_{Ln,G0} = 2 * \frac{P_{sleep}}{\left(a + R_A + \frac{1}{T_{G0}}\right)} e^{-(a+R_A)T_C} \overline{N}_{Ln-1,P} \tag{40}$$

$$\overline{N}_{Ln,P} = \frac{2}{1 + P_{sym}} \overline{N}_{Ln-1,P} \tag{41}$$

Based on the above Eqs (40) and (41) and Eqs (29), (30), the ratio between the LIMP proliferating or dormant cells of n class and the LIMP proliferating of n-1 class can be derived:

$$N_{Ln,P} = \frac{2}{1 + P_{sym}} N_{Ln-1,P} \tag{42}$$

$$N_{Ln,G0} = 2 * \frac{P_{sleep}}{\left(a + R_A + \frac{1}{T_{G0}}\right)} N_{Ln-1,P} \tag{43}$$

By substituting Eqs (42) and (43) in Eq (39) and after performing some derivations the requested ratio can be computed:

$$\frac{N_{Ln}(t)}{N_{Ln-1}(t)} = \frac{2}{1 + P_{sym}}, \; n = 1, \ldots, N - 1 \tag{44}$$

### 5.3 Ratio of LIMP cells n class to stem cells

By taking into consideration Eqs (38) and (44), the ratio between the LIMP cell population of *n*=1 class and the stem cell population can be derived:

$$\frac{N_{L1}(t)}{N_S(t)} = \frac{N_{L1}(t)}{N_{L0}(t)} \frac{N_{L0}(t)}{N_S(t)} = \frac{2}{(1+P_{sym})} \frac{(1-P_{sym})}{(1+P_{sym})} \Rightarrow$$

$$\frac{N_{L1}(t)}{N_S(t)} = 2 \frac{(1-P_{sym})}{(1+P_{sym})^2}$$

The ratio between the LIMP cell population of *n*=2 class and the stem cell population is:

$$\frac{N_{L2}(t)}{N_S(t)} = \frac{N_{L2}(t)}{N_{L1}(t)} \frac{N_{L1}(t)}{N_S(t)} = \frac{2}{(1+P_{sym})} 2 \frac{(1-P_{sym})}{(1+P_{sym})^2} \Rightarrow$$

$$\frac{N_{L2}(t)}{N_S(t)} = 2^2 \frac{(1-P_{sym})}{(1+P_{sym})^3}$$

Based on induction hypothesis and by taking into consideration Eq (38), the ratio between LIMP cells of class $n$ and stem cells can be derived:

$$\frac{N_{Ln}(t)}{N_S(t)} = 2^n \frac{(1-P_{sym})}{(1+P_{sym})^{n+1}}, \quad n = 0,\ldots,N \tag{45}$$

### 5.4 Ratio of stem to LIMP cells

Based on Eq (45) the ratio between stem cells and the total population of LIMP cells can be computed:

$$\frac{N_S(t)}{N_L(t)} = \frac{N_S(t)}{\sum_{n=0}^{N-1} N_{Ln}(t)} = \frac{1}{\sum_{n=0}^{N-1} \frac{N_{Ln}(t)}{N_S(t)}} = \frac{1}{\sum_{n=0}^{N-1} 2^n \frac{(1-P_{sym})}{(1+P_{sym})^{n+1}}} \Rightarrow$$

$$\frac{N_S(t)}{N_L(t)} = \frac{1}{(1-P_{sym})\sum_{n=0}^{N-1} \frac{2^n}{(1+P_{sym})^{n+1}}} \tag{46}$$

## 6. Growth fraction

The growth fraction (GF) is defined in the present paper as the ratio between the proliferating cells and the total living population, that is:

$$GF = \frac{N_P(t)}{N_P(t)+N_{G0}(t)+N_{Diff}(t)} = \frac{1}{1+\frac{N_{G0}(t)}{N_P(t)}+\frac{N_{Diff}(t)}{N_P(t)}}$$

Taking into account Eqs (5) and (9), it yields:

$$GF = \frac{1}{1+(1+P_{sym})P_{sleep}\frac{a+R_A}{\left(a+R_A+\frac{1}{T_{G0}}\right)}\frac{1}{\left(e^{(a+R_A)Tc}-1\right)}+(1-P_{sym})\frac{\alpha+R_A}{\left(\alpha+R_{A,Diff}+R_{N,Diff}\right)}\frac{1}{\left(e^{(\alpha+R_A)T_C}-1\right)}} \Rightarrow$$

$$GF = \frac{1}{1+\frac{a+R_A}{\left(e^{(a+R_A)Tc}-1\right)}\left(\frac{(1+P_{sym})P_{sleep}}{\left(a+R_A+\frac{1}{T_{G0}}\right)}+\frac{(1-P_{sym})}{\left(\alpha+R_{A,Diff}+R_{N,Diff}\right)}\right)} \tag{47}$$

## 7. Comparison of theoretical analysis with simulation

The following Table lists, for indicative values of the input parameters, the theoretical values of the fractions of the various cell populations, as derived from the above equations and the computational ones as obtained by performing the corresponding simulation runs using the model. As explained in Kolokotroni *et al.* (2011) the deviation between the theoretical and computational values is higher as more dormant cells re-enter cell cycle, (i.e. for higher $P_{G0toG1}$ and higher proportion of dormant cells). However, based on the set of parameters examined, it remains low (0-4%) for cell populations with a proportion of the order of $10^{-2}$ and higher. We conclude that the theoretical analysis predicts with satisfactory accuracy the initial cellular composition of the simulated tumor.

**Table A. Comparison of model simulation results with compartmental analysis in respect to cell composition.**

| | Simulation A | | Simulation B | |
|---|---|---|---|---|
| | Theoretical value* | Computational value* | Theoretical value* | Computational value* |
| $N_{G0}/Total$ | 0.0920 | 0.0920 | 0.2453 | 0.2457 |
| $N_{Diff}/Total$ | 0.7774 | 0.7781 | 0.5471 | 0.5499 |
| $N_A/Total$ | 0.0058 | 0.0048 | 0.0168 | 0.0152 |
| $N_N/Total$ | 0.0343 | 0.0338 | 0.0230 | 0.0220 |
| $N_S/N_L$ | 0.0897 | 0.0897 | 0.000095** | 0.000095** |
| *Growth fraction* | 0.0943 | 0.0946 | 0.1748 | 0.1737 |

| | A | B |
|---|---|---|
| $T_c$ (h) | 30 | 50 |
| $T_{G0}$ (h) | 96 | 334 |
| $T_N$ (h) | 20 | 15 |
| $T_A$ (h) | 6 | 10 |
| $N_{LIMP}$ | 7 | 17 |
| $R_A$ ($h^{-1}$) | 0.001 | 0.0001 |
| $R_{ADiff}$ ($h^{-1}$) | 0.001 | 0.003 |
| $R_{NDiff}$ ($h^{-1}$) | 0.001 | 0.002 |
| $P_{G0toG1}$ | 0.01 | 0.4 |
| $P_{sleep}$ | 0.26 | 0.20395 |
| $P_{sym}$ | 0.4 | 0.16 |

* Values rounded to forth decimal place
** Value rounded to two significant figures

## References

Bertuzzi A, Gandolfi A, Sinisgalli C, Iacoviello D. Steel's potential doubling time and its estimation in cell populations affected by nonuniform cell loss. Math Biosci. 1997;143:61–89.

Kolokotroni EA, Dionysiou DD, Uzunoglu NK, Stamatakos GS. Studying the growth kinetics of untreated clinical tumors by using an advanced discrete simulation model. Math Comput Model. 2011;54:1989-2006. doi:10.1016/j.mcm.2011.05.007

Stamatakos GS, Kolokotroni EA, Dionysiou DD, Georgiadi ECh, Desmedt C. An advanced discrete state – discrete event multiscale simulation model of the response of a solid tumor to chemotherapy. Mimicking a clinical study. J Theor Biol. 2010;266(1):124-39. doi: 10.1016/j.jtbi.2010.05.019
