# Supplementary material for: In Silico Oncology: Quantification of the In Vivo Antitumor Efficacy of Cisplatin-Based Doublet Therapy in Non-Small Cell Lung Cancer (NSCLC) through a Multiscale Mechanistic Model
Source: PLoS Comput Biol. 2016 Sep 22;12(9):e1005093. doi: 10.1371/journal.pcbi.1005093 (PMC5033576; doi:10.1371/journal.pcbi.1005093)
Supplement: S3 Text — (PDF) [file pcbi.1005093.s003.pdf]

Eleni Kolokotroni, Dimitra Dionysiou, Christian Veith, Yoo-Jin Kim, Jörg Sabczynski, Astrid Franz, Aleksandar Grgic, Jan Palm, Rainer M. Bohle, Georgios Stamatakos

S3 Text

Literature Survey: Proliferation features of non-small cell lung cancer (NSCLC)

A comprehensive literature survey on non-small cell lung cancer (NSCLC), has been performed, in order to define biologically-reasonable and cancer-specific value ranges for critical cancer cell proliferation features. The results will guide the selection of model parameter values for the sensitivity analysis and the cell kill rate (CKR) estimation of the real clinical cases.

1. Literature survey methodology

A literature survey on the growth kinetics of primary NSCLC has been performed using the 'Google Scholar' search engine and the National Library of Medicine Medline database (http://www.ncbi.nlm.nih.gov/pubmed/). The survey has been primarily focused on studies attempting to assess the tumor volume doubling time, T_d, based on volumetric methods, the Ki-67 index (a marker of tumor growth fraction), hypoxia, the apoptotic index and the extend of tumor necrosis. Articles correlating tumor kinetics with histological subtype and articles with a considerable number of examined clinical cases have been preferred. No restriction on publication date and study methodology has been imposed. Relevant references in the reviewed papers have also been taken into account.

The literature has also been reviewed on the growth characteristics of established cell lines derived from patients with NSCLC. Histological types of interest have been the squamous cell carcinoma, the adenocarcinoma and the mixed squamous and adenocarcinoma type, regardless of tumor source (lung, pleural effusion, lymph-node metastasis, etc.)

Moreover, a literature search on lung cancer stem cells has been conducted. The search was guided by review papers in the relevant field. Studies attempting to isolate and evaluate cancer stem cells from both cell lines and primary tumors have been taken into account.

The survey performed is not meant to be an exhaustive review of the literature but only to serve as a biologically-relevant and cancer-specific basis for the analysis performed in the present paper.

2. NSCLC proliferation features

2.1 Growth rate

Table A summarizes some of the largest studies on the growth rate of primary NSCLC found in literature [1-18]. Estimates of tumor volume doubling time, T_d, are typically based on 2 or more tumor examinations obtained prior to treatment/surgical resection. An exponential model of tumor growth between the examinations has been consistently assumed. Several methodologies have been applied, differing in the imaging technique and the adopted formalism for the calculation of tumor volume. Early approaches assume spherical or ellipsoidal tumor shapes while growth rates are assessed based on tumor diameter measurements obtained from chest radiographs or CT scans. More sophisticated approaches utilize CT images of thin-slice thickness and three-dimensional volumetric software allowing a more accurate estimation of tumor volume and, hence, growth rate.

Table A. Tumor volume doubling times in days for NSCLC according to histological type.

Study	Number of cases	Mean ±σ	Median	Range	Modality/ Methodology for volume estimation
		Adenocarcinoma			
Spratt *et al.*, 1963 [1]	7	269	-	*Min:*32	Radiograph/ tumor shadow diameter
Steel, 1977 [2]	64	148	-	-	-
Geddes, 1979 [3]	60	161	-		Radiograph
Kerr and Lamb, 1984 [4]	4	72	-	23-110	Radiograph/ tumor shadow diameter
Usuda *et al.*,1994 [5]	86	223.1±209.4	-		Radiograph/ max and perpendicular dimensions
Hasegawa *et al.*, 2000 [6]	49	533	-		CT scan/ max and perpendicular dimensions
Winner-Muram *et al.*, 2002 [7]	15 Stage I	-	157	64-(-26711)	CT scan/ sum of cross-sectional areas
Jennings *et al.*, 2006 [8]	51	166‡	216‡	32-(-52)‡	CT scan/ sum of cross-sectional areas
Lindell *et al.*, 2007 [9]	22 non-BAC	746	343		CT scan/ longest horizontal and max perpendicular diameter
	9 BAC	780	210		
Quint *et al.*, 2008 [10]	15	127	181	58-2239	CT scan/ volumetric software
Honda *et al.*, 2009 [11]	40	248*	334*†	69 -18678, 5 cases negative	CT scan/ volumetric software
Dhopeshwarkar *et al.*, 2011 [12]	21	292	227		CT scan/ longest horizontal axis and the maximum diameter perpendicular to it.
Henschke *et al.*, 2012 [13]	43 solid	-	140	-	CT scan/ the average of length and width on the largest cross-sectional area
	11 subsolid		251		
Kanashiki *et al.*, 2012 [14]	140	177	134		Radiograph
Murai *et al.*, 2012 [15]	135 Stage I		170		CT scan/ max and perpendicular dimensions

Heuvelmans *et al.*, 2013 [16]	15		196		CT scan/ volumetric software
Mackintosh *et al.*, 2014 [17]	36	-	294‡	54-2256, 3 cases negative	CT scan/ volumetric software
Nakamura *et al.*, 2014 [18]	69		305		CT scan
Squamous cell carcinoma					
Spratt *et al.*, 1963 [1]	6	93	-	14-(-325)	Radiograph/ based on diameter of tumor shadow assuming spherical shape
Steel, 1977 [2]	85	85	-		
Geddes, 1979 [3]	111	88	-		Radiograph
Kerr and Lamb, 1984 [4]	7	146	-	20-382	
Usuda *et al.*,1994 [5]	67	104.7±105.6	-		Radiograph
Hasegawa *et al.*, 2000 [6]	8	129±97	-		CT scan
Winner-Muram *et al.*, 2002 [7]	16	-	119	33-1004	
Jennings *et al.*, 2006 [8]	48	132‡	142‡	26-(-50)‡	
Lindell *et al.*, 2007 [9]	8	103	88		CT scan
Quint *et al.*, 2008 [10]	11	128	139	79-901	
Honda *et al.*, 2009 [11]	11	126±58	131	39-221	CT scan
Dhopeshwarkar *et al.,* 2011 [12]	4	73	63		
Henschke *et al.,* 2012 [13]	21		88		CT scan/ based on the average of length and width on the largest cross-sectional area of the cancerous nodule
Kanashiki *et al.,* 2012 [14]	44	133	99		Radiograph
Murai *et al.*, 2012 [15]	66		93		CT scan, based on longest diameter and the diameter perpendicular to it
Heuvelmans *et al.*, 2013 [16]	6		142		
Mackintosh *et al.*, 2014 [17]	6	-	97	66-255	CT scan, semi-automated volumetric software
Nakamura *et al.*, 2014 [18]			81		CT scan

*Values re-calculated here after a meta-analysis of the published data, as the reciprocal of the mean and median growth rate, *k*, i.e. $mean\ T_d = \frac{\ln 2}{\frac{\sum_{i=1}^{N} k_i}{N}}, median\ T_d = \frac{\ln 2}{median k_i}$ where $k = \frac{\ln 2}{T_d}$.

†Negative values of T_d are included.

‡As derived from the reciprocal of the mean or median growth rate reported in the study.

The review reveals a very wide range of tumor T_d spanning from ~20 days to negative values. Regardless of method, the vast majority of studies have reported shorter T_d for SCC than for ADC. Mean and median values for SCC are usually below 140 days, whereas slowly growing or stable tumors (with T_d more than a year or negative) are more frequently reported for the ADC type. Notably, for cases with three or more tumor observations, the growth rate appears fairly constant on the log scale over the observation period [3, 10, 19]. In terms of tumor grade, undifferentiated or poorly differentiated tumors seem to grow at a faster pace than well-differentiated tumors [17].

2.2 Growth fraction.

Ki-67 is a nuclear protein, expressed in proliferating cells during G1, S, G2 and mitosis phases [20]. Despite scarce evidence for the existence of minor amounts in G0 cells [21], it is considered absent during quiescent state and, occasionally, in early G1 cells [20]. Due to its presence in all phases of the active cell cycle only, Ki-67 is widely accepted as a cellular marker of proliferation [22, 23] and, hence, an indicator of the proportion of living cells in a tumor population that are actively proliferating (termed growth fraction).

In the vast majority of the reviewed studies [24-40] Ki-67 values derive from specimens of surgically resected tumors, without pre-operative systemic therapy or radiotherapy. A wide range of Ki-67 values has been observed for NSCLC, approximately from 0 up to 90%, whereas Ki-67 index has consistently been reported to be lower in adenocarcinoma than in squamous cell carcinoma (Table B). Regarding prognosis, Ki-67 labeling index seems to be of significance in NSCLC with a high value indicating poor prognosis.

Table B. Ki-67(%) labeling index for NSCLC.

Study	Stage	No of patients	Mean ±σ	Median	Range	Sample origin/ Method
Soomro & Whimster, 1990 [24]	-	30 ADC	10.99±11.09	7.45	0-47.5	Immunocytochemistry, Ki-67 antibody
		36 SCC	15.71±12.59	13.8	0-49.2	
Hayashi *et al.*, 1993 [25]	-	34 ADC	15.2 ± 1.8		0-50‡	resected tumor/ Ki-67 antibody
		19 SCC	29.5±3.5		10-70‡	
O'Neill *et al.* 1996 [26]	T1, T2	22 ADC	21		5.2-56.2	resected tumor/ IHC, Ki-67 antibody
		54 SCC	31.8		8.9-72	
Rudolph *et al.*, 1998 [27]	-	23	38.3±17.8			resected tumor/ IHC, Ki-S5 antibody
Hommura *et al.*, 2000 [28]	(p) I-IV	215 (106 ADC, 91 SCC, 10 Large, 8 ADSCC)	36.7 ± 27.3		0–93	resected tumor*/ IHC, MIB-1 antibody
Cagini *et al.*, 2000 [29]	I, II	99 (28 ADC, 44 SCC, 22 Large, 5 Bronchioloalveolar)	25.3±19.3	20		resected tumor*/ IHC, MIB-1 antibody
Demarchi *et al.*, 2000 [30]	I-III	64 ADC	21.6±16.8	22.22	0-67	resected tumor*/ IHC, MIB-1 antibody

van de Vaart *et al.* 2000 [31]	III	27 (6 ADC, 11 SCC, 9 Large, 1 Undifferentiated)	59.6±19.3	60.3	22-94.3	biopsy*/ IHC, MIB1 antibody
Rigau *et al.*, 2002 [32]	I-IV	86 (32 ADC, 42 SCC, 12 Large)		37		resected tumor*/ IHC, MIB-1 antibody
Tsoli *et al.*, 2001 [33]	I-III	69 (33 ADC, 31 SCC, 5 UCL)	34.8±11.29	33.1	4.6 - 70.4	resected tumor*/ IHC, MIB-1 antibody
		33 ADC	29.4±10.1	29.2		
		31 SCC	40.14±10.4	39.65		
Ferreira *et al.*, 2001 [34]	I-IIIA	144 (40 ADC, 74 SCC, 30 LCC)	49.3±25.1			resected tumor/ IHC, MIB-1 antibody
Takahashi *et al.*, 2002 [35]	pT1-pT4	62 (36 ADC, 26 SCC)	24.5			resected tumor*/ IHC, MIB-1 antibody
Haga *et al.* 2003 [36]	I	187	19.3			resected tumor/ IHC, MIB-1 antibody
		122 ADC	10.7			
		65 SCC	35.4			
Tsubochi *et al.* 2006 [37]	I-III	219 (116 ADC, 97 SCC, 5 ADSCC, 1 Large)	18.9			resected tumor*/ IHC, Ki-67 antibody
		116 ADC	12.0			
		97 SCC	26.1			
Kaira *et al.*, 2008 [38]	I-III	321 (200 ADC, 100 SCC, 21 Large)	35±24	32	5-92	resected tumor*/ IHC, MIB-1 antibody
		200 ADC	23±20	18		
		100 SCC	54±17	56		
Warth *et al.* 2014 [39]		1065	40.7			IHC
		184 ADC	25.8			
		233 SCC	52.8			
Chen *et al.* 2014 [40]	I-IV	191 (93 ADC, 98 SCC)		31		resected tumor*/ IHC, Ki-67 antibody

SCC: Squamous cell carcinoma, ADC: Adenocarcinoma, ADSCC: mixed squamous and adenocarcinoma of the lung, NSCLC: Non-small cell lung cancer, IHC: immunohistochemistry

*Explicitly stated that neither chemotherapy nor radiotherapy was performed before surgery or biopsy

‡Approximately determined from relevant graph in the study

2.3 Cell cycle time

In the present study, the average cell cycle duration of human lung tumors, *in vivo,* is assumed to be reflected in the mean generation time (or population doubling time) of lung cancer cell lines. Cell lines grow *in vitro* (e.g. as an adherent monolayer or floating cultures) and the generation time is typically determined from the slope of the growth curve during exponential growth phase. The mean generation time of a cell population is comparable to its average cell cycle time when the vast majority of cells in the population are proliferative, cell loss is low, and cells are characterized

by fairly similar cell cycle kinetics [41-43]. The above conditions are generally met in established, immortal cell lines under typical culture conditions [42].

In the reviewed literature [44-53], population doubling times of established cell lines derived from patients with adenocarcinoma or squamous cell carcinoma range from 18 to 134 h (Table C). No significant statistical difference in generation time has been reported for the two aforementioned histological types.

Table C. Population doubling time (in hours) for human NSCLC established cell lines.

Study	No of cell lines	Range (Median)	Medium
Lieber *et al.* 1976 [44]	1 ADC	48	DMEM plus 10% FBS
Kimura *et al.* 1979 [45]	1 SCC	46	α-MEM plus 10% FBS
Loh *et al.* 1984 [46]	3 ADC, 1 ADSCC	34.5, 36.1, 55.8, 102.8	enriched CMRL-1066 plus 10% FBS
Olsson *et al.* 1984 [47]	1 SCC	~ 30 to 40	RPMI-1640 plus FBS
Brower *et al.* 1986 [48]	7 ADC, 1 SCC	18-58 (38.5)	RPMI 1640 plus 10% FBS
Masuda *et al.* 1991 [49]	15 ADC, 1 SCC	21-65.5 (38.65)	RPMI 1640 plus 10% FBS
Campling *et al.* 1992 [50]	1 ADC	41.4	HITES plus 2.5% FBS
Liu & Tsao, 1993 [51]	4 ADC, 1 SCC, 1ADSCC	53-134 (79.5)	R-10 or ACL-4
Giaccone *et al.* 1992 [52]	2 ADC	24, 72	RPMI 1640 plus 10% FBS
Li *et al.* 2012 [53]	1 ADC, 2SCC	25, 38, 42	DMEM plus 10%FBS

SCC: Squamous cell carcinoma, ADC: Adenocarcinoma, NSCLC: Non-small cell lung cancer, ADSCC: mixed squamous and adenocarcinoma of the lung, MEM: minimum essential medium, DMEM: Dulbecco's modified Eagle's medium, FBS: fetal bovine serum

2.4 Apoptosis and Necrosis

For solid tumors, apoptosis and necrosis are two morphologically distinct modes of cell death that may both occur in a tumor at the same time. In normal tissues, spontaneous apoptosis is a fundamental homeostatic mechanism for the preservation of a constant cell population. Regarding cancer cells, their ability to escape apoptosis favors the malignancy outgrowth. Several publications have attempted to quantify the presence of apoptosis in tumor tissue specimens at diagnosis and correlate it with treatment prognosis [26, 31, 33, 34, 54-61]. Consistent with most solid tumor types [62], the frequency of apoptosis in NSCLC appears to be very low (Table D), most likely due to the rapid formation and phagocytic clearance of apoptotic debris *in vivo* [63-65]. In the reviewed studies, the mean apoptotic index (AI - expressed as the number of apoptotic cells and/or bodies per 100 malignant cells) spans from 0 to 12%, with a mean and median value ranging between 0.3-4.3 and 0.8–4, respectively. Higher levels of apoptosis have been reported in literature for SCC than ADC; however, the difference is not characterized as statistically significant [60]. Apoptotic cells are evenly distributed throughout a tumor, without preferential sites of accumulation [54]. Regarding the prognostic significance of AI, published data point toward contradictory conclusions.

Table D. Apoptotic Index (number of apoptotic cells and apoptotic bodies per 100 tumor cells) for NSCLC.

Study	Stage/ Classification	No of patients	Mean ±σ	Median	Range	Sample origin/ Method
Törmänen *et al.* 1995 [54]	$T_{1\text{-}3}N_{0\text{-}2}$	24 ADC<br>47 SCC	1.24±0.24‡<br>1.20±0.19‡			resected tumor/ TUNEL
O'Neill *et al.* 1996 [26]	$T_{1\text{-}2}$	22 ADC<br>54 SCC	1.1<br>2.3		0.1-5.4<br>0.1-9.6	resected tumor/ H&E staining
Komaki *et al.* 1996 [55]	N_1	173 (73 ADC, 86 SCC, 3 LCC, 6 ADSCC, 8 Others)		1.0	0.2-2.8	resected tumor*/ H&E staining
Stammler & Volm, 1996 [56]	I-III	178	0.37±0.29‡	0.25‡	0-1.7‡	resected tumor*/ TUNEL
Tanaka *et al.* 1999 [57]	I-IIIa	236 (130 ADC, 85 SCC, 13 LCC, 8 Others)<br>130 ADC<br>85 SCC	1.88±0.14<br>1.97±0.23<br>1.96±0.19	1.1		resected tumor*/ TUNEL
van de Vaart et al. 2000 [31]	III	27	1.8	2	0-8	biopsy*/ TUNEL assay
Langendijk et al. 2000 [58]	III	75	0.9		0-10	bronchoscopy*
Tsoli *et al.*, 2001** [33]	I-III	60 (29 ADC, 28 SCC, 3 ULC)<br>29 ADC<br>28 SCC	2 ±1.97<br>2.35±2.5<br>1.46±1.09	1.35<br>1.4<br>1.15	0.1-10.6	resected tumor*/ TUNEL assay
Ferreira *et al.*, 2001 [34]	I-IIIA	144 (40 ADC, 74 SCC, 30 LCC)	0.65±0.41			resected tumor/ H&E staining
Ghosh *et al.*, 2001 [59]		134 SCC	0.3029±0.24 75	0.223	0.024-1.455	resected tumor/ H&E staining
Hwang *et al.*, 2001 [60]	I-III	68 (6 ADC, 62 SCC)<br>6 ADC<br>62 SCC	4.3±2.6<br>5.9±3.54<br>4.1±2.5	4	0.2-12	biopsy*/H&E staining
Dworakowska *et al.*, 2009 [61]	I-IV	170 (43 ADC, 101 SCC, 14 LCC, 12 ADSCC)	1.2±1.0 (of 168 patients), 2 patients had 0%	0.8 (of 168 patients)		resected tumor/ TUNEL, H&E staining

SCC: Squamous cell carcinoma, ADC: Adenocarcinoma, NSCLC: Non-small cell lung cancer, ULC: undifferentiated lung cancer, ADSCC: mixed squamous and adenocarcinoma of the lung
large cell carcinomas, H&E: haematoxylin and eosin
TUNEL: terminal deoxynucleotidyl transferase-mediated dUTP nick end-labeling
‡ Explicitly stated that apoptotic index is expressed per 100 viable tumor cells
*Explicitly stated that no treatment was performed before surgery or biopsy

In contrast, necrosis, either at macroscopic or microscopic level, is more frequently observed in NSCLC (Table E) [54, 66-69] and in solid tumors, in general. During tumor growth, cell necrosis is believed to be the result of oxygen supply deprivation (hypoxia) caused by an insufficient tumor vascular network. A positive correlation of necrosis extent with increased tumor size [70, 71], high-grade (poorly differentiated) disease and poor prognosis (especially for stage I and II disease) has been demonstrated [72, 73]. The above findings suggest that extensive tumor necrosis reflects a fast growing tumor that outstrips its blood supply and, hence, is associated with a more aggressive tumor phenotype [72, 73]. Necrosis appears more often in SCC than in ADC (Table E) indicating the more aggressive nature of the former type [54, 71].

Table E. Percent (%) of tumor necrosis for NSCLC.

Study	Stage	No of patients	Mean ±σ	Median	Distribution	Sample origin/Method
Kessler *et al.* 1996 [66]	I-III	593 (124 ADC, 394 SCC, 46 Large, 29 Bronchoalveolar)	15.7 ± 0.9	5		resected tumor*/ H&E staining
Pataer *et al.*, 2012‡ [67]		192			11–30% (6); 31–50% (27); 51–70% (64); 71–100% (69)	resected tumor*/ H&E staining
Khan *et al.*, 2004 [68]		98 (26 ADC, 70 SCC, 1 Undifferentiated, 1 Bronchoalveolar)			<1/3 (87); >1/3 (11)	-
Törmänen *et al.* 1995 [54]	I-IIIa	24 ADC			0% (17); 1-20% (2); 21-50% (2); 51-70% (2); >71% (1)	resected tumor
		47 SCC			0% (3); 1-20% (24); 21-50% (13); 51-70% (6); >71% (1)	
Lee *et al.* 1989 [69]	III	30 (8 ADC, 18 SCC, 4 LCC)			≤25%: 26; ≥26%:4	Biopsy or resected tumor*/ H&E staining

SCC: Squamous cell carcinoma, ADC: Adenocarcinoma, NSCLC: Non-small cell lung cancer, H&E: haematoxylin and eosin

*Explicitly stated that neither chemotherapy nor radiotherapy was performed before surgery or biopsy

‡The percentages refer to viable tumor

2.5 Cellular Quiescence-Hypoxia

It can be easily observed (see Table B) that in most tumors the majority of cells are non-dividing. Three major types of non-dividing cells can be distinguished, namely terminally differentiated cells with no mitotic capacity, cells out of the cell cycle due to hypoxia and nutrient deficiency, and cells out of the cell cycle due to lack of growth promoting signals. In the last two cases cells retain their capacity to proliferate and cellular quiescence is reversible under appropriate conditions/stimuli.

The literature search did not reveal any study attempting to distinguish or quantify these three types of non-dividing cells in NSCLC. On the other hand, attempts to quantify the extent of hypoxia in NSCLC have been documented in literature. More specifically, in 21 NSCLCs studied with [^{18}F] FMISO PET imaging, the fractional hypoxic volume (FHV), ranged between 1.3 and 94.7% (median 47.6%), when a tumor:blood [^{18}F] activity ratio ≥ 1.4 was taken as a cutoff for hypoxic voxels [74]. Similar results have been reported in [75], where the FHV of 1.4 in 11 stage III or stage IV NSCLC ranged between 5 and 91% (median 48%). The response and viability of hypoxic cells depends on tumor microenvironment, energy levels and the ability of the cell to adapt [76]. Hypoxic cells have been reported to live for a few (4-10) days [77]. However, it has been hypothesized that cells under nutrient deficiency may stay viable in a quiescent state for even longer periods of time, after shrinking through autophagy [78].

2.6 Cancer stem cells

The cancer stem cell (CSC) hypothesis has been proposed to explain the intratumor population heterogeneity, in terms of multipontency, long-term proliferation and tumorigenic potential. Regardless of the maturation level of the cell of origin, CSCs is a subpopulation of cancer cells that has acquired features similar to normal somatic stem cells and is thought to be responsible for tumor initiation, maintenance and propagation. Unlimited mitotic capacity, self-renewal and ability to differentiate and produce the heterogeneous cell lineages that make up a tumor are fundamental characteristics attributed to CSCs.

Criteria used for the isolation of CSC enriched tumor cell populations include increased ALDH activity, low Hoechst 33342 dye staining ('side population'), sphere formation in serum free conditions, expression of stem cell markers (e.g. CD133) and chemo-resistance [79]. Other markers of stemness associated with either adenocarcinoma and/or SCC are CD24, CD44, Oct-4, nestin, SOX2 and CD166 [79-81]; however, a universal marker or combination of markers for the accurate identification of pure CSCs populations are lacking [79, 80].

It has been initially thought that CSCs is a rare population with a frequency well below 0.1% of the total tumor cells (0.1–0.0001%)[82]. In the majority of the reviewed publications [83-88], the reported frequencies for lung cancer ADC and SCC, either in primary tumors or established cell lines, is well below 0.01%. The method used is based on the tumor formation incidence following inoculation of varied quantities of tumor cells in immunocompromised mice. However, over the last years, this hypothesis has been strongly debated based on the findings of [89] and [82]. The high proportion of CSCs in lymphoma, AML and melanoma, has led some investigators to argue that the observed low frequencies of CSCs is the result of microenvironment limitations of the immunocompromised mice used for xenotransplantation and the short observation window (<20 weeks)[90]. Ishizawa *et al.* [85] compared the tumorigenic capacity using both typical and highly permissive xenotransplantation conditions, the latter based on NSG mice. Although elevated, the study failed to demonstrate high proportions of CSCs using highly permissive xenotransplantation conditions, in all of the primary tumors examined. More specifically, the estimated frequency of CSCs was of the order of 10^{-5} in non-small cell lung adenocarcinoma and 10^{-5}-10^{-4} in squamous cell carcinoma (Table F).

CSC theory has also been used to explain resistance to chemotherapy and recurrence. Drug surviving cells following *in vitro* exposure to conventional chemotherapeutic drugs (e.g. cisplatin, docetaxel etc.), exhibit elevated levels of stem-related markers, as well as, all typical stemcellness properties [84]. Likewise, drug resistance is utilized as one of the experimental validation checks for CSC enriched populations [79]. The ability of CSCs to escape therapy has been attributed to a quiescent, slow cycling nature [87], more efficient DNA repair and drug efflux mechanisms, and

an elevated metabolic activity in respect to bulk tumor cells [91]. In the reviewed publications [83, 92] the resistance of CSC enriched populations seems to range considerably depending on drug, dosage, method of CSC isolation and the original sample itself (Table F).

Table F. CSCs in NSCLC.

Study	Cell line/ Primary Sample	Value	Methods
		**Frequency (% of total alive cells)**	
Ho *et al.*, 2007 [83]	H460 cell line	0.00041*†	Marker: SP, NOD/SCID mice sacrificed at day 60 or when tumor measured ~1000 mm^3
	H441 cell line	0.0054*†	
	A549 cell line	0.0044*†	
Levina *et al.*, 2008 [84]	H460 cell line	0.0026*	SCID mice sacrificed when tumor measured ~2cm in diameter
Ishizawa *et al.*, 2010 [85]	3 ADC patients	0.0033-0.0.0063	NSG mice monitored for at least 20 weeks
		0.0023,<0.001	NOD/SCID mice monitored for at least 20 weeks
	3 SCC patients	0.0063-0.024	NSG mice monitored for at least 20 weeks
		0.0080-0.0023	NOD/SCID mice monitored for at least 20 weeks
Zhang *et al.*, 2012 [86]	H460 cell line	0.00026*	NU/NU mice monitored maximum for 16 weeks
Singh *et al.*, 2013 [87]	H1650 cell line	0.0073*	SCID-beige mice monitored maximum for 19 weeks
Xu *et al.*, 2014 [88]	A549 cell line	0.00019*	NOD/SCID mice sacrificed after 12 weeks or when tumors measured >1 cm in diameter
	H460 cell line	0.00025*	
	SK-MES-1 cell line	0.00022*	
		**Resistance‡**	
Ho *et al.*, 2007 [83]	H460, HTB-58, H441, H2170 cell lines	CIS: 0.74-1.0[a], GEM: 0.65-1.0[a], VINO: 0.61- 1.1[a], DOC:0.77-1.2[a]	SP vs. non-SP, 24h exposure at IC_{50} of unsorted cell lines
Jiang *et al.*, 2009 [92]	H460, H322, H358, H125 cell lines	CIS: 0.67-0.74[a], GEM: 0.56-0.85[a], VINO: 0.27- 0.84[a], DOC:0.40-0.80[a]	ALDH1+ vs. ALDH1, 24h exposure at IC_{50}

SCC: Squamous cell carcinoma, ADC: Adenocarcinoma, NSCLC: Non-small cell lung cancer, CSCs: Cancer stem cells, SP: side population, NSG: NOD/SCID/Gamma, NOD: Non-obese diabetic, SCID: Severe combined immune deficiency, IC_{50}: half maximal inhibitory concentration

All values have been rounded to two significant figures.

*Value calculated, following a meta-analysis of published data based on extreme limiting dilution analysis [93].

†Value calculated as the mean CSC frequency of marker positive and marker negative cells by the formula:

(CSC % in marker+ cells)×(marker+ fraction of total cells)+ (CSC % in marker- cells)×(marker- fraction of total cells)

‡Approximately determined from relevant graph in the study. Expressed as the ratio of the CKR (=1-percentage viability/100) for marker positive cells to the CKR for marker negative cells[a] or parental tumor[b]

References

[1] Spratt JS, Spjut HJ, Roper CL. The frequency distribution of the rates of growth and the estimated duration of primary pulmonary carcinomas. Cancer. 1963;16:687–93.

[2] Steel 1977. In: Steel GG, editor. Basic Clinical Radiobiology. 3rd ed. London: Arnold; 2002. p 11.

[3] Geddes DM. The natural history of lung cancer: a review based on rates of tumour growth. Br J Dis Chest. 1979;73:[33]1–17.

[4] Kerr KM, Lamb D. Actual growth rate and tumour cell proliferation in human pulmonary neoplasms. Br J Cancer. 1984;50(3):343-49.

[5] Usuda K, Saito Y, Sagawa M, Sato M, Kanma K, Takahashi S, et al. Tumor doubling time and prognostic assessment of patients with primary lung cancer. Cancer. 1994;74(8):2239–44.

[6] Hasegawa M, Sone S, Takashima S, Li F, Yang ZG, Maruyama Y, et al. Growth rate of small lung cancers detected on mass CT screening. Br J Radiol. 2000;73:1252–9.

[7] Winer-Muram HT, Jennings SG, Tarver RD, Aisen AM, Tann M, Conces DJ, et al. Volumetric growth rate of stage I lung cancer prior to treatment: serial CT scanning. Radiology. 2002;223:798–805.

[8] Jennings SG, Winer-Muram HT, Tann M Ying J, Dowdeswell I. Distribution of stage I lung cancer growth rates determined with serial volumetric CT measurements. Radiology. 2006;241:554–63.

[9] Lindell RM, Hartman TE, Swensen SJ, Jett JR, Midthun DE, Tazelaar HD, et al. Five-year lung cancer screening experience: CT appearance, growth rate, location, and histologic features of 61 lung cancers. Radiology. 2007;242:555–62.

[10] Quint LE, Cheng J, Schipper M, Chang AC, Kalemkerian G. Lung lesion doubling times: values and variability based on method of volume determination. Clin Radiol. 2008;63(1):41-8.

[11] Honda O, Johkoh T, Sekiguchi J, Tomiyama N, Mihara N, Sumikawa H, et al. Doubling time of lung cancer determined using three-dimensional volumetric software: Comparison of squamous cell carcinoma and adenocarcinoma. Lung Cancer. 2009;66(2):211-7.

[12] Dhopeshwarkar MR, Roberts HC, Paul NS, Dong Z, Tsao M, Menezes RJ. Screen-detected lung cancer: a retrospective analysis of CT appearance. Acad Radiol. 2011;18:1270-6. doi: 10.1016/j.acra.2011.06.005

[13] Henschke CI, Yankelevitz DF, Yip R, Reeves AP, Farooqi A, Xu D, et al; Writing Committee for the I-ELCAP Investigators. Lung cancers diagnosed at annual CT screening: volume doubling times. Radiology. 2012 May;263(2):578-83. doi: 10.1148/radiol.12102489

[14] Kanashiki M, Tomizawa T, Yamaguchi I, Kurishima K, Hizawa N, Ishikawa H, et al. Volume doubling time of lung cancers detected in a chest radiograph mass screening program: Comparison with CT screening. Oncol Lett. 2012 Sep;4(3):513-516.

[15] Murai T, Shibamoto Y, Baba F, Hashizume C, Mori Y, Ayakawa S, et al. Progression of non-small-cell lung cancer during the interval before stereotactic body radiotherapy. Int J Radiat Oncol Biol Phys. 2012 Jan 1;82(1):463-7. doi: 10.1016/j.ijrobp.2010.10.001

[16] Heuvelmans MA, Oudkerk M, de Bock GH, de Koning HJ, Xie X, van Ooijen PM, et al. Optimisation of volume-doubling time cutoff for fast-growing lung nodules in CT lung cancer screening reduces false-positive referrals. Eur Radiol. 2013 Jul;23(7):1836-45. doi: 10.1007/s00330-013-2799-9

[17] Mackintosh JA, Marshall HM, Yang IA, Bowman RV, Fong KM. A retrospective study of volume doubling time in surgically resected non-small cell lung cancer. Respirology. 2014 Jul;19(5):755-62. doi: 10.1111/resp.12311

[18] Nakamura R, Inage Y, Tobita R, Mori K, Numata T, Yanai H, et al. Epidermal growth factor receptor mutations: effect on volume doubling time of non-small-cell lung cancer patients. J Thorac Oncol. 2014 Sep;9(9):1340-4. doi: 10.1097/JTO.0000000000000022

[19] Friberg S, Mattson S. On the Growth Rates of Human Malignant Tumors: Implications for Medical Decision Making. J Surg Oncol. 1997;65:284–97.

[20] Gerdes J, Lemke H, Baisch H, Wacker HH, Schwab U, Stein H, et al. Cell cycle analysis of a cell proliferation-associated human nuclear antigen defined by the monoclonal antibody Ki-67. J Immunol. 1984;133(4):1710–5.

[21] Bullwinkel J, Baron-Lühr B, Lüdemann A, Wohlenberg C, Gerdes J, Scholzen T, et al. Ki-67 protein is associated with ribosomal RNA transcription in quiescent and proliferating cells. J Cell Physiol. 2006;206(3):624–35.

[22] Jakobsen JN, Sørensen JB. Clinical impact of ki-67 labeling index in non-small cell lung cancer. Lung Cancer. 2013;79(1):1-7. doi: 10.1016/j.lungcan.2012.10.008

[23] Lelle RJ, Heidenreich W, Stauch G, Gerdes J. The correlation of growth fractions with histologic grading and lymph node status in human mammary carcinoma. Cancer. 1987;59:83-8.

[24] Soomro IN, Whimster WF. Growth fraction in lung tumours determined by Ki67 immunostaining and comparison with AgNOR scores. J Pathol. 1990 Nov;162(3):217-22.

[25] Hayashi Y, Fukayama M, Koike M, Kaseda S, Ikeda T, Yokoyama T. Cell-cycle analysis detecting endogenous nuclear antigens: comparison with BrdU-in vivo labeling and an application to lung tumors. Acta Pathol Jpn. 1993 Jun;43(6):313-9.

[26] O'Neill AJ, Staunton MJ, Gaffney EF. Apoptosis occurs independently of bcl-2 and p53 over-expression in non-small cell lung carcinoma. Histopathology. 1996 Jul;29(1):45-50.

[27] Rudolph P, Peters J, Lorenz D, Schmidt D, Parwaresch R. Correlation between mitotic and Ki-67 labeling indices in paraffin-embedded carcinoma specimens. Hum Pathol. 1998;29(11):1216-22.

[28] Hommura F, Dosaka-Akita H, Mishina T, Nishi M, Kojima T, Hiroumi H, et al. Prognostic significance of p27KIP1 protein and ki-67 growth fraction in non-small cell lung cancers. Clin Cancer Res. 2000;6(10):4073–81.

[29] Cagini L, Monacelli M, Giustozzi G, Moggi L, Bellezza G, Sidoni A, et al. Biological prognostic factors for early stage completely resected non-small cell lung cancer. J Surg Oncol. 2000 May;74(1):53-60.

[30] Demarchi LM, Reis MM, Palomino SA, Farhat C, Takagaki TY, Beyruti R, et al. Prognostic values of stromal proportion and PCNA, Ki-67, and p53 proteins in patients with resected adenocarcinoma of the lung. Mod Pathol (2000) 13(5):511-20.

[31] van de Vaart PJ, Belderbos J, de Jong D, Sneeuw KC, Majoor D, Bartelink H, et al. DNA-adduct levels as a predictor of outcome for NSCLC patients receiving daily cisplatin and radiotherapy. Int J Cancer. 2000 Mar 20;89(2):160-6.

[32] Rigau V, Molina TJ, Chaffaud C, Huchon G, Audouin J, Chevret S, et al. Blood vessel invasion in resected non small cell lung carcinomas is predictive of metastatic occurrence. Lung Cancer. 2002 Nov;38(2):169-76.

[33] Tsoli E, Gorgoulis VG, Zacharatos P, Kotsinas A, Mariatos G, Kastrinakis NG, et al. Low levels of p27 in association with deregulated p53-pRb protein status enhance tumor proliferation and chromosomal instability in non-small cell lung carcinomas. Mol Med. 2001 Jun;7(6):418-29.

[34] Ferreira CG, van der Valk P, Span SW, Ludwig I, Smit EF, Kruyt FA, et al. Expression of X-linked inhibitor of apoptosis as a novel prognostic marker in radically resected non-small cell lung cancer patients. Clin Cancer Res. 2001 Aug;7(8):2468-74.

[35] Takahashi S, Kamata Y, Tamo W, Koyanagi M, Hatanaka R, Yamada Y, et al. Relationship between postoperative recurrence and expression of cyclin E, p27, and Ki-67 in non-small cell lung cancer without lymph node metastases. Int J Clin Oncol. 2002;7(6):349-55.

[36] Haga Y, Hiroshima K, Iyoda A, Shibuya K, Shimamura F, Iizasa T, et al. Ki-67 expression and prognosis for smokers with resected stage I non-small cell lung cancer. Ann Thorac Surg. 2003;75(6):1727–32; discussion 1732-3.

[37] Tsubochi H, Sato N, Hiyama M, Kaimori M, Endo S, Sohara Y, et al. Combined analysis of cyclooxygenase-2 expression with p53 and Ki-67 in nonsmall cell lung cancer. Ann Thorac Surg 2006;82(4):1198–204.

[38] Kaira K, Oriuchi N, Imai H, Shimizu K, Yanagitani N, Sunaga N, et al. Prognostic significance of L-type amino acid transporter 1 expression in resectable stage I–III nonsmall cell lung cancer. Br J Cancer. 2008;98(4):742–8.

[39] Warth A, Cortis J, Soltermann A, Meister M, Budczies J, Stenzinger A, et al. Tumour cell proliferation (Ki-67) in non-small cell lung cancer: a critical reappraisal of its prognostic role. Br J Cancer. 2014 Sep 9;111(6):1222-9. doi: 10.1038/bjc.2014.402

[40] Chen P, Li WM, Lu Q, Wang J, Yan XL, Zhang ZP, et al. Clinicopathologic features and prognostic implications of NOK/STYK1 protein expression in non-small cell lung cancer. BMC Cancer. 2014 Jun 4;14:402. doi: 10.1186/1471-2407-14-402

[41] Kolokotroni EA, Dionysiou DD, Uzunoglu NK, Stamatakos GS. Studying the growth kinetics of untreated clinical tumors by using an advanced discrete simulation model. Math Comput Model. 2011;54:1989-2006. doi:10.1016/j.mcm.2011.05.007

[42] Merrill GF. Cell synchronization. Methods Cell Biol. 1998;57:229–49.

[43] Barnes JA. Dix DJ, Collins BW, Luft C, Allen JW. Expression of inducible Hsp70 enhances the proliferation of MCF-7 breast cancer cells and protects against the cytotoxic effects of hyperthermia. Cell Stress Chaperones. 2001;6(4):316–25.

[44] Lieber M, Smith B, Szakal A, Nelson-Rees W, Todaro G. A continuous tumor-cell line from a human lung carcinoma Lieber with properties of type II alveolar epithelial cells. Int J Cancer. 1976 Jan 15;17(1):62-70.

[45] Kimura N, Shibuya T, Niho Y, Nakamura H, Matsuo S, Imamura T, et al. Human lung cancer cell line (KSNY) producing colony-stimulating activity which affects both human and mouse marrow cells. Gan. 1979 Dec;70(6):807-10.

[46] Loh PM, Clamon GH, Robinson RA, White ML, Hukku B, Rossi NP, et al. Establishment and characterization of four new human non-small cell lung cancer cell lines. Cancer Res. 1984 Aug;44(8):3561-9.

[47] Olsson L, Sorensen HR, Behnke O. Intratumoral phenotypic diversity of cloned human lung tumor cell lines and consequences for analyses with monoclonal antibodies. Cancer. 1984 Nov 1;54(9):1757-65.

[48] Brower M, Carney DN, Oie HK, Gazdar AF, Minna JD. Growth of cell lines and clinical specimens of human non-small cell lung cancer in a serum-free defined medium. Cancer Res. 1986 Feb;46(2):798-806.

[49] Masuda N, Fukuoka M, Takada M, Kudoh S, Kusunoki Y. Establishment and characterization of 20 human non-small cell lung cancer cell lines in a serum-free defined medium (ACL-4). Chest. 1991 Aug;100(2):429-38.

[50] Campling BG, Haworth AC, Baker HM, Greer DL, Holden JJ, Bradley EC, et al. Establishment and characterization of a panel of human lung cancer cell lines. Cancer. 1992 Apr 15;69(8):2064-74.

[51] Liu C, Tsao MS. Proto-oncogene and growth factor/receptor expression in the establishment of primary human non-small cell lung carcinoma cell lines. Am J Pathol. 1993 Feb;142(2):413-23.

[52] Giaccone G, Gazdar AF, Beck H, Zunino F, Capranico G. Multidrug sensitivity phenotype of human lung cancer cells associated with topoisomerase II expression. Cancer Res. 1992 Apr 1;52(7):1666-74.

[53] Li J, Yang H, Chen L, Li Y, Zhu Y, Dai Y, et al. Establishment and characterization of human non-small cell lung cancer cell lines. Mol Med Rep. 2012 Jan;5(1):114-7. doi: 10.3892/mmr.2011.613

[54] Törmänen U, Eerola AK, Rainio P, Vähäkangas K, Soini Y, Sormunen R, et al. Enhanced apoptosis predicts shortened survival in non-small cell lung carcinoma. Cancer Res. 1995 Dec 1;55(23):5595-602.

[55] Komaki R, Fujii T, Perkins P, Ro JY, Allen PK, Mason KA, et al. Apoptosis and mitosis as prognostic factors in pathologically staged N1 nonsmall cell lung cancer. Int J Radiat Oncol Biol Phys. 1996;36:601-5.

[56] Stammler G, Volm M. Apoptosis in non-small cell lung cancer as related to drug resistance and prognosis. Apoptosis. 1996;1:95-9. doi: 10.1007/BF00142084

[57] Tanaka F, Kawano Y, Li M, Takata T, Miyahara R, Yanagihara K, et al. Prognostic significance of apoptotic index in completely resected non-small-cell lung cancer. J Clin Oncol. 1999 Sep;17(9):2728-36.

[58] Langendijk H, Thunnissen E, Arends JW, de Jong J, ten Velde G, Lamers R, et al. Cell proliferation and apoptosis in stage III inoperable non-small cell lung carcinoma treated by radiotherapy. Radiother Oncol. 2000 Aug;56(2):197-207.

[59] Ghosh M, Crocker J, Morris A. Apoptosis in squamous cell carcinoma of the lung: correlation with survival and clinicopathological features. J Clin Pathol. 2001;54(2):111–5.

[60] Hwang JH, Lim SC, Kim YC, Park KO, Ahn SJ, Chung WK. Apoptosis and bcl-2 expression as predictors of survival in radiation-treated non-small-cell lung cancer. Int J Radiat Oncol Biol Phys. 2001 May 1;50(1):13-8.

[61] Dworakowska D, Jassem E, Jassem J, Karmolinski A, Lapinski M, Tomaszewski D, et al. Prognostic value of the apoptotic index analysed jointly with selected cell cycle regulators and proliferation markers in non-small cell lung cancer. Lung Cancer. 2009;66(1):127-33. doi: 10.1016/j.lungcan.2009.01.008

[62] Proskuryakov SY, Gabai VL. Mechanisms of Tumor Cell Necrosis. Curr Pharm Des. 2010;16:56-68. doi: 10.2174/138161210789941793

[63] Kerr JF, Wyllie AH, Currie AR. Apoptosis: a basic biological phenomenon with wide-ranging implications in tissue kinetics. Br J Cancer. 1972;26(4):239-57.

[64] Gavrieli Y, Sherman Y, Ben-Sasson SA. Identification of programmed cell death in situ via specific labeling of nuclear DNA fragmentation. J Cell Biol. 1992 Nov;119(3):493-501.

[65] Bursch W, Paffe S, Putz B, Barthel G, Schulte-Hermann R. Determination of the length of the histological stages of apoptosis in normal liver and in altered hepatic foci of rats. Carcinogenesis. 1990 May;11(5):847-53.

[66] Kessler R, Gasser B, Massard G, Roeslin N, Meyer P, Wihlm JM, et al. Blood vessel invasion is a major prognostic factor in resected non-small cell lung cancer. Ann Thorac Surg. 1996 Nov;62(5):1489-93.

[67] Pataer A, Kalhor N, Correa AM, Raso MG, Erasmus JJ, Kim ES, et al; University of Texas M. D. Anderson Lung Cancer Collaborative Research Group. Histopathologic response criteria predict survival of patients with resected lung cancer after neoadjuvant chemotherapy. J Thorac Oncol. 2012 May;7(5):825-32. doi: 10.1097/JTO.0b013e318247504a

[68] Khan OA, Fitzgerald JJ, Field ML, Soomro I, Beggs FD, Morgan WE, et al. Histological determinants of survival in completely resected T1-2N1M0 nonsmall cell cancer of the lung. Ann Thorac Surg. 2004 Apr;77(4):1173-8.

[69] Lee TK, Horner RD, Silverman JF, Chen YH, Jenny C, Scarantino CW. Morphometric and morphologic evaluations in stage III non-small cell lung cancers. Prognostic significance of quantitative assessment of infiltrating lymphoid cells. Cancer. 1989 Jan 15;63(2):309-16.

[70] Tantraworasin A, Saeteng S, Lertprasertsuke N, Arreyakajohn N, Kasemsarn C, Patumanond J. Prognostic factors of tumor recurrence in completely resected non-small cell lung cancer. Cancer Manag Res. 2013 Jun 6;5:77-84. doi: 10.2147/CMAR.S45642

[71] Koksal D, Demirag F, Bayiz H, Ozmen O, Tatci E, Berktas B, et al. The correlation of SUVmax with pathological characteristics of primary tumor and the value of Tumor/ Lymph node SUVmax ratio for predicting metastasis to lymph nodes in resected NSCLC patients. J Cardiothorac Surg. 2013 Apr 4;8:63. doi: 10.1186/1749-8090-8-63

[72] Park SY, Lee HS, Jang HJ, Lee GK, Chung KY, Zo JI. Tumor necrosis as a prognostic factor for stage IA non-small cell lung cancer. Ann Thorac Surg. 2011 Jun;91(6):1668-73. doi: 10.1016/j.athoracsur.2010.12.028

[73] Swinson DE, Jones JL, Richardson D, Cox G, Edwards JG, O'Byrne KJ. Tumour necrosis is an independent prognostic marker in non-small cell lung cancer: correlation with biological variables. Lung Cancer. 2002 Sep;37(3):235-40.

[74] Rasey JS, Wui-Jin K, Evans ML, Peterson LM, Lewellen TK, Graham MM, et al. Quantifying regional hypoxia in human tumors with positron emission tomography of [18F]fluoromisonidazole: a pretherapy study of 37 patients. Int J Radiat Oncol Biol Phys. 1996;36:417-28.

[75] Bollineni VR, Kerner GS, Pruim J, Steenbakkers RJ, Wiegman EM, Koole MJ, et al. PET imaging of tumor hypoxia using 18F-fluoroazomycin arabinoside in stage III-IV non-small cell lung cancer patients. J Nucl Med. 2013 Aug;54(8):1175-80. doi: 10.2967/jnumed.112.115014

[76] Höckel M, Vaupel P. Tumor hypoxia: definitions and current clinical, biologic, and molecular aspects. J Natl Cancer Inst. 2001 Feb 21;93(4):266-76. doi: 10.1093/jnci/93.4.266

[77] Durand RE, Sham E. The lifetime of hypoxic human tumor cells. Int J Radiat Oncol Biol Phys. 1998;42(4):711–5.

[78] Hanahan D, Weinberg RA. Hallmarks of cancer: the next generation. Cell. 2011 Mar 4;144(5):646-74. doi: 10.1016/j.cell.2011.02.013

[79] Rivera C, Rivera S, Loriot Y, Vozenin MC, Deutsch E. Lung cancer stem cell: new insights on experimental models and preclinical data. J Oncol. 2011; 2011:549181. doi: 10.1155/2011/549181

[80] Sterlacci W, Savic S, Fiegl M, Obermann E, Tzankov A. Putative stem cell markers in non-small-cell lung cancer: a clinicopathologic characterization. J Thorac Oncol. 2014 Jan;9(1):41-9. doi: 10.1097/JTO.0000000000000021

[81] Chen YC, Hsu HS, Chen YW, Tsai TH, How CK, Wang CY, et al. Oct-4 expression maintained cancer stem-like properties in lung cancer-derived CD133-positive cells. PLoS One. 2008 Jul 9;3(7):e2637. doi: 10.1371/journal.pone.0002637

[82] Quintana E, Shackleton M, Sabel MS, Fullen DR, Johnson TM, Morrison SJ. Efficient tumour formation by single human melanoma cells. Nature. 2008;456:593–8. doi: 10.1038/nature07567

[83] Ho MM, Ng AV, Lam S, Hung JY. Side population in human lung cancer cell lines and tumors is enriched with stem-like cancer cells. Cancer Res. 2007 May 15;67(10):4827-33.

[84] Levina V, Marrangoni AM, DeMarco R, Gorelik E, Lokshin AE. Drug-selected human lung cancer stem cells: cytokine network, tumorigenic and metastatic properties. PLoS One. 2008 Aug 27;3(8):e3077. doi: 10.1371/journal.pone.0003077

[85] Ishizawa K, Rasheed ZA, Karisch R, Wang Q, Kowalski J, Susky E, et al. Tumor-initiating cells are rare in many human tumors. Cell Stem Cell. 2010 Sep 3;7(3):279-82. doi: 10.1016/j.stem.2010.08.009

[86] Zhang X, Fang B, Mohan R, Chang JY. Coxsackie-adenovirus receptor as a novel marker of stem cells in treatment-resistant non-small cell lung cancer. Radiother Oncol. 2012 Nov;105(2):250-7. doi: 10.1016/j.radonc.2012.09.002

[87] Singh S, Bora-Singhal N, Kroeger J, Laklai H, Chellappan SP. βArrestin-1 and Mcl-1 modulate self-renewal growth of cancer stem-like side-population cells in non-small cell lung cancer. PLoS One. 2013;8(2):e55982. doi: 10.1371/journal.pone.0055982

[88] Xu MH, Gao X, Luo D, Zhou XD, Xiong W, Liu GX. EMT and acquisition of stem cell-like properties are involved in spontaneous formation of tumorigenic hybrids between lung cancer and bone marrow-derived mesenchymal stem cells. PLoS One. 2014 Feb 6;9(2):e87893. doi: 10.1371/journal.pone.0087893

[89] Kelly PN, Dakic A, Adams JM, Nutt SL, Strasser A. Tumor growth need not be driven by rare cancer stem cells. Science. 2007; 317:337.

[90] Liu HG, Zhang XH. How to estimate cancer stem cell frequency correctly. Asian Pac J Cancer Prev. 2009 Oct-Dec;10(4):711-4.

[91] Moore N, Lyle S. Quiescent, Slow-Cycling Stem Cell Populations in Cancer: A Review of the Evidence and Discussion of Significance. J Oncol. 2011;2011. pii: 396076. doi: 10.1155/2011/396076

[92] Jiang F, Qiu Q, Khanna A, Todd NW, Deepak J, Xing L, et al. Aldehyde dehydrogenase 1 is a tumor stem cell-associated marker in lung cancer. Mol Cancer Res. 2009 Mar;7(3):330-8. doi: 10.1158/1541-7786.MCR-08-0393

[93] Hu Y, Smyth GK. ELDA: extreme limiting dilution analysis for comparing depleted and enriched populations in stem cell and other assays. J Immunol Methods. 2009 Aug 15;347(1-2):70-8. doi: 10.1016/j.jim.2009.06.008
