# Supplementary material for: In Silico Oncology: Quantification of the In Vivo Antitumor Efficacy of Cisplatin-Based Doublet Therapy in Non-Small Cell Lung Cancer (NSCLC) through a Multiscale Mechanistic Model
Source: PLoS Comput Biol. 2016 Sep 22;12(9):e1005093. doi: 10.1371/journal.pcbi.1005093 (PMC5033576; doi:10.1371/journal.pcbi.1005093)
Supplement: S4 Text — (PDF) [file pcbi.1005093.s004.pdf]

S4 Text

Range sensitivity analysis

1. Method

The range sensitivity measures (SM) defined here are similar to the local sensitivity measures of the main manuscript; however the inputs are now varied over the anticipated value range [1, 2], instead of assuming a small perturbation around the baseline values. Each input parameter takes its minimum and maximum value and the corresponding change in the calculated CKR, expressed as a percentage of the reference value, is recorded. The rest of the model parameters are kept at their baseline values. All percentage changes in the output are then expressed in relation to ±1% variation of the input, by dividing with the percentage change of the input, according to the formulas:

$$SM_{\max} = \frac{(CKR_{\max} - CKR_{base})/CKR_{base}}{(p_{i,\max} - p_{i,base})/p_{i,base}} \tag{1}$$

$$SM_{\min} = \frac{(CKR_{\min} - CKR_{base})/CKR_{base}}{(p_{i,\min} - p_{i,base})/p_{i,base}} \tag{2}$$

$$SM_{\text{range}} = \frac{SM_{\max} - SM_{\min}}{2} \tag{3}$$

where $p_{i,base}$: the baseline value of the i-th parameter, $p_{i,max}$: the maximum value of the i-th parameter, $p_{i,min}$: the minimum value of the i-th parameter, CKR_{base}: the calculated CKR with all parameters set at their baseline values, CKR_{max}: the calculated CKR with the i-th parameter, only, set at its maximum value, CKR_{min}: the calculated CKR with the i-th parameter, only, set at its minimum value.

The value range of input parameters (Table A) has been derived after taking into consideration the literature (Table 4 of main manuscript), while certain cell proliferation constraints should be satisfied (e.g. positive growth rate, volume doubling time higher than 26 days, percentage of stem cells not exceeding 1%). It should be noted that between the adenocarcinoma (ADC) and squamous cell carcinoma (SCC) different value ranges for some of the parameters are observed (Table A). These boundaries have been derived after applying the aforementioned constraints, and their values depend on the baseline values of the remaining model parameters [3, S2 Text].

2. Results

The range sensitivity measures consider a larger increment compared with their local counterparts and, therefore, are expected to give a different value for parameters having a non-linear effect on the output. In our case, a small deviation from linearity exists for the majority of the input parameters (Fig 3 of manuscript). As a result, a very small divergence between the values of the local and range sensitivity measures is noted (Table B). Looking at the overall sensitivity

(two last columns of Table B), the most remarkable differences are observed for the following parameters:

- T_C, $T_{C,stem}$, $T_{C,LIMP}$: A considerable deviation between the results of the local and the range methods is observed for the SCC case, with the latter method indicating a higher overall sensitivity. For the ADC case a noteworthy deviation exists only when the cell cycle duration of both stem and LIMP cells are varied at once, i.e. for T_C.
- R_{ADiff}: The range method gives a higher overall sensitivity for the ADC case. However a low sensitivity is still indicated by both methods.
- N_{LIMP}: The range method gives a slightly higher overall sensitivity especially for the SCC case. However, a low sensitivity is still indicated by both methods.
- P_{G0toG1}, $P_{G0toG1,stem}$: The range method gives a fairly lower overall sensitivity especially for the ADC case. However, a relatively high sensitivity is indicated by all methods.

Both methods identify the same parameters having a trivial effect on output (overall sensitivity measure <0.1% in Table C). The only noteworthy deviation concerns the parameter R_{ADiff}, as stated above. The local method ranks the parameter as non-sensitive for both the ADC and SCC cases, whereas the range method indicates a low sensitivity for ADC only (Table B).

For the rest of the parameters, having a low to high impact on output (overall sensitivity measure >0.1% in Table C), the local method results almost in the same ranking as the range one. Both methods identify the same parameters as the two most sensitive. Differences observed in the overall ranking order are due to the underestimation of the sensitivity for T_C compared to P_{G0toG1} and $P_{G0toG1,stem}$ by the local method, resulting in a lower rank in respect to the range method.

In summary, the sensitivity results of the local sensitivity measures are rather consistent with the range ones, even though the latter method encompasses the effect of non-linearity.

Table A: Input Parameter Values

	**ADC representative case**			**SCC representative case**		
	$p_{i,base}$	$p_{i,min}$	$p_{i,max}$	$p_{i,base}$	$p_{i,min}$	$p_{i,max}$
$\mathbf{T_C}$	42	18	80[a]	60	19[b]	134
$\mathbf{T_{C,stem}}$	42	18	80[a]	60	18	134
$\mathbf{T_{C,LIMP}}$	42	18	134	60	18	134
$\mathbf{T_{G0}}$	382	90	650[a]	242	90	1200
$\mathbf{T_{G0,stem}}$	382	90	650[a]	242	90	1200
$\mathbf{T_{G0,LIMP}}$	382	90	1200	242	90	1200
$\mathbf{T_N}$	23	1	200	79	1	200
$\mathbf{T_A}$	4	1	25	7	1	25
$\mathbf{R_A}$	0.0003	0	0.00043[a]	0.0001	0	0.00036[a]
$\mathbf{R_{ADiff}}$	0.008	0.001	0.02	0.017	0.001	0.02
$\mathbf{R_{NDiff}}$	0.0009	0	0.02	0.01	0	0.02
$\mathbf{P_{G0toG1}}$	0.5	0.46[a]	0.86[c]	0.1	0.055[a]	0.295[c]
$\mathbf{P_{G0toG1,stem}}$	0.5	0.46[a]	0.86[c]	0.1	0.055[a]	0.295[c]
$\mathbf{P_{G0toG1,LIMP}}$	0.5	0	1	0.1	0	1
$\mathbf{N_{LIMP}}$	18	13[d]	24	22	18[d]	24
**CKF**	0.5	0	1	0.5	0	1
$\mathbf{P_{sym}}$	0.2	0.19[a]	0.292[c]	0.37	0.346[a]	0.449[c]
$\mathbf{P_{sleep}}$	0.265733	0.172[c]	0.281[a]	0.2796	0.234[c]	0.294[a]
$\mathbf{CKR_{dFdC}}$	0.2	0	0.67[e]	0.2	0	0.41[e]

[a] Derived based on the constrain for positive growth rate.
[b] For T_C=18 no solution with the sum of cell kill rates < 1 exists.
[c] Derived based on the constrain for a volume doubling time higher than 26 days.
[d] Derived based on the constrain for a fraction of stem cells lower than 0.001.
[e] No solution exists for parameter values above the considered upper limit. More specifically, treatment shrinks tumor at a higher extent compared to the observed volume reduction for all values of cisplatin's cell kill rate.

Table B: Sensitivity results. Comparison with local sensitivity measures

	-1% parameter variation		+1% parameter variation		overall variation	
	$SM_{-\%}$	SM_{min}	$SM_{+\%}$	SM_{max}	$SM_{\pm\%}$*	SM_{range}
			ADC representative case			
T_C	0.36215	0.55016	-0.20877	-0.19057	-0.28546	-0.37037
$T_{C,stem}$	0.15179	0.16567	-0.07270	-0.07594	-0.11224	-0.12081
$T_{C,LIMP}$	0.28096	0.32963	-0.14030	-0.07492	-0.21063	-0.20228
T_{G0}	0.03331	0.12818	-0.04544	-0.00714	-0.03938	-0.06766
$T_{G0,stem}$	0.12712	0.19014	-0.10131	-0.08587	-0.11421	-0.13801
$T_{G0,LIMP}$	-0.07368	-0.10114	0.08579	0.08308	0.07973	0.092109
T_N	-0.00093	0.00082	0.01839	0.00368	0.00966	0.001427
T_A	0.00083	0.00221	0.00508	0.00013	0.00213	-0.00104
R_A	0.16922	0.15489	-0.17712	-0.17717	-0.17317	-0.16603
R_{ADiff}	-0.00886	0.28591	0.00039	0.00376	0.00463	-0.14107
R_{NDiff}	0.00499	-0.00111	-0.00428	0.00030	-0.00464	0.00071
P_{G0toG1}	-0.92662	-0.92061	0.80399	0.56740	0.85849	0.74401
$P_{G0toG1,stem}$	-0.86893	-0.8829	0.77345	0.56509	0.81589	0.72400
$P_{G0toG1,LIMP}$	-0.03796	-0.03497	0.04177	0.04681	0.03987	0.04089
N_{LIMP}	0.20374	0.28702	-0.13113	-0.09726	-0.16743	-0.19214
CKF	0.02495	0.03783	-0.04721	-0.03718	-0.03608	-0.03751
P_{sym}	-1.03013	-1.03483	1.03419	0.88703	1.03216	0.96093
P_{sleep}	1.00887	1.13015	-0.98271	-1.00150	-0.99579	-1.06583
CKR_{dFdC}	-0.07704	-0.07735	0.06311	0.06376	0.07007	0.07055
			SCC representative case			
T_C	0.38847	1.45248	-0.29436	-0.14327	-0.34141	-0.79788
$T_{C,stem}$	0.19777	0.45636	-0.16914	-0.09868	-0.18345	-0.27752
$T_{C,LIMP}$	0.17016	0.72548	-0.12011	-0.03999	-0.14513	-0.38274
T_{G0}	-0.07892	-0.07947	0.08079	0.09267	0.07985	0.08607
$T_{G0,stem}$	0.01392	0.02635	-0.03329	-0.01606	-0.02361	-0.02120
$T_{G0,LIMP}$	-0.10306	-0.10771	0.11052	0.10377	0.10679	0.10574
T_N	-0.00373	-0.00588	0.01612	0.03688	0.00993	0.02138
T_A	0.00478	0.00158	0.00700	-0.00026	0.00111	-0.00092
R_A	0.07715	0.06257	-0.05874	-0.06823	-0.06795	-0.06540
R_{ADiff}	-0.00884	-0.00337	0.00498	0.00741	0.00691	0.00539
R_{NDiff}	-0.00022	-0.00027	0.00667	0.00067	0.00344	0.00047
P_{G0toG1}	-0.36875	-0.40018	0.34977	0.23344	0.36875	0.31681
$P_{G0toG1,stem}$	-0.31340	-0.35189	0.30110	0.21309	0.30725	0.28249
$P_{G0toG1,LIMP}$	-0.04049	-0.04802	0.04482	0.02115	0.04266	0.03458
N_{LIMP}	0.15648	0.25710	-0.06639	-0.07749	-0.11144	-0.16730
CKF	0.02646	0.01629	-0.00694	-0.01271	-0.02646	-0.01450
P_{sym}	-2.57337	-2.61168	2.27842	1.94370	2.42590	2.27769
P_{sleep}	2.73239	2.37882	-3.11203	-3.10590	-2.92221	-2.74236
CKR_{dFdC}	0.12396	0.10765	-0.14345	-0.16382	-0.13370	-0.13573

* $SM_{\pm\%} = (SM_{+\%} - SM_{-\%})/2$

Table C: Parameter ranking derived based on the overall sensitivity

$SM_{\pm\%}$		SM_{range}	
**Parameter***	**Sensitivity‡**	**Parameter***	**Sensitivity‡**
P_{sleep}	1.95900	P_{sleep}	1.90410
P_{sym}	1.72903	P_{sym}	1.61931
P_{G0toG1}	0.61362	T_C	0.58413
$P_{G0toG1,stem}$	0.56157	P_{G0toG1}	0.53041
T_C	0.31344	$P_{G0toG1,stem}$	0.50324
$T_{C,LIMP}$	0.17788	$T_{C,LIMP}$	0.29251
$T_{C,stem}$	0.14785	$T_{C,stem}$	0.19917
N_{LIMP}	0.13944	N_{LIMP}	0.17972
R_A	0.12056	R_A	0.11572
CKR_{dFdC}	0.10189	CKR_{dFdC}	0.10314
$T_{G0,LIMP}$	0.09326	$T_{G0,LIMP}$	0.09892
$T_{G0,stem}$	0.06891	$T_{G0,stem}$	0.07961
T_{G0}	0.05962	T_{G0}	0.07687
$P_{G0toG1,LIMP}$	0.04126	R_{ADiff}	0.07323
CKF	0.03127	$P_{G0toG1,LIMP}$	0.03774
T_N	0.00979	CKF	0.02601
R_{ADiff}	0.00577	T_N	0.0114
R_{NDiff}	0.00404	T_A	0.00098
T_A	0.00162	R_{NDiff}	0.00059

* Sorted from high to low sensitivity.
‡ Derived by averaging the absolute of the overall sensitivity measure for ADC and SCC.

References

[1] U.S. EPA. TRIM, Total Risk Integrated Methodology, TRIM FaTE Technical Support Document Volume I: Description of Module. Office of Air Quality Planning and Standards. 2002. EPA-453/R-02-011a. Available in http://nepis.epa.gov/Adobe/PDF/2000NRID.PDF (last visited on 1 July 2016)

[2] U.S. EPA. Risk Assessment Guidance for Superfund: Volume III - Part A, Process for Conducting Probabilistic Risk Assessment. 2001. EPA 540-R-02-002. Available in https://www.epa.gov/sites/production/files/2015-09/documents/rags3adt_complete.pdf (last visited on 1 July 2016)

[3] Kolokotroni EA, Dionysiou DD, Uzunoglu NK, Stamatakos GS. Studying the growth kinetics of untreated clinical tumors by using an advanced discrete simulation model. Math Comput Model. 2011;54:1989-2006. doi:10.1016/j.mcm.2011.05.007
