# Supplementary material for: In Silico Oncology: Quantification of the In Vivo Antitumor Efficacy of Cisplatin-Based Doublet Therapy in Non-Small Cell Lung Cancer (NSCLC) through a Multiscale Mechanistic Model
Source: PLoS Comput Biol. 2016 Sep 22;12(9):e1005093. doi: 10.1371/journal.pcbi.1005093 (PMC5033576; doi:10.1371/journal.pcbi.1005093)
Supplement: S5 Text — (PDF) [file pcbi.1005093.s005.pdf]

Eleni Kolokotroni, Dimitra Dionysiou, Christian Veith, Yoo-Jin Kim, Jörg Sabczynski, Astrid Franz, Aleksandar Grgic, Jan Palm, Rainer M. Bohle, Georgios Stamatakos

S5 Text

Value range of spontaneous apoptosis rate

Based on Eq. (8) in Kolokotroni *et al.* (2011), the apoptosis rate of stem and LIMP cells, R_A, must satisfy the following inequality in order to have a stable or growing tumor:

$$e^{R_A T_C} \leq (1 + P_{sym})(1 - P_{sleep} + P_{sleep}\frac{P_{G0toG1}/T_{G0}}{R_A + 1/T_{G0}}) \tag{1}$$

The second term of the above inequality is maximized for the maximum value of P_{sym}, i.e. 0.4 (the upper bound of this parameter considered in the present work) and the minimum value of P_{sleep}, i.e. 0. By substituting the above values in Eq (1), and after performing some derivations, the upper bound of R_A for a given cell cycle duration, T_C, can be derived, above which no solution, i.e. set of parameter values that gives a stable or growing tumor, exists:

$$R_A \leq \frac{\ln 1.4}{T_c} \tag{2}$$

Let [18, 134] be the considered value range of T_C. When R_A is below the lower bound of the second term in Eq (2), i.e. ln1.4/134≈0.0025h^{-1}, a solution exists for all values of T_C considered. In the present work R_A has been assumed to vary between 0 and 0.001 h^{-1}.

References

Kolokotroni EA, Dionysiou DD, Uzunoglu NK, Stamatakos GS. Studying the growth kinetics of untreated clinical tumors by using an advanced discrete simulation model. Math Comput Model. 2011;54:1989-2006. doi:10.1016/j.mcm.2011.05.007
