# Supplementary figures and images for: In Silico Oncology: Quantification of the In Vivo Antitumor Efficacy of Cisplatin-Based Doublet Therapy in Non-Small Cell Lung Cancer (NSCLC) through a Multiscale Mechanistic Model
Source: PLoS Comput Biol. 2016 Sep 22;12(9):e1005093. doi: 10.1371/journal.pcbi.1005093 (PMC5033576; doi:10.1371/journal.pcbi.1005093)

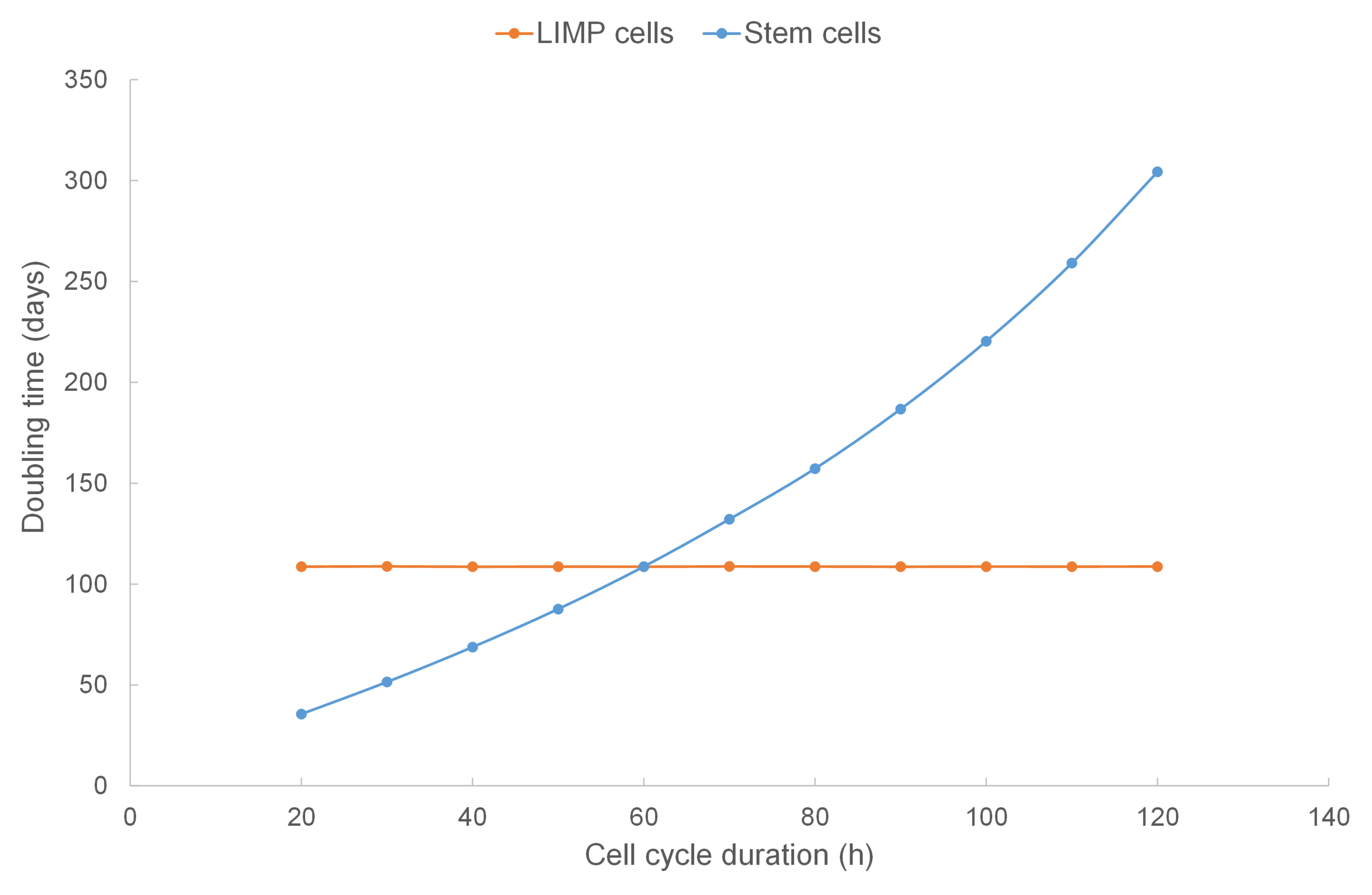

Supplement: S1 Fig — The values assigned to the rest of the model input parameters correspond to the baseline values of SCC (Table 5). Abbreviations: LIMP: LImited Mitotic Potential tumor cell (also called committed or restricted progenitor cell), SCC: Squamous Cell Carcinoma. (TIF) [file pcbi.1005093.s006.tif]

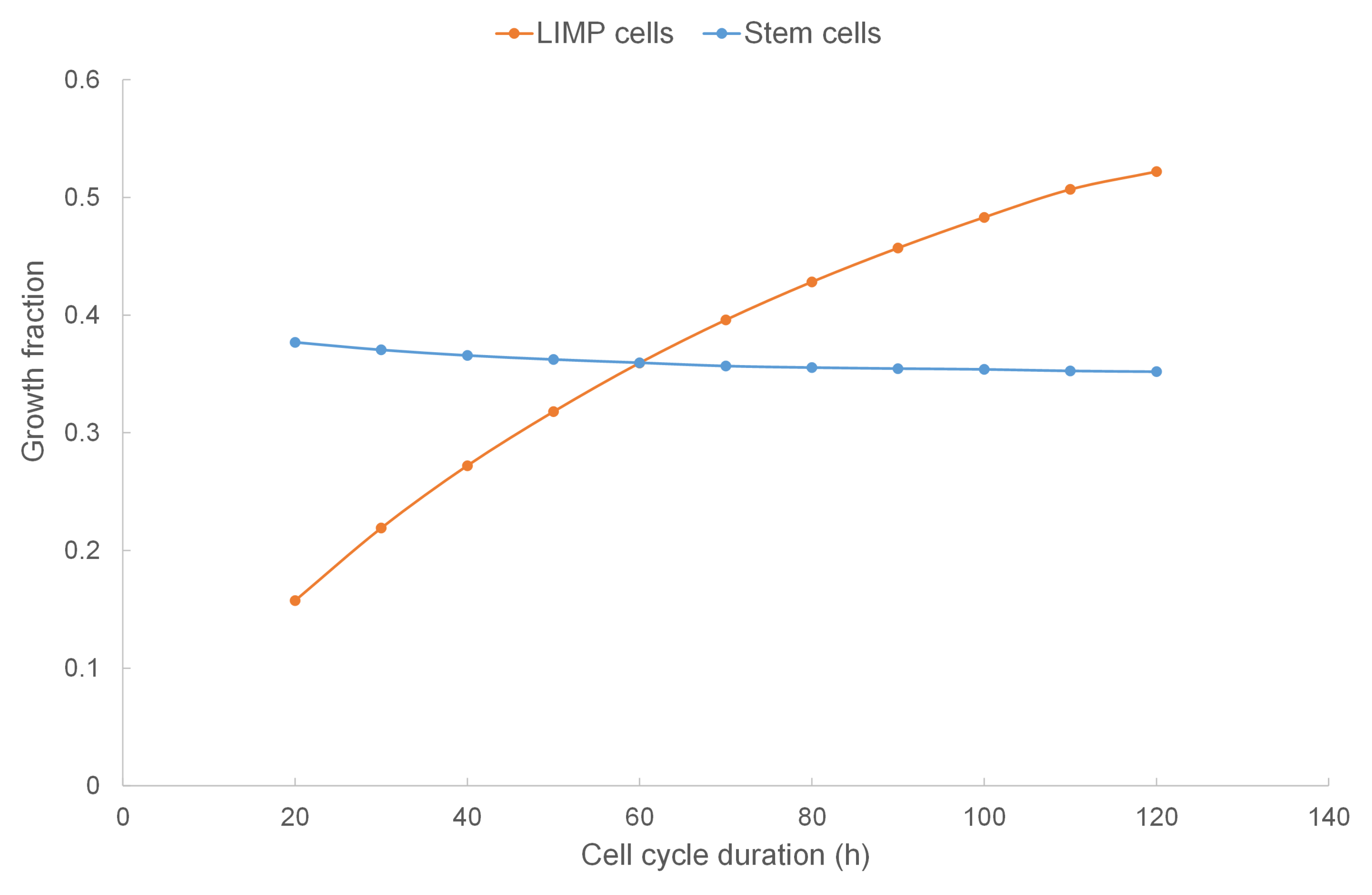

Supplement: S2 Fig — The values assigned to the rest of the model input parameters correspond to the baseline values of SCC (Table 5). Abbreviations: LIMP: LImited Mitotic Potential tumor cell (also called committed or restricted progenitor cell), SCC: Squamous Cell Carcinoma. (TIF) [file pcbi.1005093.s007.tif]

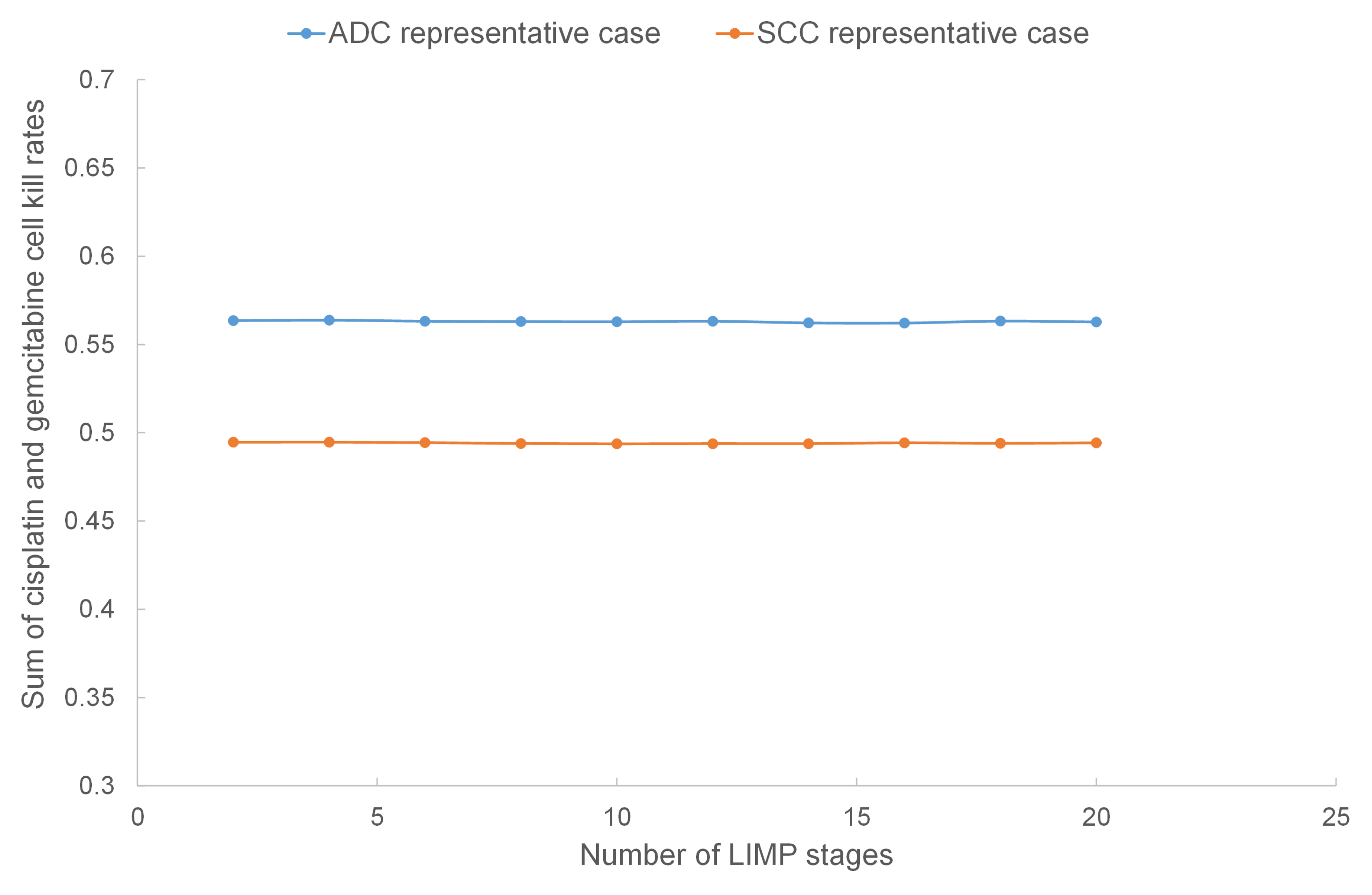

Supplement: S3 Fig — The rest of the model parameters are kept constant at a baseline value. Stem and LIMP cells are assumed to be equally sensitive to treatment, i.e. the cell kill factor of stem cells, CKF, is set equal to unity. Two sets of baseline values have been considered for the rest of the model input parameters, corresponding to a SCC and an ADC representative case (Table 5). Abbreviations: LIMP: LImited Mitotic Potential tumor cell (also called committed or restricted progenitor cell), ADC: Adenocarcinoma, SCC: Squamous Cell Carcinoma. (TIF) [file pcbi.1005093.s008.tif]

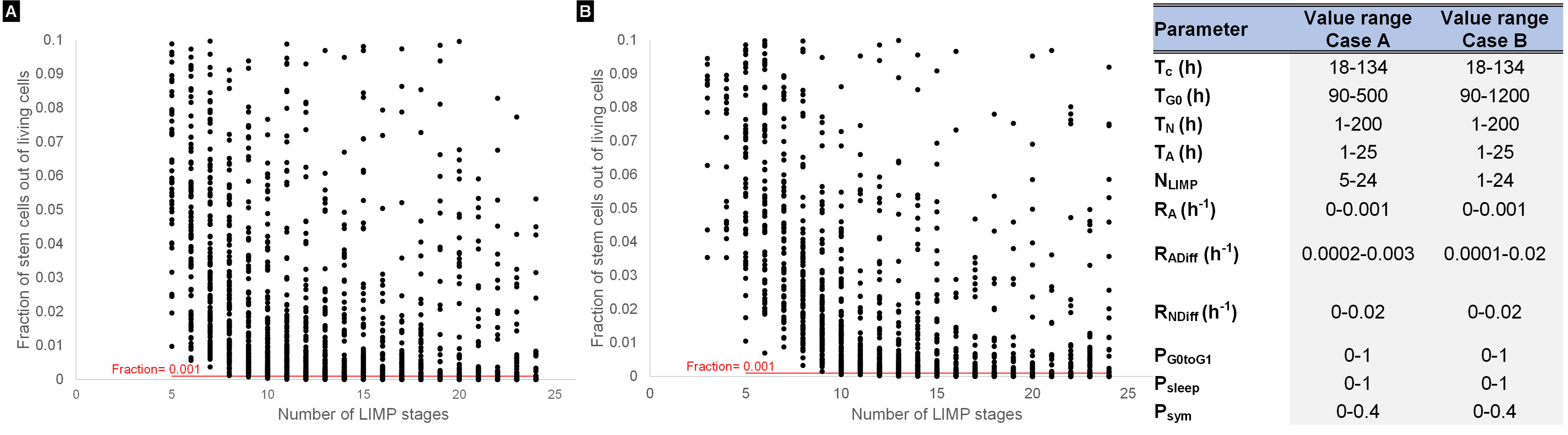

Supplement: S4 Fig — Latin Hypercube Sampling has run to produce two sets of 8000 combinations of model parameters (Table 4), with the given value ranges (cases A and B respectively). Combinations with negative cell proliferation kinetics have been excluded. The red line corresponds to the upper limit of 0.001 considered in the present study for the cancer stem cell fraction. We observe that for values of NLIMP lower than 8, this limit is always exceeded. Abbreviations: LIMP: LImited Mitotic Potential tumor cell (also called committed or restricted progenitor cell). (TIF) [file pcbi.1005093.s009.tif]
