# Supplementary material for: In Silico Oncology: Quantification of the In Vivo Antitumor Efficacy of Cisplatin-Based Doublet Therapy in Non-Small Cell Lung Cancer (NSCLC) through a Multiscale Mechanistic Model
Source: PLoS Comput Biol. 2016 Sep 22;12(9):e1005093. doi: 10.1371/journal.pcbi.1005093 (PMC5033576; doi:10.1371/journal.pcbi.1005093)
Supplement: S1 Table — (PDF) [file pcbi.1005093.s010.pdf]

Eleni Kolokotroni, Dimitra Dionysiou, Christian Veith, Yoo-Jin Kim, Jörg Sabczynski, Astrid Franz, Aleksandar Grgic, Jan Palm, Rainer M. Bohle, Georgios Stamatakos

S1 Table

Supplemental PRCC analyses

Analysis A			Analysis B		
**Feature**	**PRCC**	**p-value**	**Feature**	**PRCC**	**p-value**
Stem/LIMP	0.129	$2.47*10^{-3}$	Stem/DIFF	0.113	$7.97*10^{-3}$
LIMP/Living	0.131	$2.11*10^{-3}$	LIMP/Living	0.111	$9.08*10^{-3}$
Growth Fraction	-0.487	$6.00*10^{-34}$	Growth Fraction	-0.489	$3.73*10^{-34}$
Quiescent/Living	-0.0823	0.0544	Quiescent/Living	-0.0845	0.0482
Apoptotic/Total	0.0187	0.663	Apoptotic/Total	0.0184	0.667
Necrotic/Total	0.121	$4.76*10^{-3}$	Necrotic/Total	0.120	$4.90*10^{-3}$
T_d	-0.813	$2.62*10^{-130}$	T_d	-0.813	$2.20*10^{-130}$

Values rounded to three significant figures

DIFF: terminally differentiated tumor cell, LIMP: LImited Mitotic Potential tumor cell, T_d: volume doubling time
